# Total neoadjuvant treatment using short-course radiotherapy and four CAPOX cycles in locally advanced rectal cancer with high-risk criteria for recurrence: a Swedish nationwide cohort study (LARCT-US)

**DOI:** 10.1016/j.eclinm.2024.102771

**Published:** 2024-08-05

**Authors:** Bengt Glimelius, Tanweera Khan, Karin Adolfsson, Eva Angenete, Åke Berglund, Kristina Bonde, Nils Elander, Tone Fokstuen, Johan Haux, Israa Imam, Cecilia Lagerbäck, Ingrid Ljuslinder, Andrzej Piwowar, Marie Zajicova, Per J. Nilsson

**Affiliations:** a Department of Immunology, Genetic and Pathology, Uppsala University, Uppsala, Sweden; b Department of Oncology, Ryhovs Hospital, Jönköping, Sweden; c Department of Surgery, SSORG - Scandinavian Surgical Outcomes Research Group, Institute of Clinical Sciences, Sahlgrenska Academy, University of Gothenburg, Gothenburg, Sweden; d Region Västra Götaland, Sahlgrenska University Hospital, Department of Surgery, Gothenburg, Sweden; e Department of Oncology, Falun Hospital, Falun, Sweden; f Department of Oncology, Central Hospital, Karlstad, Sweden; g Department of Oncology, University Hospital, Linköping, Sweden; h Department of Oncology, Karolinska University Hospital, Stockholm, Sweden; i Department of Oncology Skaraborgs Hospital, Skövde, Sweden; j Department of Oncology, Södersjukhuset, Stockholm, Sweden; k Department of Radiation Sciences, Oncology, University Hospital of Umeå, Umeå, Sweden; l Department of Oncology, Västmanlands Hospital, Västerås, Sweden; m Department of Oncology, Gävle Hospital, Gävle, Sweden; n Department of Pelvic Cancer, Division Coloproctology, Karolinska University Hospital, Stockholm, Sweden

**Keywords:** Locally advanced rectal cancer, Total neoadjuvant treatment, Complete response, Population observational cohort

## Abstract

**Background:**

Total neoadjuvant treatment (TNT) for locally advanced rectal cancer (LARC) increases pathologic complete response (pCR) rate and reduces the risk of systemic recurrences over chemoradiotherapy (CRT) in randomised trials, e.g., the RAPIDO trial. A modified RAPIDO schedule was prospectively explored in Sweden to evaluate TNT in routine health care before the RAPIDO results were published.

**Methods:**

Between July 2016 and June 2020, 273 patients with high-risk LARC (clinical tumour stage cT4, clinical nodal stage cN2, extramural vascular invasion, involved mesorectal fascia or enlarged lateral lymph nodes) were treated in a prospective observational cohort study at 16 hospitals (LARCT-US). Another 189 patients at 18 (including the 16) hospitals were similarly treated (*ad modum* LARCT-US, AdmL) during the same period. Inclusion and exclusion criteria were identical to the RAPIDO trial. Patients received short-course radiotherapy (5 × 5 Gy for 5 days) followed by four cycles of CAPOX or six FOLFOX-6, followed by total mesorectal excision or, if clinical complete response (cCR), inclusion into a watch-and-wait (W&W) study. The primary endpoint was complete response (CR), i.e., the sum of pCR in specimens and cCR exceeding one year in W&W patients. Safety was assessed in all patients.

**Findings:**

Compared to the RAPIDO trial, patients were older, and tumours more advanced. Median follow-up was 4.8 years (IQR 4.2–5.2). In LARCT-US all patients received radiotherapy and 268 (98%) started chemotherapy whereas in AdmL all patients received radiotherapy and chemotherapy. In LARCT-US 34 patients had pCR and 31 sustained cCR resulting in a CR-rate of 24% (95% CI 20–28). In AdmL, results were similar (23%, 95% CI 17–30). Locoregional recurrences were 6% (95% CI 4–10) and 5% (95% CI 2–9), respectively, both at 3 years and at last follow-up. Neurotoxicity, recorded in LARCT-US, was lower than in RAPIDO (EORTC-QLQ-CIPN20 tingling toes or feet mean score 24 (SD 31) vs 43 (SD 37)). One treatment-associated death occurred.

**Interpretation:**

Despite older patients and more advanced tumours, results similar to the RAPIDO trial were obtained. Hence, two chemotherapy cycles less do not compromise the results maintaining a high CR-rate. This TNT schedule resulted in favourable outcomes in a nation-wide real-life situation.

**Funding:**

10.13039/501100002794Swedish Cancer Society.

**Research in context**

**Evidence before this study**

On December 5, 2023, we searched PubMed, without any language restrictions, using terms related to locally advanced rectal cancer, radiotherapy, preoperative chemotherapy, and total neoadjuvant treatment (TNT). When this cohort study was initiated, two randomised trials had used 5 × 5 Gy radiotherapy followed by preoperative chemotherapy for 6 and 12 weeks, respectively. The latter study had reported short-term results in the first portion of included patients. Research during the past decades had found that total mesorectal excision (TME), often preceded by either short-course radiotherapy or concomitant chemoradiotherapy, had resulted in low locoregional recurrence rates in rectal cancers considered locally advanced by MRI but the risk of distant metastases and mortality had not substantially decreased. Several randomised trials exploring adjuvant chemotherapy failed to show convincing evidence of reduced recurrence risks or improved survival. Despite this, adjuvant chemotherapy was widely used, however, often with poor compliance. The RAPIDO trial had just completed patient enrollment after having randomised 920 patients with locally advanced rectal cancer and high-risk criteria for recurrence between TNT using 5 × 5 Gy radiotherapy and 18 weeks of chemotherapy and chemoradiotherapy with optional adjuvant chemotherapy. Most Swedish hospitals that included patients in RAPIDO decided to use the same schedule of TNT but with a reduction in chemotherapy to only 12 weeks as reference treatment while awaiting the results of the RAPIDO trial. One reason for abbreviating the chemotherapy was the anticipation that three months of adjuvant chemotherapy in colon cancer was as effective as six months, which was later confirmed from an analysis of several randomised trials (the IDEA consortium).

**Added value of this study**

The RAPIDO trial reported after three years minimum follow-up that TNT reduced the rate of disease-related treatment failure, mainly due to fewer distant metastases, without increasing the risk of locoregional failures. TNT also doubled the chance of pathological complete response and was neither more toxic nor resulted in poorer quality of life compared to chemoradiotherapy. During the four years prior to the RAPIDO results being reported, 462 patients from 5/6 Swedish health care regions were treated according to this new reference schedule, either after informed consent (n = 273, LARCT-US) or similarly in routine care (*ad modum* LARCT-US, AdmL, n = 189). The RAPIDO protocol was adhered to apart from randomization and, in good-responders, surgery could be deferred using a watch-and-wait protocol (W&W). After comparable follow-up, the results of the experimental arm of RAPIDO were replicated, i.e., high complete response rates (complete pathological and clinical response sustained for more than one year) with less neurological problems. The results were achieved despite that more advanced tumours and older patients were treated than in the RAPIDO trial. Loco-regional recurrence rates were also lower than reported in RAPIDO and distant metastasis rates were similar.

**Implications of all the available evidence**

Several randomised phase II or III trials have reported that TNT results in higher rates of complete response, fewer distant metastases, and improved disease-free survival. However, improved overall survival has been elusive. Various TNT schedules have been used and some compared with each other, but no reference treatment has yet emerged. The RAPIDO trial reported a maintained reduction in distant metastases after five years but an increased risk of locoregional failures causing concern. Consequently, some have started to disfavour short-course radiotherapy in favour of long-course chemoradiotherapy. However, aggregating the results of the Polish and the Chinese STELLAR trials with the low locoregional recurrence rates reported from this nationwide “real-life” cohort study confirms that TNT including short-course radiotherapy is effective. Furthermore, advantages for the patients and healthcare system resource utilization are evident. The increased locoregional recurrence rate after longer follow-up in RAPIDO remains to be explained but it can be hypothesized that for non- or poorly responding patients, 18 weeks of chemotherapy prior to surgery may be too long. Twelve weeks appears to be sufficient to obtain high response rates of importance if organ preservation is a primary aim.

## Introduction

In patients with locally advanced rectal cancer (LARC), preoperative chemoradiotherapy (CRT) has been the reference treatment for two decades.^1^^,^^2^ Frequently, adjuvant chemotherapy is administered despite limited evidence of improved disease-free and overall survival (DFS and OS).^3^^,^^4^ CRT ± adjuvant chemotherapy together with a total mesorectal excision (TME) have resulted in low locoregional recurrence rates (LRR, about 5–10%) but without significant impact on distant metastases (DM) or OS.^5^ This, and suboptimal compliance to adjuvant chemotherapy attracted interest in delivering neoadjuvant systemic treatment. Randomised phase III trials using this approach, total neoadjuvant treatment (TNT), have reported fewer DM, improved DFS or disease-related treatment failure (DrTF) and higher pathologic complete response (pCR),^6^^,^^7^ albeit not in all trials.^8^^,^^9^ Different inclusion criteria and TNT schedules were used, and the optimal regimen is unknown.^10^

The RAPIDO trial^6^ compared CRT with TNT using short-course radiotherapy (scRT, 5 Gy x 5 for 5 days) followed by six cycles of CAPOX (capecitabine/oxaliplatin) or nine FOLFOX (5-fluorouracil/leucovorin/oxaliplatin) preoperatively. In CRT patients, adjuvant chemotherapy was optional. Patient accrual closed in June 2016 at 920 randomised patients. Uppsala, the largest recruiting centre, perceived positive experiences with the TNT including a logistically advantageous reduction of radiation fractions (5 vs 25–28), CAPOX tolerability, and occurrences of complete responses (CRs), and decided to continue TNT while awaiting the RAPIDO results. It was then decided to modify the TNT regimen and abbreviate the number of chemotherapy cycles for the following two reasons: (i) the anticipation that abbreviation of adjuvant chemotherapy in colon cancer stage III should be non-inferior (IDEA consortium^11^), and (ii) reports of pCR of 26% using scRT followed by four cycles of CAPOX in LARC.^12^ Thus, a modified RAPIDO schedule with four cycles of CAPOX as reference treatment was introduced (locally advanced rectal cancer treatment–Uppsala style, LARCT-US) and ethical approval was obtained.

Many Swedish centres joined the study but some lacked resources to prospectively include patients but treated patients in accordance with the protocol “off-study”. Because all patients are registered in the Swedish Colorectal Cancer Registry (SCRCR), outcomes for patients treated *ad modum* LARCT-US (AdmL) were available for analysis.

We aim to report the results of all Swedish LARC patients treated with an abbreviated RAPIDO TNT schedule, while waiting for the presentation of the RAPIDO trial results, after minimum 3½-years follow-up. Results are primarily compared with those of the experimental arm of RAPIDO.^6^

## Methods

Sixteen hospitals joined the prospective LARCT-US study, however, at some centres, some patients were not formally included and, thus, became AdmL patients. Two non-participating hospitals treated patients AdmL. During the covid-19 pandemic, study inclusion was temporarily halted at most centres, but treatment AdmL continued. Collectively, these 18 hospitals (Appendix p 2) constituted all hospitals treating LARC in 5/6 Swedish health care regions.

### Staging and treatment

Patients with rectal adenocarcinoma less than 16 cm from the anal verge were staged with pelvic magnetic resonance imaging (MRI), as in the RAPIDO protocol, and thoracoabdominal computed tomography (CT) prior to a multidisciplinary team (MDT) conference. To facilitate comparison with the RAPIDO trial, identical criteria (at least one risk criterium on MRI: cT4, cN2, involved mesorectal fascia (MRF+), extramural vascular invasion (EMVI+), or enlarged lateral lymph nodes (LN+)) were used. Staging, exclusion criteria and follow-up were identical. Patients should have adequate blood counts, no contraindications to treatment, be above 18 years, an Eastern Cooperative Oncology Group (ECOG) performance status ≤1, and adequate potential for follow-up. In LARCT-US, written informed consent was required. Extensive growth into the cranial part of the sacrum or the lumbosacral nerve roots indicating irresectability, DM or recurrent rectal cancer, familial adenomatosis polyposis, Lynch Syndrome, active Crohn's disease or active ulcerative colitis, concomitant malignancies (subjects with prior malignancies should be disease-free for at least 5 years), known dihydropyrimidine dehydrogenase (DPD) deficiency, contraindications to MRI, concurrent uncontrolled medical conditions, any investigational treatment for rectal cancer within the past month, pregnancy or breast feeding, known malabsorption syndromes, clinically significant cardiac disease or myocardial infarction within the past 12 months and symptoms or history of peripheral neuropathy were exclusion criteria.

Initial treatment was external radiotherapy to 5 Gy for five consecutive days (week 1). A boost of two-three 2.0 Gy fractions was permitted. The 12-week CAPOX (capecitabine 1000 mg/m^2^ twice daily for 14 days and oxaliplatin 130 mg/m^2^ day 1) should preferably be initiated within 11–18 days (week 3), but no upper limit was defined. Alternatively, modified FOLFOX-6 (oxaliplatin 85 mg/m^2^ day 1, folinic acid 200 mg/m^2^ day 1 followed by bolus fluorouracil 400 mg/m^2^ and fluorouracil 2400 mg/m^2^ for 46 h days 1–2) could be given; the decision to start with FOLFOX-6 was up to the discretion of the treating physician as was the case also in the RAPIDO trial. Dose reductions due to toxicity followed conventional rules.

Tumour response evaluation including digital rectal examination, endoscopy, and pelvic MRI and thoracoabdominal CT should be performed 1–2 weeks after completion of chemotherapy with an MDT the following week. Surgery was performed within the following fortnight (weeks 17–20) unless an excellent response was achieved making organ preservation potentially possible. Contrary to the RAPIDO trial, patients with clinical complete response (cCR) could enter a watch-and-wait (W&W) protocol (clinicaltrials.gov NCT03125343). Surgery was according to TME-principles and extended surgery (“beyond TME”) was performed if indicated.

Postoperatively, follow-up consisted of serum carcinoembryonic antigen (CEA) and thoracoabdominal CT after 12 and 36 months according to Swedish guidelines.^13^ Patients entering the nationwide W&W protocol were followed using pelvic MRI, CEA, and flexible endoscopy every three months for two years and six-monthly thereafter. In addition, CT scans were performed at 12 and 36 months.

### Endpoints

The primary endpoint was complete response (CR), being the sum of pCR in operated patients and sustained cCR (duration >12 months after the start of radiotherapy, s-cCR) in patients where surgery was not primarily done, i.e., W&W patients. Secondary endpoints were acute toxicity according to the Common Terminology Criteria for Adverse Events (CTCAE) version 4.0, DrTF, defined as any recurrence, a new primary colorectal cancer or treatment related death, DFS (any recurrence, a new primary cancer, all deaths), OS (any death irrespective of cause), DM, LRF (any unresectable tumour, local R2-resection and any LRR in R0/R1-resected tumour) and LRR (locoregional recurrence in R0/R1, M0 resected tumours) rates, the neoadjuvant rectal score (NAR)^14^ and quality-of-life (QoL) after three years. Since the NAR-score was developed for operated patients, W&W patients with no regrowth within one year were assigned a low score (0–6.7). For the study to be considered positive, CR rate should exceed that of CRT alone in similar patients (12–14%), be similar to TNT in RAPIDO (29% in the intention-to-treat, ITT, population), and median NAR score below 15.

### Toxicity and quality-of-life (QoL) evaluations

The CTCAE, version 4.0 was used; however, only grade 3+ toxicity was recorded in the case registration forms (CRFs). In SCRCR, only postoperative complications according to the Clavien-Dindo classification were recorded.

For QoL assessment, identical questionnaires as in RAPIDO were applied^15^ at three years after surgery/end of treatment if W&W. Peripheral sensory neurotoxicity was evaluated using EORTC QLQ-CIPN20.^16^ Since the three-year evaluation coincided with the covid-19 pandemic, resulting in closure of most trial units, distribution of questionnaires was frequently postponed, and questionnaires completed between three and five years were accepted. Besides neurotoxicity, other QoL data will be reported separately.

### Statistical analyses and sample size determination

Analyses of the primary endpoint and the secondary endpoints OS, DrTF and locoregional failure (LRF) were in the eligible ITT-population. The secondary endpoints DFS, LRR, and DM were analysed in patients with an R0/R1 resection or entered W&W; surgical complications in patients operated electively with curative intent; QoL in resected patients or entered W&W and remained disease-free; and toxicity in all patients who started treatment. All calculated median values are accompanied by an IQR and means with SDs. 95% confidence intervals (CI) were calculated for proportions using the Clopper-Pearson approach. Since the true population is not known, the conservative Clopper-Pearson approach was considered adequate. All outcome analyses (OS, DFS, time to DrTF, LRF, DM, and LRR) were conducted using the Kaplan–Meier approach. The cumulative incidence of recurrence was computed accounting for death as competing risk. Patients who were alive and disease-free at last follow-up were censored. For CR, odds ratios with the corresponding 95% CIs were calculated. In all analyses we used the current packages in R: survival version 3.5.5, survminer version 0.4.9, epitools version 0.5.10.1, and DescTools version 0.99.53.

**Fig. 1:**
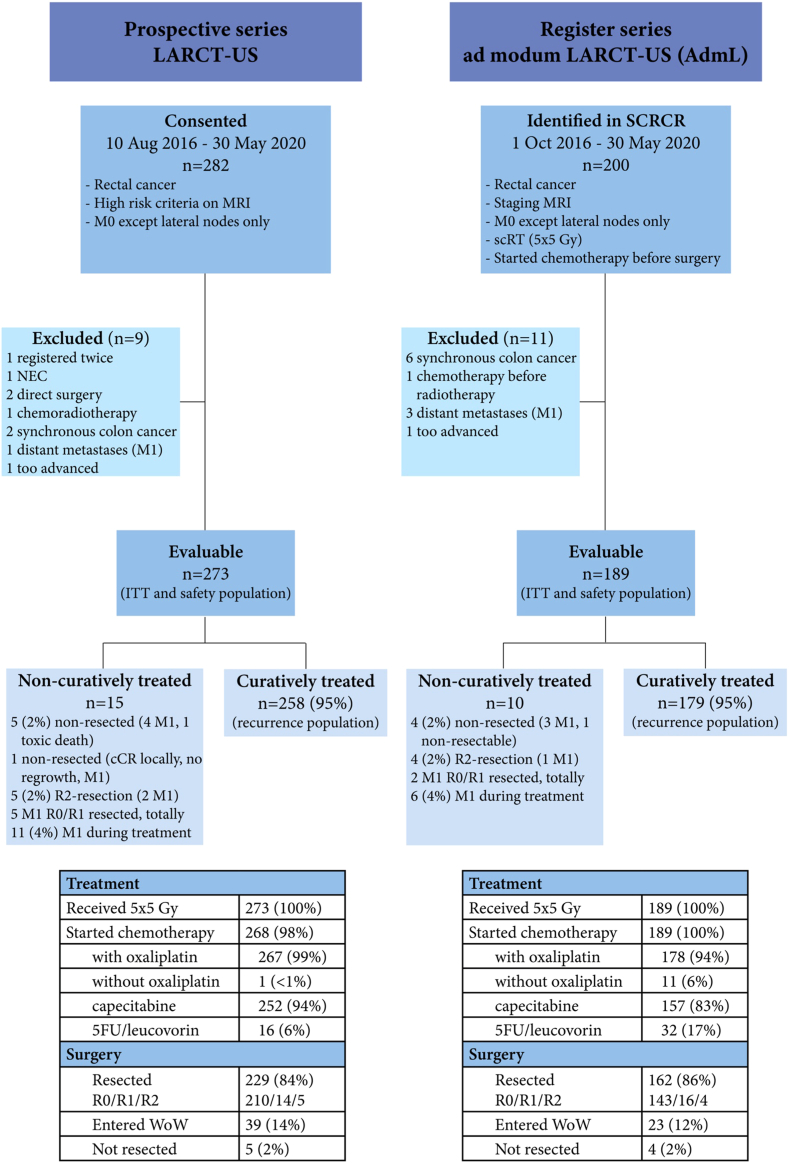
Flow chart.

When patient recruitment to the ethically approved LARCT-US observational study started at three hospitals (population 0.7 million), we estimated that about 60 patients could be treated until the results of the RAPIDO trial were reported after about 3 years. Many other Swedish hospitals started to treat their patients similarly and most of them gradually joined the prospective study. Some hospitals declared that they would treat their patients similarly but not seek informed consent and not complete any CRFs. Ethical approval to retrospectively evaluate all patients in Sweden with LARC and high-risk criteria treated with the LARCT-US schedule (ad modum LARCT-US or AdmL) and registered in the SCRCR until the results of the RAPIDO trial were released (early June 2020) was obtained. The totally 482 identified patients (Flowchart, Fig. 1) constitute all patients thus treated in Sweden during this almost four-year period. Since the patient cohort constitutes all patients in a defined population during a defined time period, no estimates of the number of patients or power calculations were done. Based upon the incidence of rectal cancer in Sweden and the eligible proportion, i.e., having any of the risk criteria, between 100 and 150 patients could be treated per year in five out of six Swedish health care regions (population 8 million) (see also Appendix p 2).

### Ethical approval

The research ethics committee at Uppsala University approved the LARCT-US protocol as research (Dnr 2016/305) with an aim to report the results whereas the Swedish Medical Products Agency (MPA) considered this a reference treatment not requiring their approval. The approved protocol was a minimally modified RAPIDO protocol. The written patient information was modified, and patients provided informed consent prior to treatment. An amendment concerned the NAR score^14^ as a secondary endpoint (Dnr 2016/305/6).

In a separate application, the ethics committee approved a retrospective evaluation of data from SCRCR on all patients treated with this schedule during the period after the last patient had been included in RAPIDO and the first presentation of the results of the RAPIDO trial (Dnr 2016/305/9). SCRCR comprises limited information compared to the CRFs of LARCT-US, however, retrieval of missing information from treating hospitals was approved. When approval to evaluate all patients thus treated in 5/6 Swedish health care regions was obtained, the study was registered at clincialtrials.gov (NCT03729687). The reporting of the study followed the Strengthening the reporting of observational studies in epidemiology, STROBE, statement.

### Role of the funding source

Study design, data collection, analysis, interpretation, and writing of the report were independent of the funder.

## Results

### Patients and neo-adjuvant treatments

Between August 2016 and May 31, 2020, 282 patients gave informed consent to participate in LARCT-US and 200 patients were treated with scRT followed by chemotherapy and registered in SCRCR (AdmL). Twenty patients were ineligible as presented in the flowchart (Fig. 1) leaving 462 patients (273 LARCT-US and 189 AdmL) for analysis. Follow-up on February 6, 2024, ranged between 3.6 and 7.6 years, median 4.9 (IQR 4.3–5.2) in LARCT-US (one recurrence-free patient emigrated after 3.5 years) and 4.8 (IQR 3.9–5.8) in AdmL. Fifty (18%) patients had died in LARCT-US and 37 (20%) in AdmL, most commonly from rectal cancer.

Patient and tumour characteristics (Table 1) were similar between LARCT-US and AdmL. Performance status was not registered in SCRCR, and data regarding risk factors was incomplete in 22 AdmL patients, although all had at least one. When data were complete, AdmL patients tended to be more advanced regarding ASA class, cT4 and MRF+. Compared with the experimental arm of RAPIDO, tumours were more advanced in both LARCT-US and AdmL (Appendix p 3).

**Table 1:** Characteristics of rectal cancer patients in Sweden treated with total neoadjuvant treatment according to the LARCT-US concept.

	Prospective LARCT-US	Register AdmL
**Total**	273	189
Age, median (range)	63 (28–79)	65 (24–81)
Gender, male	162 (60)	111 (59)
**ECOG performance status**		
0	200 (73)	NA
1	71 (26)	NA
Not known	2 (1)	
**ASA class**		
1	43 (16)	15 (8)
2	141 (51)	103 (55)
3+	38 (14)	44 (23)
Not known	51 (19)	27 (14)
**Clinical T- and N-status**		
cT3N0	4 (1)	9 (5)
cT2-3N+	124 (45)	67 (36)
cT4N0	12 (4)	17 (9)
cT4N+	133 (49)	96 (51)
**Risk factors**		
cT4	145 (53)	113 (59)
cN2	176 (64)	95 (50)
MRF+	196 (72)	155 (82)
EMVI+	165 (60)	83 (44)
LN+	64 (23)	42 (22)
**Number of risk factors**		
1	50 (18)	32 (20)
2	77 (28)	41 (26)
3	71 (26)	42 (24)
4	54 (20)	41 (24)
5	21 (8)	11 (6)
**Tumour level**		
Low <5 cm	74 (27)	62 (33)
Mid 5–10 cm	93 (33)	76 (41)
High ≥10 cm	106 (39)	51 (27)

AdmL, *Ad modum* LARCT-US. Data for all risk factors (N-status, EMVI and LN) were unavailable for 22 (13%) patients in AdmL. Risk factors were at least one in 12 patients, at least two in nine patients, and at least three in one patient. Thus, the actual presence of risk factors was greater than shown in the AdmL-group. Data were complete concerning cT4 and MRF + where higher percentages were observed in the AdmL group. Percentages are calculated based upon the 164–176 patients where all information was available.

NA, not available; ECOG, Eastern Co-operative Oncology Group; ASA, American Society of Anesthesiologists; MRF+, mesorectal fascia involvement; EMVI+, extramural vascular involvement; LN+, lateral nodes involved.

All eligible patients received 5 × 5 Gy. Of consenting patients, five (2%) did not start chemotherapy (Flowchart Fig. 1). All AdmL patients had a recorded date for starting chemotherapy in SCRCR, however, actual dosage of chemotherapy was unavailable.

**Fig. 2:**
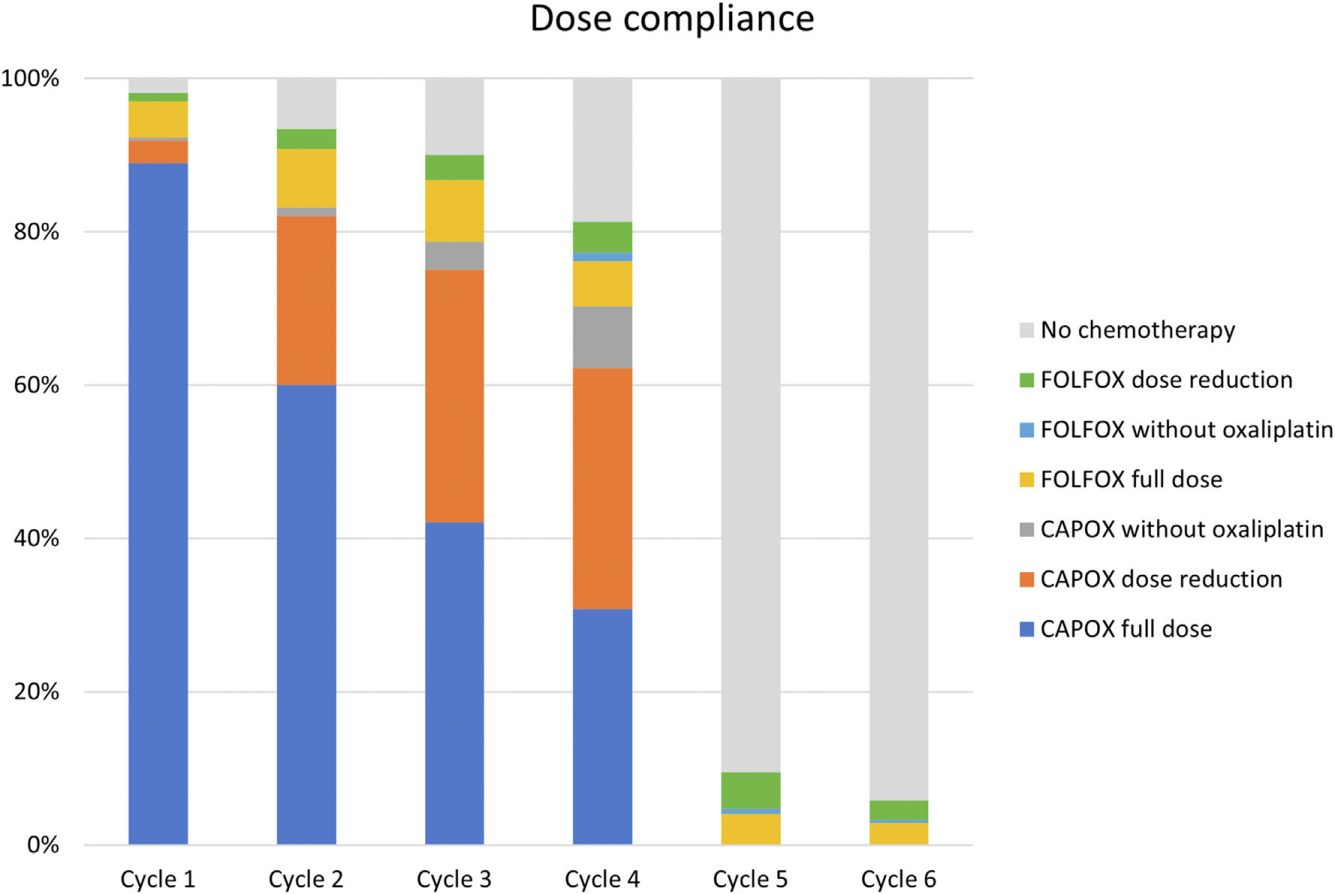
Compliance to preoperative chemotherapy in LARCT-US. Proportion of patients treated with chemotherapy per course. Four CAPOX cycles (or 6 FOLFOX) with at least 3 (5) cycles of oxaliplatin was provided to 208 (76%) patients and 239 (88%) patients received at least 75% of the number of prescribed chemotherapy cycles.

In both LARCT-US and AdmL, chemotherapy started median 21 days (IQR 16–25) from the first day of radiotherapy. In LARCT-US, 16 (6%) patients started with FOLFOX, and 20 (7%) patients changed to FOLFOX during cycle 2–4 (Figs. 1 and 2). Up-front FOLFOX was more common in AdmL (17%) and at least eight (4%) changed to FOLFOX. All but one patient received oxaliplatin during cycle 1 in LARCT-US and all but 11 (6%) in AdmL. Fig. 2 shows compliance to treatment in LARCT-US.

### Immediate outcome including surgery or watch-and-wait (W&W)

After neoadjuvant treatment, 84% and 85% of patients underwent elective surgery in LARCT-US and AdmL, respectively, and 14% and 13% entered W&W. Five and four patients, respectively, were not resected or included in W&W (Fig. 1, Table 2). The R0/R1 resection rate was 98% across groups. Fifty-two (23%) patients in LARCT-US and 61 (38%) in AdmL underwent resection of other organs/structures synchronously with TME (Table 2, Appendix p 4). In 17 (4%) patients, DM was detected after treatment initiation before or during surgery, median 112 (IQR 105–119) days.

**Table 2:** Treatment outcome in rectal cancer patients treated with total neoadjuvant treatment according to the LARCT-US concept.

	Prospective LARCT-US	Register AdmL
**Total**	273	189
**Treatment outcome**		
**No resection, M1, tox death or non-resectable**	5 (2%)	4 (2%)
**Entered W&W**	39 (14%) (100%)	23 (13%) (100%)
Regrowth within one year	8 (21%)	6 (29%)
Regrowth after one year	6 (15%)	1 (4%)
Remaining in W&W	25 (64%)	16 (67%)
**Planned resection surgery**	229 (84%) (100%)	162 (85%) (100%)
**Type of resection**		
Anterior resection	118 (51%)	69 (44%)
Abdominoperineal excision	92 (40%)	74 (45%)
Hartmann's procedure	19 (8%)	18 (11%)
**Extended surgery**	52 (23%)	61 (38%)
**Resection plane**		
Mesorectal	113 (49%)	80 (45%)
Intramesorectal	16 (7%)	9 (5%)
Muscular	2 (1%)	15 (8%)
Not known	98 (43%)	73 (41%)
**Residual tumour classification, CRM**		
R0 (>1 mm)	210 (92%)	143 (88%)
R1 (≤1 mm)	14 (6%)	16 (10%)
R2	5 (2%)	4 (2%)
**Pathologic T-stage if planned surgery**		
ypT0	40	30
ypT1	6	9
ypT2	36	28
ypT3	118	82
ypT4	28	26
**Pathologic N-stage if planned surgery**		
ypN0	135	118
ypN1	73	48
ypN2	21	9
**Complete response (CR)**	65 (24%)	44 (23%)
Pathologic complete response (pCR)	34	27
Entered W&W, no regrowth first year (s-cCR)	31	17
**NAR score**^a^		
Low	85 (32%)	67 (37%)
Intermediate	94 (35%)	72 (40%)
High	89 (33%)	40 (24%)
Median	13	8.4
**Posttreatment M-stage**		
M0	261	183
M1^b^	11 (4%)	6 (4%)
**Early locoregional failure (eLRF)**^c^	11 (4%)	6 (4%)
**Curatively treated**^d^	258 (95%)	179 (95%)

AdmL, *Ad modum* LARCT-US, W&W, watch-and-wait; CRM, circumferential resection margin; NAR, neoadjuvant rectal. Complete response (CR) is the sum of pCR in planned resected patients (resection performed without a registered period of W&W) and patients entering W&W without regrowth within a year from the start of radiotherapy (s-cCR), no distant metastases.

a Patients remaining in W&W for more than one year were assigned a low score, for all others, the actual score was used.

b One patient died during treatment and was not evaluated. In LARCT-US, distant metastasis appeared during treatment or at surgery in four non-resected patients, two R2-resected, four R0/1 resected and one entering W&W with early regrowth. In AdmL, the corresponding numbers were three non-resected, one R2-resected, and two R0/1 resected.

c Non-resected primary tumour (one patient with toxic death not included) unless the patient entered W&W or an R2-resection. Ten of these patients also had distant metastasis.

d R0/1 resected or entered W&W, no distant metastases before or at surgery.

Time between the first day of radiotherapy and surgery in non-W&W patients ranged from 64 to 273 days (median 136, IQR 126–148 in LARCT-US and 139, IQR 129–160 in AdmL). In patients receiving four cycles of CAPOX, the time within the IQRs corresponds to surgery 4–8 weeks after the last chemotherapy dose. The shortest time to surgery after four CAPOX was 113 days, or two weeks after termination of chemotherapy. In patients operated beyond 200 days, corresponding to 13 weeks after the last chemotherapy, unregistered W&W attempts may have occurred.

### Primary and secondary tumour outcomes

In LARCT-US, 34 resected patients had pCR and 31 s-cCR, and in AdmL the corresponding numbers were 27 and 17. Thus, the primary endpoint was reached in 65/273 (24%, 95% CI 20–28 and 44/189 (23%, 95% CI 17–30), respectively (Table 2). CR was associated with cT-stage, number of risk factors, CEA-level (available in LARCT-US) and tumour length (Appendix p 5 and 6).

A low NAR score was observed in 32–37% when patients with s-cCR were attributed a low score. Median scores were in the intermediate group (8.4–15, Table 2).

At data-lock, DrTF had occurred in 80 (29%, 95% CI 23–35) patients in LARCT-US (six non-resected including one toxic death, five R2-resections, and 69 recurrences), and 51 (27%, 95% CI 20–33) in AdmL (four non-resected, four R2-resections, and 43 recurrences). DrTF at three years was 28% (95% CI 23–34) and 26% (95% CI 19–31), and OS 88% (95% CI 84–92) and 89% (95% CI 85–93), respectively (Appendix p 7). Early locoregional failure (eLRF, i.e., no resection unless the patient entered W&W, or an R2-resection) occurred in 17 (4%) patients (Table 2), 10 of whom also had DM (Flowchart Fig. 1).

All patients undergoing an R0/R1 resection or entering W&W (irrespective of duration) without DM were considered curatively treated (Appendix p 8). Among curatively treated LARCT-US patients, recurrence occurred in 69/258 (26%, 95% CI 21–33) and in AdmL 43/179 (24%, 95% CI 17–30) patients (Table 3). Of recurrences, 16 (6%, 95% CI 4–10) and nine (5%, 95% CI 2–9) were LRR, and 63 (24%, 95% CI 19–30) and 40 (22%, 95% CI 15–28) DM in LARCT-US and AdmL, respectively (Table 3). Only 7/25 LRR (28%, 95% CI 12–49) were isolated. Of the 25 LRR, all occurred before three years or, in 4 cases, at the three-year follow-up with no recurrences after that. One hundred and forty patients have completed their five-year follow-up.

**Table 3:** Systemic and local recurrence risks in rectal cancer patients being either radically operated (R0/1, M0, n = 258 in LARCT-US, n = 179 in ad modum larctus, AdmL) or having entered a watch-and-wait (W&W) programme and treated with total neoadjuvant treatment according to the LARCT-US concept.

	LARCT-US total	LARCT-US local	LARCT-US systemic	AdmL total	AdmL local	AdmL systemic
**Total**	69 (26%)	16 (6%)	63 (24%)	43 (23%)	9 (5%)	40 (22%)
**Treatment**						
Entered W&W	6 (16%)	0	6 (16%)	3 (13%)	0	3 (13%)
Planned resection surgery	61 (28%)	16 (7%)	56 (25%)	40 (25%)	9 (6%)	37 (22%)
**Type of resection**^a^						
Anterior resection	35 (30%)	9 (8%)	31 (26%)	22 (31%)	3 (4%)	21 (29%)
Abdominoperineal excision	23 (27%)	7 (8%)	22 (26%)	15 (20%)	5 (7%)	14 (17%)
Hartmann's procedure	3 (18%)	0	3 (18%)	3 (17%)	1 (6%)	2 (11%)
**Resection plane**^a^						
Mesorectal	28 (25%)	3 (3%)	27 (25%)	16 (22%)	3 (4%)	15 (19%)
Intramesorectal	5 (33%)	1 (6%)	5 (33%)	5 (56%)	1 (11%)	5 (56%)
Muscular	2 (100%)	2 (100%)	1 (50%)	4 27%)	2 (13%)	4 (27%)
Not known	26 (28%)	10 (10%)	23 (25%)	15 (22%)	3 (5%)	13 (19%)
**Residual tumour classification**^a^						
R0 (>1 mm)	54 (26%)	14 (7%)	49 (24%)	32 (23%)	6 (4%)	31 (20%)
R1 (≤1 mm)	7 (50%)	2 (14%)	7 (50%)	6 (40%)	3 (20%)	6 (38%)
**Pathologic T-stage**^a^						
ypT0	1 (3%)	0	1 (3%)	0	0	0
ypT1-2	6 (!4%)	3 (7%)	4 (9%)	7 (20%)	1 (3%)	6 (17%)
ypT3	42 (36%)	10 (8%)	41 (35%)	24 (35%)	6 (9%)	22 (32%)
ypT4	12 (52%)	3 (11%)	10 (43%)	9 (34%)	2 (8%)	9 (29%)
**Pathologic N-stage**^a^						
ypN0	16 (12%)	3 (2%)	16 (12%)	15 (13%)	6 (5%)	12 (11%)
ypN1	28 (45%)	9 (15%)	24 (39%)	21 (53%)	2 (5%)	21 (53%)
ypN2	17 (68%)	4 (16%)	16 (64%)	4 (50%)	1 (13%)	4 (50%)
**Response**						
**s-cCR**	4 (13%)	0	4 (13%)	0	0	0
**pCR**	1 (3%)	0	1 (3%)	0	0	0
**Non-CR**	64 (33%)	16 (8%)	58 (30%)	43 (31%)	9 (7%)	40 (29%)
**NAR score**^b^						
Low	6 (7%)	1 (1%)	6 (7%)	3 (4%)	0	3 (4%)
Intermediate	18 (20%)	3 (3%)	17 (19%)	18 (24%)	6 (8%)	15 (19%)
High	45 (54%)	12 (14%)	40 (48%)	23 (55%)	3 (8%)	23 (55%)

AdmL, *Ad modum* LARCT-US; W&W, watch-and-wait; s-cCR, sustained clinical complete response (i.e., no regrowth within one year after the start of radiotherapy); pCR, pathologic complete response; NAR, neoadjuvant rectal.

a Relates only to patients operated primarily (planned resection surgery, n = 220 in LARCT-US, n = 156 in AdmL).

b Patients entering W&W with a duration above one year were assigned a low score. All others had the score as recorded after surgery.

No LRR occurred in patients with pCR. Among 62 patients entering W&W, regrowth rate was 34% (95% CI 22–47, Table 2). After regrowth, all patients had R0/R1 surgery with no subsequent LRR. Thus, no patient with CR has had an LRR (Table 3). Following R0 resection, LRR occurred in 20/353 patients (6%), and after R1 in 5/30 (17%). The overall LRF rate (eLRF + LRR) in the ITT population was 42/462 (9%). Patients with an LRF had more advanced tumours that required extended surgery more often than non-LRF patients (Appendix p 9–11).

At three years, DM in the ITT population was detected in 112/462 patients (24% (95% CI 20–28), of which 17 (4% (95% CI 2–6) occurred during treatment. Table 3 shows DM risk according to treatment response in curatively treated patients. A low risk was observed in good-responders (pCR 1/61 (3%), s-cCR 4/48 (8%)). In W&W patients with regrowth within one year, DM occurred in 5/14 (36%).

NAR scores also discriminated between recurrence risks (low <10%, intermediate about 20% and high about 50%, Table 3). The individual components of the NAR score, ypT, ypN, and cT-stage, also correlated with recurrence risk. No association between type of surgery and recurrence risks was observed, as opposed to resection margin and quality of the specimen.

### Toxicity to treatments

In LARCT-US, 19 (7%) patients reported radiotherapy toxicity, mostly grade 3 diarrhoea (Appendix p 12). Toxicity was associated with prolonged time between start of radiation and chemotherapy (median 29 days (IQR 18–35) vs 21 (IQR 16–23) days), and treatment failure (n = 2) or early discontinuation (n = 2).

During chemotherapy, 130 (47%) LARCT-US patients reported grade 3+ toxicity, mostly during the first two cycles, however, less than in RAPIDO (Appendix p 12). Most common toxicities were diarrhoea and non-febrile neutropenia. One toxic death occurred in a 61-year-old male who suddenly died at home after the third CAPOX cycle; no autopsy was performed because of the covid pandemic. Surgical complications are shown in Appendix p 13. No 30-day postoperative mortality occurred.

Of curatively treated, recurrence-free patients, 144 (77%) responded to the QoL questionnaires. Detailed results will be reported separately. An anticipated reduction in sensory problems following fewer oxaliplatin-containing cycles compared to RAPIDO is shown in Appendix p 14. For example, tingling and numbness in toes and feet, being most problematic, had mean scores 24 (SD31) vs 43 (SD37) and 26 (SD29) vs 35 (SD37), respectively.

## Discussion

Complete response rates (CR, sum of pCR and s-cCR) after neoadjuvant treatment are chiefly dependent upon the treatment efficacy and patient mix, given that reasonable time elapses between treatment initiation and re-assessment/surgery. The CR-rate in the ITT-population of LARCT-US/AdmL (23–24%) was substantially higher than that of CRT in the RAPIDO trial (13%) and close to the experimental TNT arm (29% in the ITT-population). This result was obtained with less chemotherapy despite more advanced tumours (e.g., cT4 in 53–59% vs 33%, Appendix p 3), emphasized by a high proportion of extended surgery (Appendix p 4). Actually, more advanced tumour stages were included in LARCT-US/AdmL than in any other TNT study, except a Polish trial (Appendix p 15–19).^9^^,^^17^ In studies with TNT, CR rates above 20% are repeatedly reported whereas after CRT, the reported CR rates are 12–15% (pCR/cCR). Higher rates are achieved in less advanced stages, although referred to as locally advanced (cT3-4, any cN+). CRT with increased radiation doses using brachytherapy may provide higher CR-rates.^18^ Chemotherapy alone does not achieve the same CR rates unless, again, earlier stages are included.^19^

The CR rate in this study is also similar to the STELLAR trial^8^ providing the same TNT (CR 22% vs 12% after CRT), and to those in TNT studies using CRT, such as the PRODIGE-23 trial (CR 26%)^7^ and the CAO/ARO/AIO-12 trial^20^ (FOLFOX before or after CRT, CR 21% and 28%, respectively) (Appendix p 15–19). LARCT-US/AdmL also included more advanced tumours than the OCUM trial^21^ (cT4 30%) providing neoadjuvant CRT only to patients with high-risk features.

Inclusion and exclusion criteria in LARCT-US/AdmL were identical to RAPIDO and the teams including patients were unchanged. However, RAPIDO was a multicentre randomised trial whereby exclusion of co-morbid patients and the most advanced tumours may have occurred, whereas all patients were treated during the inclusion period of LARCT-US. AdmL tumours were even more advanced than LARCT-US, further supporting that routinely treated patients differ from those in prospective studies/trials. Reaching the same CR rate (W&W was not an option in RAPIDO although practised in a few patients) with worse tumour and patient characteristics indicate that after scRT, four cycles of CAPOX are as effective as six cycles in reaching CR. It also appears equally effective to TNT schedules using CRT, being used in most TNT studies (see Appendix p 15–19) considering that younger patients and less advanced tumours were treated. In LARCT-US/AdmL, cT-stage and number of risk factors were, besides CEA-level and tumour length, associated with obtaining CR.

A pCR or a cCR are not identical although considerable overlap exists. If pCR is reached, the recurrence risk is limited.^22^^,^^23^ In patients entering a W&W programme, tumour regrowth occurs in about one third, mostly salvageable by radical surgery.^22^^,^^24^ In LARCT-US/AdmL, all W&W patients with regrowth underwent surgery with no subsequent LRR. No specific quality control of the cCR evaluation was made in this study, but all hospitals followed a national protocol using standard international criteria (clinicaltrials.gov NCT03125343). The rate of regrowth (34%) was similar to that reported from dedicated centres (cT3 31%, cT4 37%).^22^^,^^25^ Patients with regrowth are at increased risk of DM, as also seen here, and may indicate that further selection before entering a W&W programme is preferable.^26^

To compare outcome of the secondary endpoints LRF/LRR, DM, DFS/DrTF and OS between LARCT-US/AdmL and RAPIDO is more difficult than pCR/cCR rates, but only small differences were noted. The numerically higher eLRF rates in LARCT-US/AdmL than in RAPIDO (4% vs 2%), predominantly caused by no resection following DM during the neoadjuvant chemotherapy, reflect more advanced tumours. CAPOX/FOLFOX has limited capability to prevent progression from occult to detectable metastases. Overall, DM rates of 23% in LARCT-US/AdmL are similar to the TNT and CRT arms in RAPIDO at 20% and 27%, respectively.

Despite a higher proportion of cT4 and MRF+, the numerical risk of LRR in R0/R1 resected patients in LARCT-US/AdmL compares favourably to the TNT arm of RAPIDO (overall 6% vs 7% after median 4.8 vs 4.6 years follow-up).^6^ LRR rate of LARCT-US/AdmL will be monitored to compare with the concerning rate of 10% reported in the TNT arm of RAPIDO after median 5.6 years of follow-up.^27^ The favourable LRR rate in LARCT-US/AdmL with no LRR so far detected beyond three years, together with the STELLAR^8^ trial, contradicts scRT being inferior to CRT within a TNT schedule. It is currently unknown why the increased LRR rates in the TNT arm of RAPIDO occurred, despite being extensively investigated. It can be speculated that 6 cycles of CAPOX (or 9 cycles of FOLFOX) may be too long in non-responding patients; if this is the case, 4 cycles are less detrimental.

Intertrial comparisons of NAR-scores are difficult where the proportions of operated and non-operated differ. In this report, where patients with s-cCR were assigned a low NAR score (a reasonable assumption had they been operated), similar NAR scores to the TNT arm in RAPIDO were observed (data not shown).

Treatment toxicity could be expected to be similar in LARCT-US compared to the TNT arm in RAPIDO, except for cumulative neurotoxicity due to the reduction in oxaliplatin-containing cycles. Comparisons between studies are difficult although the CRFs and the toxicity grading were the same, but, if anything, overall and individual toxicities were slightly less in LARCT-US (Appendix p 12). Radiation-induced toxicity did, as expected, not differ and was similar to the scRT arm with delayed surgery in the Stockholm III trial,^28^ but less than reported from a prospective study with detailed toxicity reporting.^29^ Reasons for inferior chemotherapy compliance in LARCT-US compared to RAPIDO are unknown but could reflect a real-life situation and slightly older patients and with worse performance status. Postoperative morbidity was comparable to what was reported in a US retrospective rectal cancer consortium study (cT4 in 13%).^30^

The pragmatic design of LARCT-US, and the register-based data-capture in AdmL inevitably entails limitations, particularly concerning toxicity reporting. During the covid pandemic, the QoL evaluations for patients included in LARCT-US were delayed which could have resulted in less remaining neurotoxicity. However, despite some missing data, the registered characteristics of patients in LARCT-US, and in particular in AdmL were worse, reflecting a real-life situation where all patients are eligible and must be treated. This and the meticulous registration of both pCR and cCR justifies comparison with outcomes in RAPIDO. The SCRCR does not contain complete information about all risk factors (cN2, EMVI, and LN+) used to identify the patients, resulting in some missing information in AdmL. However, this together with no registration of ECOG performance status and comorbidity resulted in that it is not possible to define the group eligible for the treatment. Most probably, the included 462 (+ the 20 excluded) patients constitute the far majority of eligible patients diagnosed during the defined period in the 5/6 participating Swedish health care regions covering a population of about 8 million.

Further, it could be argued that time for s-cCR should be calculated from reassessment rather than start of radiotherapy, but the latter was chosen to facilitate comparisons between LARCT-US and AdmL. In LARCT-US, complete three-year follow-up of all patients was assured but some uncertainty existed concerning AdmL. The reported lower recurrence risk in AdmL may reflect less complete follow-up/registration in SCRCR. The results in LARCT-US are, thus, slightly more valid.

In conclusion, in LARC with high recurrence risk, an abbreviated TNT including scRT followed by four rather than six CAPOX cycles as in the RAPIDO trial appears to have the same tumour cell kill efficacy in a real-life situation including more advanced tumours than in trials. The low risk of LRF/LRR appears promising and implementation of this resource-saving schedule in routine care can be encouraged. Four cycles cannot prevent more systemic recurrences than six but may reduce LRR if local progression occurs in poor-responders.

## Contributors

BG, TK and ÅB designed the study. BG, TK, EA, ÅB, TF, CL, IL, AP, NE, KA, KB, JH, MZ, PJN and collaborative investigators provided the data. BG and PJN did the literature search. BG, TK, II and PJN had full access to all data, analysed and verified the data and designed the figures. BG, TK, EA and PJN interpreted the results. BG wrote the manuscript. BG, TK, EA, IL, ÅB, and PJN read and reviewed the manuscript. All authors approved the final version of the manuscript. The corresponding author had final responsibility to submit for publication.

## Data sharing statement

Please see Appendix p 20.

## Declaration of interests

BG reports research support from the Swedish Cancer Society and the Foundation Oncology Department in Uppsala Research Fund, EA reports research support from the Swedish Cancer Society, Swedish Research Council and Grants from the Swedish state under the agreement between the Swedish government and the county councils, the ALF-agreement. All other authors declare no competing interests.

## Appendix A Supplementary data

Supplementary material

Protocol

## References

[1] Bosset J.F., Collette L., Calais G. (2006). Chemotherapy with preoperative radiotherapy in rectal cancer. N Engl J Med.

[2] Braendengen M., Tveit K.M., Berglund Å. (2008). A randomized phase III study (LARCS) comparing preoperative radiotherapy alone versus chemoradiotherapy in non-resectable rectal cancer. J Clin Oncol.

[3] Breugom A.J., Swets M., Bosset J.F. (2015). Adjuvant chemotherapy after preoperative (chemo)radiotherapy and surgery for patients with rectal cancer: a systematic review and meta-analysis of individual patient data. Lancet Oncol.

[4] Bujko K., Glimelius B., Valentini V., Michalski W., Spalek M. (2015). Postoperative chemotherapy in patients with rectal cancer receiving preoperative radio(chemo)therapy: a meta-analysis of randomized trials comparing surgery +/- a fluoropyrimidine and surgery + a fluoropyrimidine +/- oxaliplatin. Eur J Surg Oncol.

[5] Glimelius B., Myklebust T.Å., Lundqvist K., Wibe A., Guren M.G. (2016). Two countries - two treatment strategies for rectal cancer. Radiother Oncol.

[6] Bahadoer R., Dijkstra E., van Etten B. (2021). Short-course radiotherapy followed by chemotherapy before total mesorectal excision (TME) versus preoperative chemoradiotherapy, TME, and optional adjuvant chemotherapy in locally advanced rectal cancer (RAPIDO): a randomised, open-label, phase 3 trial. Lancet Oncol.

[7] Conroy T., Bosset J.F., Etienne P.L. (2021). Neoadjuvant chemotherapy with FOLFIRINOX and preoperative chemoradiotherapy for patients with locally advanced rectal cancer (UNICANCER-PRODIGE 23): a multicentre, randomised, open-label, phase 3 trial. Lancet Oncol.

[8] Jin J., Tang Y., Hu C. (2022). Multicenter, randomized, phase III trial of short-term radiotherapy plus chemotherapy versus long-term chemoradiotherapy in locally advanced rectal cancer (STELLAR). J Clin Oncol.

[9] Cisel B., Pietrzak L., Michalski W. (2019). Long-course preoperative chemoradiation versus 5 x 5 Gy and consolidation chemotherapy for clinical T4 and fixed clinical T3 rectal cancer: long-term results of the randomized polish II study. Ann Oncol.

[10] Aschele C., Glynne-Jones R. (2023). Selecting a TNT schedule in locally advanced rectal cancer: can we predict who actually benefits?. Cancers (Basel).

[11] Grothey A., Sobrero A.F., Shields A.F. (2018). Duration of adjuvant chemotherapy for stage III colon cancer. N Engl J Med.

[12] Tang Y., Jin J., Li S. (2016). The initial results for a phase 3 study of short-term versus LongTerm chemoradiation therapy in locally advanced rectal cancer (STELLAR trial). Int J Radiat Oncol Biol Phys.

[13] RCC (2020). https://kunskapsbankencancercentrumse/diagnoser/tjock-och-andtarmscancer/vardprogram/.

[14] George T.J., Allegra C.J., Yothers G. (2015). Neoadjuvant rectal (NAR) score: a new surrogate endpoint in rectal cancer clinical trials. Curr Colorectal Cancer Rep.

[15] Dijkstra E.A., Hospers G.A.P., Kranenbarg E.M. (2022). Quality of life and late toxicity after short-course radiotherapy followed by chemotherapy or chemoradiotherapy for locally advanced rectal cancer - the RAPIDO trial. Radiother Oncol.

[16] Postma T.J., Aaronson N.K., Heimans J.J. (2005). The development of an EORTC quality of life questionnaire to assess chemotherapy-induced peripheral neuropathy: the QLQ-CIPN20. Eur J Cancer.

[17] Bujko K., Wyrwicz L., Rutkowski A. (2016). Long-course oxaliplatin-based preoperative chemoradiation versus 5 x 5 Gy and consolidation chemotherapy for cT4 or fixed cT3 rectal cancer: results of a randomized phase III study. Ann Oncol.

[18] Appelt A.L., Ploen J., Harling H. (2015). High-dose chemoradiotherapy and watchful waiting for distal rectal cancer: a prospective observational study. Lancet Oncol.

[19] Schrag D., Shi Q., Weiser M.R. (2023). Preoperative treatment of locally advanced rectal cancer. N Engl J Med.

[20] Fokas E., Allgauer M., Polat B. (2019). Randomized phase II trial of chemoradiotherapy plus induction or consolidation chemotherapy as total neoadjuvant therapy for locally advanced rectal cancer: CAO/ARO/AIO-12. J Clin Oncol.

[21] Ruppert R., Junginger T., Kube R. (2023). Risk-adapted neoadjuvant chemoradiotherapy in rectal cancer: final report of the OCUM study. J Clin Oncol.

[22] van der Valk M.J.M., Hilling D.E., Bastiaannet E. (2018). Long-term outcomes of clinical complete responders after neoadjuvant treatment for rectal cancer in the International Watch & Wait Database (IWWD): an international multicentre registry study. Lancet.

[23] Socha J., Kepka L., Michalski W., Paciorek K., Bujko K. (2020). The risk of distant metastases in rectal cancer managed by a watch-and-wait strategy - a systematic review and meta-analysis. Radiother Oncol.

[24] Hall W.A., Smith J.J. (2023). Achieving a cure without total mesorectal excision in rectal adenocarcinoma. J Clin Oncol.

[25] Chadi S.A., Malcomson L., Ensor J. (2018). Factors affecting local regrowth after watch and wait for patients with a clinical complete response following chemoradiotherapy in rectal cancer (InterCoRe consortium): an individual participant data meta-analysis. Lancet Gastroenterol Hepatol.

[26] Fernandez L.M., Sao Juliao G.P., Renehan A.G. (2023). The risk of distant metastases in patients with clinical complete response managed by watch and wait after neoadjuvant therapy for rectal cancer: the influence of local regrowth in the international watch and wait database. Dis Colon Rectum.

[27] Dijkstra E.A., Nilsson P.J., Hospers G.A.P. (2023). Locoregional failure during and after short-course radiotherapy followed by chemotherapy and surgery compared to long-course chemoradiotherapy and surgery - a five-year follow-up of the RAPIDO trial. Ann Surg.

[28] Pettersson D., Cedermark B., Holm T. (2010). Interim analysis of the Stockholm III trial of preoperative radiotherapy regimens for rectal cancer. Br J Surg.

[29] Verweij M.E., Hoendervangers S., von Hebel C.M. (2023). Patient- and physician-reported radiation-induced toxicity of short-course radiotherapy with a prolonged interval to surgery for rectal cancer. Colorectal Dis.

[30] Bauer P.S., Gamboa A.C., Otegbeye E.E. (2023). Short-course radiation with consolidation chemotherapy does not increase operative morbidity compared to long-course chemoradiation: a retrospective study of the US rectal cancer consortium. J Surg Oncol.

