# Supplementary material for: Total neoadjuvant treatment using short-course radiotherapy and four CAPOX cycles in locally advanced rectal cancer with high-risk criteria for recurrence: a Swedish nationwide cohort study (LARCT-US)
Source: eClinicalMedicine. 2024 Aug 5;75:102771. doi: 10.1016/j.eclinm.2024.102771 (PMC11577565; doi:10.1016/j.eclinm.2024.102771)
Supplement: Supplementary material [file mmc1.docx]

**ONLINE SUPPLEMENT**

**TABLE OF CONTENTS**

|  |  | Page |
| --- | --- | --- |
| Table S1 | Participating institutes and collaborative investigators | 2 |
| Table S2 | Characteristics of rectal cancer patients in Sweden treated with total neoadjuvant treatment according to the LARCT-US concept compared with the experimental arm of the RAPIDO trial | 3 |
| Table S3 | Additional surgical resections, as reported in the CRFs (LARCT-US) or SCRCR (LARCT-US and AdmL) and of those in the experimental arm of RAPIDO for comparison | 4 |
| Table S4 | Factors associated with CR (pCR or cCR with a duration exceeding 12 months from the start of radiotherapy) in rectal cancer patient treated with total neo-adjuvant therapy according to LARCT-US | 5 |
| Figure S1 | Forest plot of the associations between pretreatment characteristics and the chance to obtain a complete response (pCR+s-cCR). | 6 |
| Figure S2 | Time to disease-related treatment failure (DrTF), overall survival and time to locoregional failure in the ITT populations and disease-free survival (DFS), and time to distant metastasis and locoregional recurrence in the recurrence population in LARCT-US and AdmL | 7 |
| Table S5 | Characteristics of rectal cancer patients curatively treated with total neoadjuvant treatment according to the LARCT-US concept | 8 |
| Table S6 | Characteristics of all rectal cancer patients treated according to the LARCT-US concept and of those with a locoregional failure (LRF), either an early locoregional failure (eLRF) or a locoregional recurrence (LRR) | 9 |
| Table S7 | Treatment and treatment outcome in radically treated rectal cancer patients according to the LARCT-US concept and of those with a locoregional recurrence (LRR) | 10 |
| Table S8 | Characteristics of patients with an early locoregional failure (eLRF) treated according to the LARCT-US concept (n=17, 4% of all 462 eligible patients) | 11 |
| Table S9 | Grade 3+ toxicity to the preoperative therapy in LARCT-US patients compared with the experimental arm of the RAPIDO trial | 12 |
| Table S10 | Surgical complications within 30 days after surgery and graded according to the Clavien-Dindo classification | 13 |
| Table S11 | QLQ-CIPN20 sensory scales in LARCT-US and for comparison in RAPIDO approximately three years after surgery | 14 |
| Table S12 | Clinical stages and complete remission rates in studies delivering total neo-adjuvant therapy (TNT) in patients with locally advanced rectal cancer | 15-16 |
|  | References to supplementary material including Table S12 | 17-19 |
| Table S13 | Data sharing statement | 20 |
|  |  |  |

**Table S1. Participating institutes and collaborative investigators**

| Location | Institute | | Investigator | Contributed patients to |
| --- | --- | --- | --- | --- |
| Borås | Södra Älvsborgs sjukhus | | Johan Haux | AdmL |
| Falun | | Falu Lasarett | Åke Berglund | LARCT-US |
| Gävle | | Gävle Sjukhus | Marie Zajicova | LARCT-US+AdmL |
| Göteborg | | Sahlgrenska Universitetssjukhuset | Eva Angenete | LARCT-US |
| Jönköping | | Länssjukhuset Ryhov | Karin Adolfsson | LARCT-US+AdmL |
| Kalmar | | Kalmar Hospital | Charlotte Bratthäll | LARCT-US |
| Karlstad | | Centralsjukhuset i Karlstad | Kristina Bonde | LARCT-US+AdmL |
| Linköping | | Linköpings Universitet | Nils Elander | AdmL |
| Mora | | Mora Lasarett | Åke Berglund | LARCT-US |
| Skövde | | Skaraborgs Sjukhus Skövde | Johan Haux | LARCT-US+AdmL |
| Stockholm | | Karolinska Universitetssjukhuset, Solna | Tone Fokstuen  Per J Nilsson | LARCT-US+AdmL |
| Stockholm | | Södersjukhuset | Cecilia Lagerbäck | LARCT-US |
| Stockholm | | St Görans sjukkhus | Caroline Staff | LARCT-US+AdmL |
| Sundsvall | | Sundsvalls Sjukhus | Maria Hansen | LARCT-US+AdmL |
| Umeå | | Norrlands Universitetssjukhus | Ingrid Ljuslinder | LARCT-US |
| Uppsala | | Akademiska Sjukhuset | Bengt Glimelius  Tanweera Kahn | LARCT-US+AdmL |
| Västerås | | Västmanlands Sjukhus | Andrzej Piwowar | LARCT-US+AdmL |
| Örebro | | Universitetssjukhuset | Karin Nyström | LARCT-US |

Patient recruitment to the LARCT-US study started at three hospitals (Uppsala, Falun, and Mora). It was then estimated that about 60 patients could be treated for three years. Many other hospitals started to treat their patients similarly and most of them gradually joined the prospective study after ethical approval. Two hospitals (Borås, Linköping) declared upfront that they would treat their patients as in the observational study but not seek informed consent and not complete the CRFs. Ethical approval was obtained to retrospectively evaluate all patients in Sweden with LARC and high-risk criteria treated with the LARCT-US schedule (ad modum LARCT-US or AdmL) and registered in the national quality register Swedish Colorectal Cancer Register (SCRCR with almost 100% coverage) between August 2016 until the results of the RAPIDO trial were released. When this retrospective evaluation was approved, two hospitals (Karlstad, Västerås) stopped study inclusion but continued to treat their patients AdmL. During the covid pandemic, all but three hospitals (Umeå, Mora and Örebro) stopped inclusion in any study but continued to treat their patients as before, i.e., AdmL. The totally 482 identified patients constitute all patients thus treated in Sweden during this almost four-year period. Since the patient cohort constitutes all patients in a defined large population during a defined time period, no precise estimates of the number of patients or power calculations were done. Based upon the incidence of rectal cancer in Sweden and the proportion being eligible, i.e., having any of the risk criteria ^1^, between 100-150 patients could be treated per year in these health care regions. No patient was identified from one out of six health care regions who declared that they continued to treat their patients with conventional chemoradiotherapy until the RAPIDO results were known.

**Table S2. Characteristics of rectal cancer patients in Sweden treated with total neoadjuvant treatment according to the LARCT-US concept compared with the experimental arm of the RAPIDO trial**

|  | **Prospective**  **LARCT-US** | **Register**  **AdmL** | **Rapido**  **experimental arm** |
| --- | --- | --- | --- |
| **Total** | 273 | 189 | 460 |
| Age, median (range) | 63 (28-78) | 65 (24-81) | 61 (31-83) |
| Gender, male | 162 (60) | 111 (59) | 299 (65) |
| **ECOG performance status** |  |  |  |
| 0 | 200 (73) | NA | 368 (80) |
| 1  Not known | 71 (26)  2 (1) | NA | 92 (20) |
| **Clinical T- and N-status** |  |  |  |
| cT3N0 | 4 (1) | 9 (5) | 24 (5) |
| cT2-3N+ | 126 (46) | 67 (36) | 289 (63) |
| cT4N0 | 12 (4) | 17 (9) | 23 (5) |
| cT4N+ | 133 (49) | 96 (51) | 124 (27) |
| **Risk factors** |  |  |  |
| cT4 | 145 (53) | 113 (59) | 151 (33) |
| cN2 | 176 (64) | 95 (50) | 312 (68) |
| MRF+ | 196 (72) | 155 (82) | 284 (62) |
| EMVI+ | 165 (60) | 83 (44) | 147 (32) |
| LN+ | 64 (23) | 42 (22) | 63 (14) |
| **Number of risk factors** |  |  |  |
| 1 | 50 (18) | 32 (20) | 158 (34) |
| 2 | 77 (28) | 41 (26) | 160 (35) |
| 3 | 71 (26) | 42 (24) | 98 (21) |
| 4 | 54 (20) | 41 (24) | 39 (8) |
| 5 | 21 (8) | 11 (6) | 7 (2) |
| **Tumour level** |  |  |  |
| Low <5 | 74 (27) | 62 (33) | 103 (22) |
| Mid 5-10 | 93 (33) | 76 (41) | 180 (39) |
| High ≥10 | 106 (39) | 51 (27) | 145 (32) |
| Not known |  |  | 32 (7) |

*Information about all risk factors (N-status, EMVI (extramural vascular invasion) and LN (lateral node involved, designated ELLN in the RAPIDO trial) was not available for 22 (13%) patients in the AdmL group. There was at least one in 12 patients, at least two in nine patients, and at least three in one patient. Thus, in reality more risk factors were present than shown in the AdmL-group. In the aspects where complete information was present in AdmL (cT4 and MRF+), higher percentages were seen in the AdmL group. Percentages are calculated based upon the 164-176 patients where all information was available.

NA = not available, ECOG =Eastern Cooperative Oncology Group

The numbers for the RAPIDO-trial taken from Bahadoer et al Lancet Oncol 2021.^2^

**Table S3. Additional surgical resections, as reported in the CRFs (LARCT-US) or SCRCR (LARCT-US and AdmL) and in the experimental arm of RAPIDO for comparison**

|  | LARCT-US (n=229) | AdmL (n=162) | RAPIDO (n=426) |
| --- | --- | --- | --- |
| **Number of additional organs/structures resected** |  |  |  |
| None | 175 (76) | 100 (62) | 393 (92) |
| 1 organ/structure | 35 (15) | 33 (20) | 16 (4) |
| 2 organ/structures | 14 (6) | 16 (10) | 15 (4) |
| 3 organ/structures | 4 (2) | 9 (6) | 2 (1) |
| 4+ organ/structures | 3 (1) | 5 (3) | -- |
| **Resected organ/structure (or part of)** | (**n=86)** | (**n=101)** | **(n=56)** |
| Ovarium/uterus | 18 (21) | 20 (20) | 20 (39) |
| Vagina | 14 (16) | 8 (8) | 4 (8) |
| Vesiculae seminales/prostate/funiculus spermaticus | 13 (16) | 13 (13) | 11(21) |
| Urethra/bladder | 5 (6) | 14 (14) | 5 (10) |
| Colon/appendix | 7 (8) | 10 (10) | 2 (4) |
| Short bowel | 4 (5) | 5 (5) | 2 (4) |
| Spleen | -- | 1 (1) | 1 (2) |
| Liver | 1 (1) | 1 (1) | 2 (4) |
| Lateral lymph nodes | 12 (14) | 15 (15) | 2 (4) |
| Sacrum/coccyx | 2 (2) | 5 (5) | 1 (2) |
| Levator/endopelvic fascia | 7 (8) | 6 (6) | 1 (2) |
| Vertebral wall | -- | 1 (1) | 1 (2) |
| Blood vessels | 1 (1) | -- | -- |
| Abdominal wall | 1 (1) | 2 (2) | -- |

The numbers for the RAPIDO trial were taken from van der Valk et al, Radiother Oncol 2020.^3^

**Table S4. Factors associated with complete response (CR, pCR or cCR with a duration exceeding 12 months from the start of radiotherapy) in rectal cancer patients treated with total neo-adjuvant therapy according to LARCT-US**

|  | **Prospective**  **LARCT-US** | **Register**  **AdmL** | **LARCT-US + AdmL** |
| --- | --- | --- | --- |
|  | **Numbers (%)** | **Numbers (%)** | **Numbers (%, 95% CI)** |
| **Total** | 65/273 (24%) | 44/189 (23%) | 109/462 (24%,20-28) |
| Age 28-69 | 50/200 (25%) | 31/126 (25%) | 81/326 (25%, 20-30) |
| 70+ | 15/73 (21%) | 13/63 (21%) | 28/136 (21%, 14-28) |
| Gender, male | 39/162 (24%) | 24/111 (22%) | 63/273 (23%, 18-29) |
| female | 26/111 (23%) | 20/78 (26%) | 46/189 (24%, 18-31) |
| **ECOG performance status** |  |  |  |
| 0 | 50/200 (25%, 19-32) | NA |  |
| 1 | 15/71 (21%, 12-32) | NA |  |
| **Clinical T- and N-status** |  |  |  |
| cT2N+ | 4/8 (42%) | 2/4 (50%) | 6/12 (50%, 21-79) |
| cT3N0 | 1/4 (25%) | 2/9 (22%) | 3/13 (23%, 5-54) |
| cT3N+ | 31/118 (26%) | 20/63 (32%) | 51/181(28%, 22-35) |
| cT4N0 | 5/12 (42%) | 4/17 (24%) | 9/29 (31%, 15-51) |
| cT4N+ | 23/131 (18%) | 18/105 (17%) | 41/236 (17%, 13-23) |
| **Risk factors*** |  |  |  |
| cT4 | 28/143 (20%) | 22/122 (18%) | 50/265 (19%,, 14-24) |
| cN2 | 37/176 (21%) | 16/95 (16%) | 53/271(20%, 15-25) |
| MRF+ | 48/197 (24%) | 35/165 (21%) | 83/362 (23%, 19-28) |
| EMVI+ | 30/164 (18%) | 10/92 (11%) | 40/256 (16%, 11-21) |
| LN+ | 17/64 (27%) | 9/45 (20%) | 26/109 (24%, 16-33) |
| **Number of risk factors** |  |  |  |
| 1 | 17/50 (34%) | 14/32 (44%) | 31/82 (38%, 27-49) |
| 2 | 17/77 (22%) | 9/41 (21%) | 26/118 (22%, 15-31) |
| 3 | 20/71 (28%) | 9/42 (21%) | 29/113 (26%, 18-35) |
| 4-5 | 11/75 (15%) | 5/52 (10%) | 16/127 (13%, 7-20) |
| Missing information | - | 7/22 (32%) | 7/32 (32%, 9-40) |
| **Tumour level** |  |  |  |
| Low <5 cm | 18/74 (24%) | 19/66 (29%) | 37/140 (26%, 19-35) |
| Mid 5-10 cm | 27/93 (29%) | 16/85 (19%) | 43/178 (24%, 18-31) |
| High ≥10 cm | 20/106 (19%) | 12/56 (21%) | 32/162 (20%, 14-27) |
| **Pretreatment CEA** |  |  |  |
| ≤ 3.6 µg/L | 40/122 (33%, 25-42) | NA |  |
| >3.6 µg/L | 24/143 (17%, 11-24) | NA |  |
| Missing information | 1/8 |  |  |
| **Tumour length (mm)** |  |  |  |
| ≤ 40 mms | 22/69 (32%) | 10/32 (31%) | 32/101 (32%, 23-42) |
| 41-69 mm | 37/146 (25%) | 11/71 (15%) | 48/217 (22%, 17-28) |
| 70+ mm | 7/58 (12%) | 5/39 (13%) | 12/97 (12%, 7-21) |
| Missing information | - | 18/47 (38%) | 18/47 (38%, 25-34) |

ECOG = Eastern Co-operative Oncology Group, MRF+ = mesorectal fascia involvement, EMVI = extramural vascular invasion, LN+ = lateral node involvement, CEA = carcinoembryonic antigen

**Figure S1**

**
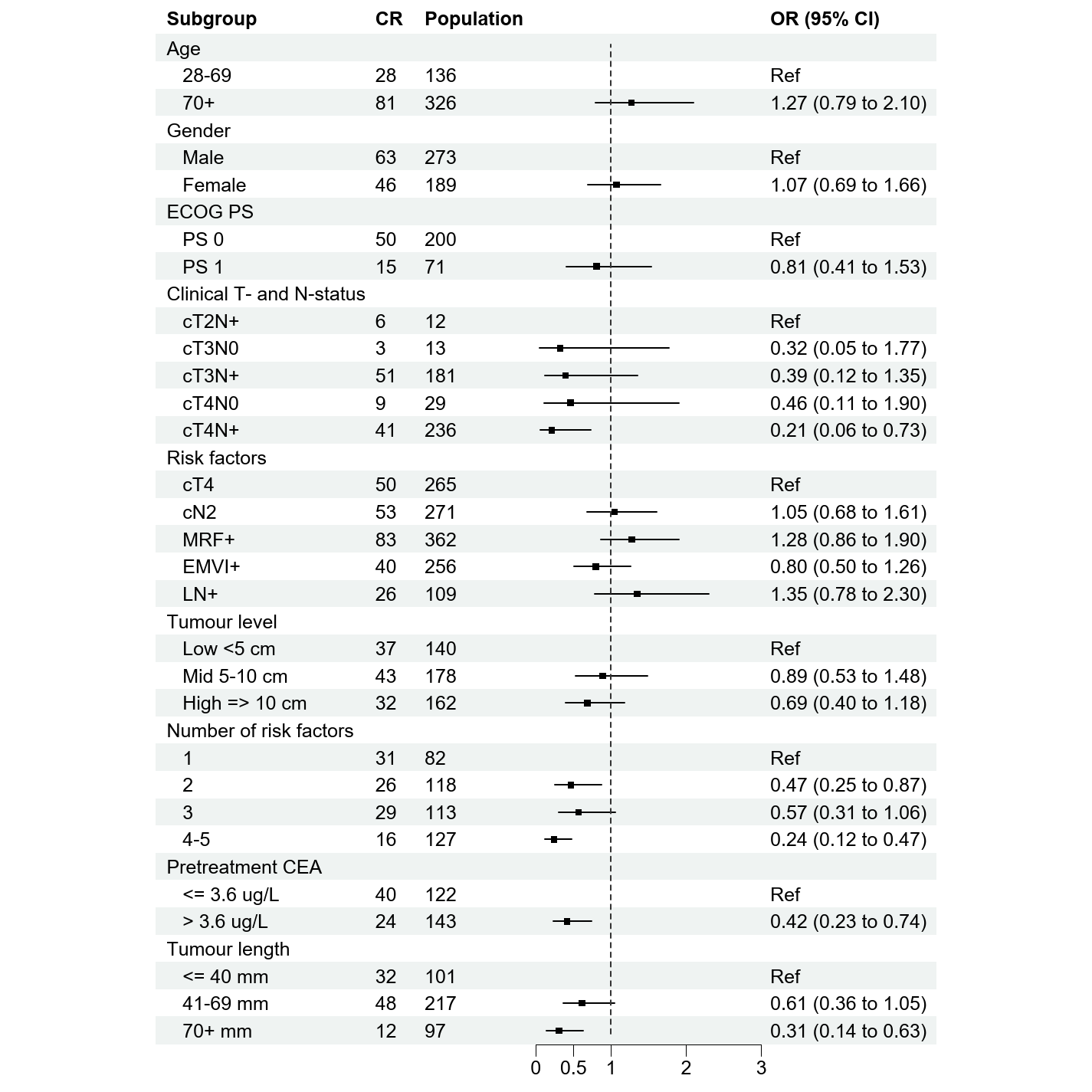
**

**Supplementary figure 1** Forest plot of the associations between pretreatment characteristics and the chance to obtain a complete response (pCR+s-cCR). Both LARCT-US and AdmL patients are included, however, Eastern Co-operative Oncology group performance status (ECOG PS) and pretreatment carcinoembryonic antigen (CEA) only available in LARCT-US.

Supplementary figure 2. Time to disease-related treatment failure (DrTF), overall survival and time to locoregional failure in the intention-to-treat (ITT) populations and disease-free survival (DFS), and time to distant metastasis and locoregional recurrence in the recurrence population in LARCT-US and AdmL. No comparisons between the cohorts were planned.

**Table S5. Characteristics of rectal cancer patients curatively treated with total neoadjuvant treatment according to the LARCT-US concept**

|  | **Prospective**  **LARCT-US** | **Register**  **AdmL** |
| --- | --- | --- |
| **Total** | 258 | 179 |
| Age, median (range) | 62 (28-79) | 66 (24-81) |
| Gender, male | 150 (58) | 106 (59) |
| **ECOG performance status** |  |  |
| 0 | 190 (74) | NA |
| 1 | 66 (26) | NA |
| **ASA class** |  |  |
| 1 | 43 (17) | 15 (8) |
| 2 | 136 (53) | 99 (56) |
| 3+ | 34 (14) | 43 (24) |
| not known | 45 (17) | 22 (12) |
| **Clinical T- and N-status** |  |  |
| cT2N+ | 8 (3) | 4 (2) |
| cT3N0 | 4 (2) | 9 (4) |
| cT3N+ | 115 (44) | 59 (33) |
| cT4N0 | 12 (5) | 16 (8) |
| cT4N+ | 119 (46) | 91 (52) |
| **Risk factors** |  |  |
| cT4 | 131 (51) | 107 (60) |
| cN2 | 165 (63) | 91 (48) |
| MRF+ | 186 (68) | 148 (82) |
| EMVI+ | 153 (59) | 75 (42) |
| ELLN+ | 59 (23) | 37 (21) |
| **Number of risk factors*** |  |  |
| 1 | 50 (19) | 32 (20) |
| 2 | 73 (28) | 39 (25) |
| 3 | 66 (26) | 42 (27) |
| 4 | 51 (20) | 34 (22) |
| 5 | 18 (7) | 10 (6) |
| **Tumour level** |  |  |
| Low <5 cm | 69 (27) | 58 (32) |
| Mid 5-10 cm | 89 (34) | 70 (40) |
| High ≥10 cm | 100 (39) | 51 (28) |

Curatively treated: R0/1 resection or entered W&W, M0

*In 22 (13%) AdmL patients, knowledge of all risk factors (cN2, EMVI and LN) was not present. All had at least 1, 12 had 1+, 9 2+ and 1 3+ risk factors. The percentages relate to the patients with complete information. For abbreviations, see supplementary table S2.

**Table S6. Characteristics of all rectal cancer patients treated according to the LARCT-US concept and of those with locoregional failure (LRF), either an early locoregional failure (eLRF) or a locoregional recurrence (LRR)**

|  | **All patients** | **Patients with LRR** | **Patients with eLRF** |
| --- | --- | --- | --- |
| **Total** | 462 | 25 (5) (100) | 17 (4) (100) |
| Age, median (range) | 64 (24-81) | 57 (38-77) | 66 (41-74) |
| Gender, male | 273 (59) | 11 (48) | 12 (71) |
| **ASA class** |  |  |  |
| 1 | 58 (12) | 4 (17) | 0 |
| 2 | 244 (53) | 13 (57) | 6 (35) |
| 3+ | 82 (17) | 4 (17) | 3 (18) |
| not known | 78 (17) | 2 (9) | 8 (48) |
| **Clinical T- and N-status** |  |  |  |
| cT3N0 | 14 (3) | 0 | 0 |
| cT2-3N+ | 192 (41) | 10 (43) | 4 (24) |
| cT4N0 | 30 (6) | 1 (4) | 1 (6) |
| cT4N+ | 227 (49) | 12 (52) | 12 (71) |
| **Risk factors*** |  |  |  |
| cT4 | 258 (56) | 13 (43) | 13 (76) |
| cN2 | 271 (59) | 16 (70) | 13 (76) |
| MRF+ | 351 (76) | 11 (48) | 16 (94) |
| EMVI+ | 248 (54) | 12 (52) | 14 (82) |
| LN+ | 106 (23) | 6 (26) | 10 (59) |
| **Number of risk factors*** |  |  |  |
| 1 | 82 (18) | 8 (35) | 0 |
| 2 | 118 (26) | 4 (17) | 3 (18) |
| 3 | 113 (24) | 4 (17) | 3 (18) |
| 4 | 95 (21) | 5 (22) | 7 (41) |
| 5 | 32 (7) | 2 (9) | 4 (24) |
| **Tumour level** |  |  |  |
| Low <5 cm | 136 (29) | 10 (43) | 7 (41) |
| Mid 5-10 cm | 169 (37) | 5 (22) | 5 (29) |
| High ≥10 cm | 157 (34) | 7 (30) | 5 (29) |
|  |  |  |  |

Figures are number of patients (per cent) unless otherwise indicated

*Not completely known for 22 AdmL patients concerning cN2, EMVI+ and LN+. For abbreviations, see Table S2

**Table S7. Treatment and treatment outcome in radically treated rectal cancer patients according to the LARCT-US concept and for those with a locoregional recurrence (LRR)**

|  | **Prospective**  **LARCT-US** | **Register**  **AdmL** | **LRR (both groups)** |
| --- | --- | --- | --- |
| **Total** | 258 | 179 | 25 |
| **Received radiotherapy** | 258 (100) | 179 (100) | 25 (100) |
| **Received chemotherapy** |  |  |  |
| None | 5 (2) | 0 | 2 (8) |
| Non-compliant | 26 (10) | NR | 5/16 (31) |
| Compliant | 228 (88) | NR | 11/16 (69) |
| Started with CAPOX | 239 (94) | 139 (78) | 17 (68) |
| Started with FOLFOX | 15 (6) | 29 (16) | 5 (20) |
| FU alone, no oxaliplatin | 0 | 11 (6) | 1 (4) |
|  |  |  |  |
| **Entered W&W** | 38 (14%) (100%) | 24 (12%) (100%) | 0 |
| Regrowth within one year | 7 (18%) | 5 (21%) |  |
| Regrowth after one year | 4 (15%) | 2 (8%) |  |
| Remaining in W&W | 27 (67%) | 17 (71%) |  |
| **Immediate resection surgery** | 220 (84%) (100%) | 171 (88%) (100%) | 25 (100%) |
| **Type of resection** |  |  |  |
| Anterior resection | 117 (53%) | 76 (44%) | 12 (48%) |
| Abdominoperineal excision | 86 (39%) | 75 (44%) | 12 (48%) |
| Hartmann’s procedure | 17 (8%) | 20 (12%) | 1 (4%) |
| **Resection plane** |  |  |  |
| Mesorectal | 110 (50%) | 77 (45%) | 6 (17%) |
| Intramesorectal | 15 (7%) | 9 (5%) | 2 (8%) |
| muscular | 2 (1%) | 15 (9%) | 4 (17%) |
| not known | 93 (42%) | 70 (41%) | 13 (56%) |
| **Extended surgery (beyond TME)** | 48 (22%) | 61 (36%) | 10 (40%) |
| **Residual tumour classification, crm** |  |  |  |
| R0 (> 1mm) | 206 (94%) | 153 (89%) | 20 (78%) |
| R1 (≤ 1 mm) | 14 (6%) | 18 (11%) | 5 (22%) |
| **Pathologic T-stage in immediately resected** |  |  |  |
| ypT0 | 36 (16%) | 31 (18%) | 0 |
| ypT1 | 8 (4%) | 9 (5%) | 0 |
| ypT2 | 35 (16%) | 28 (16%) | 4 (17%) |
| ypT3 | 118 (54%) | 78 (46%) | 16 (70%) |
| ypT4 | 23 (10%) | 25 (15%) | 3 (13%) |
| **Pathologic N-stage in immediately resected** |  |  |  |
| ypN0 | 132 (60%) | 116 (69%) | 8 (35%) |
| ypN1 | 63 (29%) | 48 (28%) | 10 (43%) |
| ypN2 | 25 (11%) | 7 (4%) | 5 (22%) |
|  |  |  |  |
| **Complete response** | 65 (25%) | 44 (25%) | 0 |
| Pathologic complete response (pCR) | 34 | 27 |  |
| Entered W&W, no regrowth first year (s-cCR) | 31 | 17 |  |
| **NAR score*** |  |  |  |
| Low | 83 (33%) | 70 (36%) | 1 (4%) |
| Intermediate | 91 (35%) | 77 (39%) | 8 (35%) |
| High | 84 (33%) | 48 (25%) | 14 (61%) |
| Median | 13 | 8.4 | 20 |

Patients with R0/1 resected tumours or having entered watch and wait (W&W), no distant metastases before or at surgery. Complete response (CR) is the sum of pCR in planned resected patients and patients entering W&W without regrowth within a year from the start of radiotherapy (s-cCR), no distant metastases.

*Patients remaining in W&W above one year were assigned a low score, for all others, the actual score was used.

**Table S8. Characteristics of patients with an early locoregional failure (eLRF) treated according to the LARCT-US concept (n=17, 4% of all 462 eligible patients)**

| **Reason for eLRF** |  |
| --- | --- |
| DM, no surgery | 7 |
| DM and R2 resection | 3 |
| R2 resection only | 6 |
| Non-resectable primary | 1 |
| **Male gender** | 12 (71%) |
| **Median age (range)** | 66 years (48-74) |
| **High risk criteria** |  |
| cT4 | 13 (76%) |
| cN2 | 13 (76%) |
| EMVI+ | 16 (94%) |
| MRF+ | 14 (82%) |
| LN+ | 10 (69%) |
| **Tumour level** |  |
| Low | 7 (41%) |
| Intermediate | 6 (35%) |
| High | 4 (24%) |
| **Compliance*** |  |
| ≥75% of prescribed |  |
| chemotherapy 6 (9) cycles | 6/9 (67%) |
| **Distant metastases** |  |
| Before/synchronously with surgery | 10 (5%) |
| After | 5 (29%) |
| No | 2 (12%) |
| **Extended surgery** (in R2-resected) | 8/9 (80%) |

One patient not operated on due to sudden death after three cycles of chemotherapy and one patient whose tumour was not resected due to distant metastases and a complete clinical response of the primary tumour with no regrowth before the patient died from his metastases are not included.

* All patients had the planned radiotherapy. Compliance only known in LARCT-US (n=9) patients. One patient was not fit for any chemotherapy, and two did not receive at least 75% of the number of planned cycles. The surgery in R2-resected patients was performed after 108 – 196 days after the first radiation fraction. The surgical attempt when the tumour was found non-resectable was performed after 166 days.

For abbreviations of risk factors, see Table S2

**Table S9. Grade 3+ toxicity in LARCT-US patients compared with the experimental arm of the RAPIDO trial**

| **Highest grade reported by patient** | **During or after radiotherapy (n=273)** | **During chemotherapy (n=268)** | **LARCT-US (n=273)** | **RAPIDO (n=460)** |
| --- | --- | --- | --- | --- |
| 3 | 19 (7%) | 126 (46%) | 129 (47%) | 191 (41%) |
| 4 |  | 3 (1%) | 3 (1%) | 30 (7%) |
| 5 |  | 1 (<1%) | 1 (<1%) | 1 (<1%) |
| *Adverse event* |  |  |  |  |
| **General** |  |  |  |  |
| Febrile neutropenia |  | 4 | 4 (1%) | 5 (1%) |
| Neutropenia (w/o fever)/thrombocytopenia |  | 12 (4%) | 12 (4%) | NR |
| Mucositis |  | 1 | 1 (<1%) | 3 (<1%) |
| Fatigue |  | 5 | 5 (2%) | 14 (3%) |
| Hand-foot syndrome |  | 3 | 3 (1%) | 8 (2%) |
| Neurological |  | 4 | 4 (1%) | 20 (4%) |
| Infection |  | 3 | 2 (<1%) | 18 (4%) |
| Cardiac |  | 4 | 4 (1%) | 7 (2%) |
| Vascular |  | 3 | 3 (1%) | 39 (8%) |
| **Gastro-intestinal** |  |  |  |  |
| Diarrhoea | 15 (5%) | 30 (11%) | 30 (11%) | 81 (18%) |
| Nausea, vomiting |  | 9 (3%) | 9 (3%) | 19 (´4%) |
| Proctitis, rectal bleeding | 2 | 6 | 6 (2%) | 8 (2%) |
| Abdominal pain |  | 4 | 4 (1%) | 15 (3%) |
| Obstipation |  | 4 | 4 (1%) | 15 (3%) |
| **Other** | 5 | 14 | 15 (5%) | 20 (4%) |

Toxicity according to CTCAE version 4.0.
Other: Impaired general condition =1, laryngospasm =6, electrolyte disturbance =2, allergic reaction =2, impaired liver function =1, oedema =1, reduced appetite =1.

The numbers for the RAPDIO trial were taken from van der Valk et al Radiother Oncol 2020.^3^

**Table S10. Surgical complications within 30 days of surgery and graded according to the Clavien-Dindo classification**

| **Complication** | **LARCT-US (n=229)** | **AdmL (n=177)** | **RAPIDO exp (n=426)** |
| --- | --- | --- | --- |
| Any complication | 92 (40%) | 68 (38%) | 215 (50%) |
| General ≥ CD II |  |  |  |
| Cardiovascular | 3 (1%) | 3 (2%) | 13 (3%) |
| Neurological | 1 (<1%) | 2 (1%) | 8 (“%) |
| Urological | 3 (1%) | 2 (1%) | 10 (2%) |
| Pneumonia | 1 (<1%) | 2 (1%) | 23 (5%) |
| Sepsis | 2 (1%) | 1 (<1%) | 14 (3%) |
| Other infection | 21 (%) | 8 (7%) | 29 (7%) |
| Other | 5 (2%) | 11 (6%) | 6 (1%) |
| Any surgical ≥ CD III | 33 (14%) | 32 (18%) | 63 (15%) |
| Intra-abdominal infection | 9 (4%) | 10 (4%) | 21 (5%) |
| Wound complication | 4 (2%) | 4 (2%) | 13 (3%) |
| Subileus/ileus | 2 (1%) | 1 (<1%) | 17 (4%) |
| Anastomotic leakage* | 14 (12%) | 9 (15%) | 14 (3%) |
| Stoma-related**†** | 3 (2%) | 3 (2%) | 3 (1%) |
| Other | 12 (5%) | 5 (3%) | 11 (3%) |
| Readmission | 30 (13%) | 27 (16%) | 58 (14%) |
| Reoperation | 18 (8%) | 22 (13%) | 42 (10%) |
| Intraabdominal infection | 5 (2%) | 9 (5%) | 13 (3%) |
| Anastomotic leakage | 7 (3%) | 7 (4%) | 10 (2%) |
| Stoma complications | 2 (1%) | 3 (1%) | 3 (<1%) |
| Bleeding | 3 (1%) | 0 | 2 (<1%) |
| Ileus | 0 | 0 | 9 (2%) |
| Other | 8 (3%) | 7 (4%) | 3 (<1%) |
| Postoperative mortality | 0 | 0 | 3 (<1%) |

Patients operated primarily. The information of toxicity was taken from the SCRCR in both groups and in addition from the CRFs in LARCT-US.

*In patients with a primary anastomosis and **†**in patients with a stoma

The numbers for the RAPDIO trial were taken from van der Valk et al Radiother Oncol 2020.^3^

**Table S11. QLQ-CIPN20 sensory scales in LARCT-US and for comparison in RAPIDO approximately three years after surgery**

| **Scale** | **LARCT-US** |  | **RAPIDO exp arm** |  |
| --- | --- | --- | --- | --- |
|  | **number** | **Mean (SD)** | **number** | **Mean (SD)** |
| **Sensory scale** |  | 14.4 (15.1) |  | 20.1 (18.9) |
| Tingling fingers or hands | 143 | 16.2 (23.6) | 238 | 21.3 (28.0) |
| Tingling toes or feet | 143 | 24.1 (30.9) | 238 | 42.7 (37.3) |
| Numbness fingers or hands | 143 | 17.7 (24.2) | 238 | 16.9 (25.4) |
| Numbness toes or feet | 143 | 26.1 (29.1) | 238 | 34.5 (37.4) |
| Pain fingers or hands | 143 | 6.9 (18.0) | 238 | 6.9 (19.0) |
| Pain toes or feet | 143 | 10.9 (22.1) | 236 | 20.2 (33.4) |
| Trouble standing or walking | 143 | 13.6 (24.1) | 236 | 19.8 (30.7) |
| Trouble distinguishing temperature | 143 | 2.8 (11.4) | 238 | 5.5 (17.1) |
| Trouble hearing | 143 | 10.9 (21.0) | 237 | 13.1 (24.2) |

Due to the Covid pandemic, many patients were not invited to respond to the questionnaire after 36±3 months as originally planned. For these reasons, questionnaires were accepted within up to 63 months. However, in the 92 patients responding within 36±3 months, the mean values did not differ more than one decimal from all patients.

The numbers for the RAPDIO trial were taken from Dijkstra et al, Radiother Oncol 2022.^4^

**Table S12. Clinical stages and complete remission rates in studies delivering total neo-adjuvant therapy (TNT) in patients with locally advanced rectal cancer**

| Study (ref) | Type of study | Neoadjuvant treatment | Allowed W&W | Number of patients | cT4 (%) | cN2 (%) | MRF (%) | pCR* resected | pCR* ITT | CR ITT* | Comments |
| --- | --- | --- | --- | --- | --- | --- | --- | --- | --- | --- | --- |
| RAPIDO ^2^ | Rand phase III | TNT, scRT+CAPOX x6 | No | 460 | 32% | 65% | 62% | 28% | 26% | 29% | A few patients entered W&W, were protocol violators |
|  |  | CRT |  | 446 | 30% | 66% | 60% | 14% | 13% | 14% |  |
| Stellar ^5^ | Rand phase III | TNT, scRT+CAPOXx4 | No | 302 | 16% | 35% | 56% | 17% | 13% | 22% | Non-inferiority design. Many patients did not have surgery. |
|  |  | CRT+AC |  | 297 | 13% | 34% | 56% | 12% | 9% | 12% |  |
| Polish ^6^ | Rand phase III | TNT, scRT+FOLFOX x2 | No | 261 | 63% | NR | NR | 17% | 14% | -- | The only trial with similar patients and tumours as in LARCT-US/AdmL |
|  |  | CRT |  | 254 | 64% | NR | NR | 12% | 9% | -- |  |
| PRODIGE-23 ^7^ | Rand phase III | TNT, FOLFIRINOXx6+CRT | No | 231 | 18% | 26% | 26% | 28% | 26% | -- |  |
|  |  | CRT |  | 230 | 16% | 23% | 28% | 12% | 11% | -- |  |
| PROSPECT ^8^ | Rand phase III | FOLFOX+ selective CRT | No | 597 | 0 | NR | NR | 22% | 21% | -- | CRT only if limited response, 9% received preop CRT, 2% postop CRT |
|  |  | CRT |  | 597 | 0 | NR | NR | 24% | 23% | -- |  |
| AIO-12 ^9^ | Rand phase III | TNT, induction 3 FOLFOX+CRT | No | 150 | 18% | NR | NR | 19% | 17% | 21% | Some patients entered W&W |
|  |  | TNT CRT+consolidation 3 FOLFOX |  | 156 | 12% | NR | NR | 27% | 25% | 28% |  |
| FOWARC ^10,11^ | Rand phase III | TNT FLv dGx3+CRT | No | 165 | 35% | 26% | 32% | 14% | 13% | -- | Addition of oxaliplatin increased pCR rates. Chemotherapy alone inferior pCR rate |
|  |  | TNT mFOLFOXx3+CRT with oxaliplatin |  | 165 | 34% | 28% | 36% | 28% | 25% | -- |  |
|  |  | mFOLFOX6 x4-6 alone |  | 165 | 30% | 26% | 31% | 7% | 6% | -- |  |
| GCR-3 ^12^ | Rand phase II | TNT, 4 CAPOX+ CRT | No | 56 | 13% | NR | 0 | 14% | 14% | -- | No benefit of TNT, besides better compliance |
|  |  | CRT |  | 52 | 6% | NR | 10% | 13% | 13% | -- |  |
| INOVA ^13^ | Rand phase II | TNT, 2 FOLFOX+bev+CRT | No | 46 | 0 | 20% | NR | 24% | 22% | -- | No benefit of adding bevacizumab |
|  |  | CRT with bevacizumab |  | 45 | 0 | 11% | NR | 11% | 11% | -- |  |
| WAIT ^14^ | Rand phase II | TNT, CRT+ 3 FLv dG | No | 25 | 4% | 63% | 60% | 16% | 16% | -- | No advantage of 3 FLv dG consolidation cycles |
|  |  | CRT |  | 24 | 20% | 76% | 50% | 25% | 25% | -- |  |
| KCSG CO 14-03 ^15^ | Rand phase II | TNT, CRT+ CAPOXx2 | No | 53 | 17% | NR | 26% | 14% | 11% | -- | Minor benefit of adding 2 CAPOX as consolidation |
|  |  | CRT |  | 55 | 18% | NR | 29% | 6% | 5% | -- |  |
| Marechal ^16^ | Rand phase II | TNT, 2 FOLFOX+CRT | No | 29 | 7% | NR | NR | 25% | 25% | -- | No difference, however, small study |
|  |  | CRT |  | 28 | 10% | NR | NR | 28% | 28% | -- |  |
| GEMCAD 1402 ^17^ | Rand phase II | TNT, FOLFOXx6+aflibercept + CRT | No | 115 | 18% | 69% | 59% | 25% | 25% | -- | More pCR if + aflibercept |
|  |  | TNT, FOLFOXx6 + CRT |  | 65 | 20% | 71% | 57% | 15% | 15% | -- |  |
| OPRA ^18^ | Rand phase II | TNT, induction FOLFOXx8 +CRT | Yes | 158 | 15% | NR | NR | -- | -- | 45% |  |
|  |  | TNT, CRT+consolidation FOLFOXx8 |  | 166 | 11% | NR | NR | -- | -- | 60% |  |
| GRECCAR 4 ^19,20^ | Rand phase II | Induction FOLFIRINOX, good responders, surgery, or CRT | No | 30 | 0 | 0 | 87% | 10% vs 58% | -- | -- | Good responders randomised to surgery or CRT, poor responders to CRT 50 or 60 Gy, however, no difference |
|  |  | Poor responders, CRT 50 or 60 Gy |  | 103 | 24% | 0 | 89% | 17% | -- | -- |  |
| Sclafani ^21^ | Rand Phase II x 2 | TNT, CAPOXx4 +CRT | No | 269 | 22% | 36% | 62% | 20% | 18% | -- | Randomised ±cetuximab, no difference, pooled data shown |
| Myerson ^22^ | Phase II | TNT, scRT+FOLFOXx4 | No | 76 | 9% | NR | NR | 25% |  |  |  |
| Timing consortium ^23^ | Phase II | CRT | No | 60 | 2% | 2% | NR | 18% |  |  | Sequential studies |
|  |  | TNT, CRT+FOLFOXx2 |  | 67 | 1% | 4% | NR | 25% |  |  |  |
|  |  | TNT, CRT+FOLFOXx4 |  | 67 | 4% | 13% | NR | 30% |  |  |  |
|  |  | TNT, CRT+FOLFOXx6 |  | 65 | 5% | 16% | NR | 38% |  |  |  |
| AVACROSS ^24^ | Phase II | CAPOX+bev+CRT | No | 47 | 12% | NR | 19% | 36% |  |  |  |
| PROARCT ^25^ | Phase II | TNT, FOLFOX+ split RT | No | 40 | 12% | 28% | NR | 20% | 20% | -- |  |
| Chin ^26^ | Phase II | TNT, scRT+ boost+ FOLFOXx8 | Yes | 86 | 20% | 46% | 42% | -- |  | 50% | Local regrowth 21% |
| NRG-GI002 ^27^ | Phase II | TNT, FOLFOX+CRT | Yes | 121 | 29% | 49% |  | 28% | 25% | NR |  |
| Copernicus ^28^ | Phase II | TNT, FOLFOXx4+scRT | No | 60 | 5% | 23% | 0 | 12% |  | -- |  |
| Markovina ^29^ | Retrospective, matched | TNT scRT+FOLFOXx4 | No | 69 | 7% | 21% | NR | 28% |  | -- |  |
|  |  | CRT |  | 69+41 | 6% | 21% | NR | 16% |  | -- |  |
| Benlice ^30^ | Retrospective propensity score | TNT, scRT+ chemotherapy | No | 53 | 27% | NR | NR | 21% |  |  | 48 scRT-treated matched with 48 CRT-treated, no difference pCR 21% vs 19% |
|  |  | TNT, CRT+ chemotherapy |  | 128 | 17% | NR | NR | 20% |  |  |  |
|  |  | CRT |  | 164 | 25% | NR | NR | 15% |  |  |  |
| Moyer ^31^ | Retrospective | TNT FOLFOXx8/CAPOXx5+CRT | Yes | 84 | 19% | NR | NR | 28% |  | 49% | Compared patients at two hospitals |
|  |  | TNT scRT+FOLFOXx8/CAPOXx5 |  | 83 | 27% | NR | NR | 11% |  | 53% |  |
| Cercek ^32^ | Retrospective | TNT 4 month induction chemotherapy+CRT | Yes | 308 | 6% | NR | NR | 18% |  | 36% | Patients from a comprehensive cancer centre |
|  |  | CRT |  | 320 | 12% | NR | NR | 17% |  | 21% |  |
| Kim, MSKCC^33^ | Retrospective | TNT induction 5FUOx+ CRT | Yes | 311 | 13% | NR | NR | -- |  | 27% |  |
|  |  | CRT |  | 313 | 6% | NR | NR | -- |  | 20% |  |
| LARCT-US | Prospective observation study | TNT scRT+CAPOX x4 | Yes | 273 | 53% | 64% | 72% | -- | -- | 24% | Present nationwide studies |
| AdmL | Register study | TNT scRT+CAPOX x4 | Yes | 189 | 59% | 50+% | 82% | -- | -- | 23% |  |

*The first pCR column presents frequencies as reported in the publications in patients having primary surgery. To be more comparable to the LARCT-US/AdmL cohort, where responses are evaluated in the intention-to-treat (ITT) population, an attempt to make such an estimation based upon reported data were made. The third column presents the sum of pCR or cCR (entered W&W) in the ITT population.

Since the quality of the pathology examination of the resected specimen as well as the clinical evaluation behind a W&W decision if a cCR vary considerably, it is notoriously difficult to make these interstudy comparisons. Near complete remissions were sometimes included and the duration was not always considered. Further, staging was not always up-to-date MRI in all patients, making direct comparisons even more difficult.

**References**

1. Hammarstrom K, Mezheyeuski A, Korsavidou Hult N, Sjoblom T, Glimelius B. Stage distribution utilizing magnetic resonance imaging in an unselected population of primary rectal cancers. *Eur J Surg Oncol* 2018; **44**(12): 1858-64.

2. Bahadoer R, Dijkstra E, van Etten B, et al. Short-course radiotherapy followed by chemotherapy before total mesorectal excision (TME) versus preoperative chemoradiotherapy, TME, and optional adjuvant chemotherapy in locally advanced rectal cancer (RAPIDO): a randomised, open-label, phase 3 trial. *Lancet Oncol* 2021; **22**(1): 29-42.

3. van der Valk MJM, Marijnen CAM, van Etten B, et al. Compliance and tolerability of short-course radiotherapy followed by preoperative chemotherapy and surgery for high-risk rectal cancer - Results of the international randomized RAPIDO-trial. *Radiother Oncol* 2020; **147**: 75-83.

4. Dijkstra EA, Hospers GAP, Kranenbarg EM, et al. Quality of life and late toxicity after short-course radiotherapy followed by chemotherapy or chemoradiotherapy for locally advanced rectal cancer - The RAPIDO trial. *Radiother Oncol* 2022; **171**: 69-76.

5. Jin J, Tang Y, Hu C, et al. Multicenter, Randomized, Phase III Trial of Short-Term Radiotherapy Plus Chemotherapy Versus Long-Term Chemoradiotherapy in Locally Advanced Rectal Cancer (STELLAR). *J Clin Oncol* 2022; **40**(15): 1681-92.

6. Bujko K, Wyrwicz L, Rutkowski A, et al. Long-course oxaliplatin-based preoperative chemoradiation versus 5 x 5 Gy and consolidation chemotherapy for cT4 or fixed cT3 rectal cancer: results of a randomized phase III study. *Ann Oncol* 2016; **27**(5): 834-42.

7. Conroy T, Bosset JF, Etienne PL, et al. Neoadjuvant chemotherapy with FOLFIRINOX and preoperative chemoradiotherapy for patients with locally advanced rectal cancer (UNICANCER-PRODIGE 23): a multicentre, randomised, open-label, phase 3 trial. *Lancet Oncol* 2021; **22**(5): 702-15.

8. Schrag D, Shi Q, Weiser MR, et al. Preoperative Treatment of Locally Advanced Rectal Cancer. *N Engl J Med* 2023; **389**(4): 322-34.

9. Fokas E, Schlenska-Lange A, Polat B, et al. Chemoradiotherapy Plus Induction or Consolidation Chemotherapy as Total Neoadjuvant Therapy for Patients With Locally Advanced Rectal Cancer: Long-term Results of the CAO/ARO/AIO-12 Randomized Clinical Trial. *JAMA Oncol* 2022; **8**(1): e215445.

10. Deng Y, Chi P, Lan P, et al. Modified FOLFOX6 With or Without Radiation Versus Fluorouracil and Leucovorin With Radiation in Neoadjuvant Treatment of Locally Advanced Rectal Cancer: Initial Results of the Chinese FOWARC Multicenter, Open-Label, Randomized Three-Arm Phase III Trial. *J Clin Oncol* 2016; **34**(27): 3300-7.

11. Deng Y, Chi P, Lan P, et al. Neoadjuvant Modified FOLFOX6 With or Without Radiation Versus Fluorouracil Plus Radiation for Locally Advanced Rectal Cancer: Final Results of the Chinese FOWARC Trial. *J Clin Oncol* 2019; **37**(34): 3223-33.

12. Fernandez-Martos C, Pericay C, Aparicio J, et al. Phase II, randomized study of concomitant chemoradiotherapy followed by surgery and adjuvant capecitabine plus oxaliplatin (CAPOX) compared with induction CAPOX followed by concomitant chemoradiotherapy and surgery in magnetic resonance imaging-defined, locally advanced rectal cancer: Grupo cancer de recto 3 study. *J Clin Oncol* 2010; **28**(5): 859-65.

13. Borg C, Andre T, Mantion G, et al. Pathological response and safety of two neoadjuvant strategies with bevacizumab in MRI-defined locally advanced T3 resectable rectal cancer: a randomized, noncomparative phase II study. *Ann Oncol* 2014; **25**(11): 2205-10.

14. Moore J, Price T, Carruthers S, et al. Prospective randomized trial of neoadjuvant chemotherapy during the 'wait period' following preoperative chemoradiotherapy for rectal cancer: results of the WAIT trial. *Colorectal Dis* 2017; **19**(11): 973-9.

15. Kim SY, Joo J, Kim TW, et al. A Randomized Phase 2 Trial of Consolidation Chemotherapy After Preoperative Chemoradiation Therapy Versus Chemoradiation Therapy Alone for Locally Advanced Rectal Cancer: KCSG CO 14-03. *Int J Radiat Oncol Biol Phys* 2018; **101**(4): 889-99.

16. Marechal R, Vos B, Polus M, et al. Short course chemotherapy followed by concomitant chemoradiotherapy and surgery in locally advanced rectal cancer: a randomized multicentric phase II study. *Ann Oncol* 2012; **23**(6): 1525-30.

17. Pesantez D, Ten Hoorn S, Machado I, et al. Total neoadjuvant therapy with or without aflibercept in rectal cancer: 3-year results of GEMCAD-1402. *J Natl Cancer Inst* 2023; **115**(12): 1497-505.

18. Garcia-Aguilar J, Patil S, Gollub MJ, et al. Organ Preservation in Patients With Rectal Adenocarcinoma Treated With Total Neoadjuvant Therapy. *J Clin Oncol* 2022; **40**(23): 2546-56.

19. Rouanet P, Rullier E, Lelong B, et al. Tailored Strategy for Locally Advanced Rectal Carcinoma (GRECCAR 4): Long-term Results From a Multicenter, Randomized, Open-Label, Phase II Trial. *Dis Colon Rectum* 2022; **65**(8): 986-95.

20. Rouanet P, Rullier E, Lelong B, et al. Tailored Treatment Strategy for Locally Advanced Rectal Carcinoma Based on the Tumor Response to Induction Chemotherapy: Preliminary Results of the French Phase II Multicenter GRECCAR4 Trial. *Dis Colon Rectum* 2017; **60**(7): 653-63.

21. Sclafani F, Brown G, Cunningham D, et al. PAN-EX: a pooled analysis of two trials of neoadjuvant chemotherapy followed by chemoradiotherapy in MRI-defined, locally advanced rectal cancer. *Ann Oncol* 2016; **27**(8): 1557-65.

22. Myerson RJ, Tan B, Hunt S, et al. Five fractions of radiation therapy followed by 4 cycles of FOLFOX chemotherapy as preoperative treatment for rectal cancer. *Int J Radiat Oncol Biol Phys* 2014; **88**(4): 829-36.

23. Marco MR, Zhou L, Patil S, et al. Consolidation mFOLFOX6 Chemotherapy After Chemoradiotherapy Improves Survival in Patients With Locally Advanced Rectal Cancer: Final Results of a Multicenter Phase II Trial. *Dis Colon Rectum* 2018; **61**(10): 1146-55.

24. Nogue M, Salud A, Vicente P, et al. Addition of bevacizumab to XELOX induction therapy plus concomitant capecitabine-based chemoradiotherapy in magnetic resonance imaging-defined poor-prognosis locally advanced rectal cancer: the AVACROSS study. *Oncologist* 2011; **16**(5): 614-20.

25. Ng SP, Chu J, Chander S, et al. Results of phase II trial of intensified neoadjuvant treatment with interdigitating radiotherapy and chemotherapy with oxaliplatin, 5-fluorouracil and folinic acid in patients with locally advanced rectal cancer (PROARCT trial). *Radiother Oncol* 2021; **155**: 27-32.

26. Chin RI, Roy A, Pedersen KS, et al. Clinical Complete Response in Patients With Rectal Adenocarcinoma Treated With Short-Course Radiation Therapy and Nonoperative Management. *Int J Radiat Oncol Biol Phys* 2022; **112**(3): 715-25.

27. Hall WA, Li J, You YN, et al. Prospective Correlation of Magnetic Resonance Tumor Regression Grade With Pathologic Outcomes in Total Neoadjuvant Therapy for Rectal Adenocarcinoma. *J Clin Oncol* 2023; **41**(29): 4643-51.

28. Gollins S, West N, Sebag-Montefiore D, et al. A prospective phase II study of pre-operative chemotherapy then short-course radiotherapy for high risk rectal cancer: COPERNICUS. *Br J Cancer* 2018; **119**(6): 697-706.

29. Markovina S, Youssef F, Roy A, et al. Improved Metastasis- and Disease-Free Survival With Preoperative Sequential Short-Course Radiation Therapy and FOLFOX Chemotherapy for Rectal Cancer Compared With Neoadjuvant Long-Course Chemoradiotherapy: Results of a Matched Pair Analysis. *Int J Radiat Oncol Biol Phys* 2017; **99**(2): 417-26.

30. Benlice C, Koc MA, Gulcu B, et al. Short-Course Radiotherapy Followed By Consolidation Chemotherapy Is Safe and Effective In Locally Advanced Rectal Cancer: Comparative Short-term Results of Multicenter Propensity Score Case-Matched Study. *Dis Colon Rectum* 2023; **66**(5): 681-90.

31. Moyer AM, Vogel JD, Lai SH, et al. Total Neoadjuvant Therapy in Rectal Cancer: Multi-center Comparison of Induction Chemotherapy and Long-Course Chemoradiation Versus Short-Course Radiation and Consolidative Chemotherapy. *J Gastrointest Surg* 2023.

32. Cercek A, Roxburgh CSD, Strombom P, et al. Adoption of Total Neoadjuvant Therapy for Locally Advanced Rectal Cancer. *JAMA Oncol* 2018; **4**(6): e180071.

33. Kim JK, Marco MR, Roxburgh CSD, et al. Survival After Induction Chemotherapy and Chemoradiation Versus Chemoradiation and Adjuvant Chemotherapy for Locally Advanced Rectal Cancer. *Oncologist* 2022; **27**(5): 380-8.

**Table S13. Data sharing statement**

| Will individual participant data be available (including data dictionaries)? | Yes |
| --- | --- |
| What data in particular will be shared? | All of the individual participant data collected during the trial, after de-identification. |
| Additional information about the data: | Data on radiotherapy, chemotherapy, surgery, pathology, quality of life, follow-up as registered in SCRCR. |
| What other documents will be available? | Study protocol, publications. |
| When will data be available (start and end dates)? | Beginning at the earliest as the autumn of 2024, after finishing manuscript writing; no end date. |
| To whom will the data be available? | Qualifying researchers who submit a proposal with a valuable research question |
| For what types of analyses? | To achieve aims in the approved proposal |
| By what mechanism will data be made available? | Research proposals should be directed to professor Bengt Glimelius, principal investigator, bengt.glimelius@igp.uu.se. Data requestors will require to sign a Data Sharing Agreement. |
