# Supplementary material for: Total neoadjuvant treatment using short-course radiotherapy and four CAPOX cycles in locally advanced rectal cancer with high-risk criteria for recurrence: a Swedish nationwide cohort study (LARCT-US)
Source: eClinicalMedicine. 2024 Aug 5;75:102771. doi: 10.1016/j.eclinm.2024.102771 (PMC11577565; doi:10.1016/j.eclinm.2024.102771)
Supplement: Protocol [file mmc2.pdf]

AKADEMISKA SJUKHUSET

UPPSALA UNIVERSITET

Phase II study of short course radiation therapy followed by pre-operative chemotherapy and surgery in primary high-risk rectal cancer

LARCT- US

Locally Advanced Rectal Cancer Trial – Uppsala Style

Principal Investigator B. Glimelius

Investigators Åke Berglund, Nina Cavalli-Björkman, Tanweera Khan, Ylva Naeser, Marjut Niinivirta, Peter Nygren, Calin Radu and Gustav Ullenhag for The Gastrointestinal cancer unit, Dept of Oncology, Akademiska sjukhuset, Uppsala, Sweden

2016-06-22	**Protocol version 1.0 submitted to Research Ethical Committee**
2016-09-21	**Protocol version 1.1**
2017-03-30	**Protocol version 1.2**
2018-06-14	**Protocol version 1.3**

Table of contents

TABLE OF CONTENTS **2**

PROTOCOL SIGNATURE SHEET **4**

SYNOPSIS OF THE PROTOCOL **5**

ABBREVIATIONS **9**

BACKGROUND AND INTRODUCTION **10**

1.1 EPIDEMIOLOGY 10
1.2 TREATMENT OF RECTAL CANCER 10
1.2.1 Surgery *10*
1.2.2 Radiotherapy *11*
1.2.3 Chemotherapy *11*
1.3 RECTAL CANCER STAGING AND RISK EVALUATION 11
1.4 MOTIVATION FOR THE RAPIDO TRIAL OF PRE-OPERATIVE CHEMOTHERAPY IN RECTAL CANCER 12
1.4.1 Systemic relapses *12*
1.4.2 Locoregional therapy *13*
1.5 DESIGN CONSIDERATIONS OF A TRIAL OF PRE-OPERATIVE CHEMOTHERAPY IN RECTAL CANCER (THE RAPIDO TRIAL) 14
1.6 DESIGNS OF THE RAPIDO TRIAL AND THE FOLLOW-UP TRIAL LARCT-US WHILE WAITING FOR THE RESULTS 15
1.7 PRIMARY OBJECTIVE 17
1.8 SECONDARY OBJECTIVES 17
1.9 ENDPOINTS 17
1.9.1 Primary endpoint *17*
1.9.2 Secondary endpoints *17*

2 TRIAL DESIGN **18**

3 PATIENT SELECTION CRITERIA **19**

3.1 PRIMARY TUMOUR CHARACTERISTICS 19
3.2 GENERAL 19
3.3 EXCLUSION CRITERIA 20
3.4 COMMENTS TO THE INCLUSION CRITERIA WITH AN ASSESSMENT OF RISKS 20

4 THERAPEUTIC REGIMENS, EXPECTED TOXICITY, DOSE MODIFICATIONS **21**

4.1 CHEMOTHERAPY AND RADIOTHERAPY 21
4.1.1 Radiotherapy *21*
4.1.2 Chemotherapy *22*
4.1.3 Dose modification schedules *22*
4.1.4 Oxaliplatin *24*
4.1.5 Status of non-haematological toxicity at the start of each treatment cycle. *26*
4.1.6 Status of haematological toxicity at the start of each treatment cycle. *26*
4.1.7 At the start of each treatment cycle. *26*
4.1.8 Permanent discontinuation of individual drugs due to toxicity *26*
4.1.9 Prophylactic treatments *26*
4.2 SURGERY 27

5 CLINICAL EVALUATION, LABORATORY TESTS, FOLLOW-UP **28**

5.1 BEFORE TREATMENT START 28
5.1.1 Eligibility evaluation *28*
5.1.2 Obstructing tumours *28*
5.2 DURING TREATMENT 28
5.2.1 Experimental arm (5 x 5 Gy, neo-adjuvant chemotherapy, surgery, ... *28*
5.3 STOPPING RULES DUE TO CHEMOTHERAPY TOXICITY 29
5.4 RESECTION AND RESPONSE EVALUATION 29
5.5 PATHOLOGIC EVALUATION OF THE RECTAL CANCER RESECTION SPECIMEN 30
5.5.1 Quality of resection evaluation *30*

5.5.2 *Tumour regression score* ... *31*
5.6 After the end of treatment: Follow-up ... 31
5.6.1 *Requirements for Follow-Up* ... *31*
5.6.2 *Assessment of Recurrent Disease* ... *31*
5.7 Treatment Summary table ... 33

6 CRITERIA OF EVALUATION ... 34

6.1 Definitions ... 34
6.2 Statistical considerations ... 35
6.2.1 *Sample size* ... *35*
6.3 Interim Analyses ... 35

7 TRANSLATIONAL RESEARCH ... 35

PATIENT REGISTRATION ... 36

8 FORMS AND PROCEDURES FOR COLLECTING DATA ... 36

8.1 Case report forms (modified from the RAPIDO trial) ... 36
8.2 Data flow ... 36

REPORTING ADVERSE EVENTS ... 37

8.3 Section 10 WHO event ... 37
8.4 Adverse events and serious adverse events ... 37
8.5 Suspected unexpected serious adverse reactions (SUSAR) ... 37
8.6 Follow-up of adverse events ... 38

9 QUALITY ASSURANCE ... 38

9.1 Control of data consistency ... 38
9.2 Audits ... 38
9.3 Review of pathology ... 38
9.4 Other review procedures ... 38

10 ETHICAL CONSIDERATIONS ... 38

10.1 Patient protection ... 38
10.2 Subject identification ... 39
10.3 Informed consent ... 39

11 PUBLICATION POLICY ... 39

REFERENCES ... 39

APPENDIX E - PATIENT INFORMATION AND INFORMED CONSENT LARCTUS ... 42

Protocol Signature Sheet

We declare that we have read and familiarized ourselves with the following protocol:

Phase II study of short course radiation therapy followed by pre-operative chemotherapy and surgery in primary high-risk rectal cancer

LARCT - US

**Name**	**Site**	**Signature**	**Date**
**Study Coordinators**	Uppsala	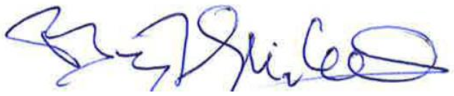	June 14, 2018

Synopsis of the protocol

**Protocol title**	Phase II study of short course radiation therapy followed by pre-operative chemotherapy and surgery in patients with high-risk primary rectal.
**Protocol Phase**	Final
**Indication**	Primary rectal cancer with high risk of failing locally and/or systemically
**Background**	In patients with a newly diagnosed rectal cancer at high risk of failing locally and/or systemically standard therapy long-course preoperative radiotherapy with concomitant chemotherapy followed by surgery after 6-8 weeks has been standard therapy after a previous Nordic study in locally advanced rectal cancer. Despite lack of strong scientific evidence, postoperative adjuvant chemotherapy during 6 months has been added in many centres.<br>In a randomized study, the RAPIDO trial, this chemoradiotherapy (CRT) has been compared with an experimental treatment starting with short-course radiotherapy (RT) followed by 6 courses of neo-adjuvant chemotherapy with capecitabine and oxaliplatin (CAPOX). The trial closed patient entry early June 2016 after having randomized the planned number of 920 patients. Almost half of the patients were included in Sweden. The immediate experience with the experimental treatment is that it is well tolerated by most patients, provided that the patient does not have remaining radiation toxicity at the start of the chemotherapy. No results will be available until 2018 when some secondary variables can be analysed. The primary outcome will not be available until late 2019.<br>During the inclusion period of the RAPIDO trial, a Polish group randomized similar patients, however not all staged with MRI, to CRT or to an experimental therapy starting also with short-course RT followed by three cycles of FOLFOX, being equivalent to two cycles of CAPOX. The results were published in May 2016 [1]. They showed that the experimental treatment was better tolerated than CRT, preferred by more patients and that overall survival was improved. The latter is hard to explain since disease-free survival was not influenced. However, the experimental treatment can at least be considered equivalent to the previous standard therapy, and it has better compliance.<br>In the light of the favourable immediate experience with short-course RT followed by neo-adjuvant therapy in the RAPIDO trial together with the apparently favourable experience with a similar treatment in the randomized trial by the Polish group, the GI-oncologists in Uppsala have decided that the coming reference treatment for these patients should be short-course RT followed by chemotherapy and then surgery. The decision was to recommend four cycles of CAPOX and not six as in the RAPIDO trial or two as in the Polish trial. Since the evidence for positive effects from adjuvant postoperative chemotherapy in rectal cancer after preoperative therapy is limited, it

	was decided not to give a full period of 6 months of adjuvant therapy prior to surgery in patients fit for this therapy, but rather 3 months (corresponding to 4 cycles of CAPOX) since the present standard of 6 months of adjuvant therapy will be shortened to 3 months for some patient groups after the results of the large trials comparing 6 and 3 months of therapy (IDEA, final results released in May 2017).
**Endpoints**	**Primary endpoint:**<br>• Pathological complete response (pCR) rate<br>**Secondary endpoints:**<br>• Clinical complete response (cCR) rate (see full protocol if durable)<br>• NAR-score<br>• 3-year disease-free survival<br>• Overall survival<br>• Short and long-term toxicity
**Study design**	Patients will be treated with the short course 5 x 5 Gy radiation scheme followed by four cycles of combination chemotherapy (capecitabine and oxaliplatin) and TME surgery. In patients at risk for chemotherapy toxicity, the number of courses may be limited to two.
**Total number of centres**	Akademiska sjukhuset, Uppsala<br>Other centres in Sweden, Norway and Denmark, most participating in the RAPIDO trial have decided to adopt this regimen as reference treatment until the results of the RAPIDO trial is known, thus participating in LARCT-US
**Selection criteria**	Patients with a primary rectal cancer without detectable distant metastasis who after locoregional therapy only, meaning preoperative radio(chemo)therapy plus surgery have at least a 40% risk of not having a CRM negative resection or a recurrence, local or distant, within three years.
**Main criteria for inclusion**	**Primary tumour characteristics (identical to those in the preceding RAPIDO trial):**<br>• Histological proof of newly diagnosed primary adenocarcinoma of the rectum.<br>• Locally advanced tumour fulfilling at least one of the following criteria on pelvic MRI indicating high risk of failing locally and/or systemically (T4b, i.e., overgrowth to an adjacent organ or structure like the prostate, urinary bladder, uterus, sacrum, pelvic floor or side-wall (according to TNM version 7), cT4a, i.e., peritoneal involvement, extramural vascular invasion (EMVI+). N2, i.e., four or more lymph nodes in the mesorectum showing morphological signs on MRI indicating metastatic disease. Four or more nodes, whether enlarged or not, with a rounded, homogeneous appearance is thus not sufficient. Positive MRF (previously named CRM), i.e., tumour or lymph node $\leq$ 1 mm from the mesorectal fascia [60]. Enlarged lateral nodes, > 1 cm (lat LN+).

	**General:**<br>• Staging done within 6 weeks before start of radiotherapy.<br>• No contraindications to chemotherapy with CAPOX or FOLFOX, including adequate blood counts:<br>- white blood count ≥4.0 x 10^9/L<br>- platelet count ≥100 x 10^9/L<br>- clinically acceptable haemoglobin levels<br>- creatinine levels indicating renal clearance of ≥50 ml/min<br>- bilirubin <35 µmol/l.<br>• ECOG performance score ≤ 1.<br>• Patient is considered to be mentally and physically fit for chemotherapy as judged by the oncologist.<br>• Age ≥ 18 years<br>• Written informed consent.<br>• Adequate potential for follow-up.
**Exclusion criteria**	• See detailed description in the protocol
**Main parameters of efficacy**	**Primary:** pCR – rate. Patients who receive a clinical complete response and where surgery is postponed will also be considered<br>**Secondary:** Clinical response as assessed using MRI, digital palpation and endoscopy, disease-free survival after 3 years, overall survival and toxicity, CRM negative resection rate, NAR score
**Main parameters of safety**	Adverse events, graded according to the NCI CTCAE (version 4.0).<br>• Frequency of patients able to undergo full treatment schedule.
**Screening**	Baseline screening includes:<br>• CT (or MRI) of the abdomen and liver<br>• MRI of the pelvis<br>• CT of the thorax<br>• Routine blood tests
**Treatment**	Week 1: 5 x 5 Gy<br>Week 3-14: 4 courses of CAPOX (Capecitabine b.i.d.1000 mg/m^2 day 1-14 every 3 weeks, Oxaliplatin 130 mg/m^2 day 1 every 3 weeks)<br>Week 17-20: Surgery (TME)
**Statistical considerations**	No statistical evaluations have been performed, but the pCR rates should be at least comparable to the ones seen in multiple trials using CRT, and in the trial using 5x5 Gy with 3 cycles of FOLFOX (corresponding to 2 cycles of CAPOX) and in the same order of magnitude as will be seen in the experimental arm in the RAPIDO trial. The NAR score should aim at to be below 14.
**Planned sample size**	The number of patients to be included has not been fixed, but it is estimated to be at least 60 during the period until RAPIDO data will be available.
**Analysis plan**	The primary endpoint will be analysed when the last patient has completed treatment including the surgery. For patients who are not

	operated because of a cCR, minimal follow-up should be 12 months.
**Duration of the study**	About two-year inclusion, and at least two-year follow up after inclusion of the last patient.

Abbreviations

ANC	absolute neoutrophil count
APR	abdominoperineal resection
BED	biological effective dose
CAPOX	capecitabine and oxaliplatin
CEA	carcinoembryonic antigen
CRM	circumferential resection margin
CRF	case record form or case report form
CT	computer tomography
CTCAE	Common Terminology Criteria for Adverse Events
DPD	dihydropyrimidine dehydrogenase
DFS	disease free survival
DSMB	Data Safety Monitoring Board
ECOG	Eastern Cooperative Oncology Group
EMVI	extramural vascular invasion
EORTC	European Organisation for Research and Treatment of Cancer
5HT3	5-hydroxytryptamine (serotonin) receptor 3A
LAR	low anterior resection
IMRT	intensity-modulated radiotherapy
ICRU	international commission on radiation unit and measurements
MDT	multidisciplinary team
MRF	mesorectal fascia
MRI	magnetic resonance imaging
NCI	national cancer institute
RT	radiotherapy
OS	overall survival
pCR	pathological complete response
PET	positron emission tomography
PME	partial mesorectal excision
QoL	quality of life
SAE	serious adverse event
SUSAR	suspected unexpected serious adverse event
TNM	tumour node metastasis classification
TME	total mesorectal excision

Background and introduction

1.1 Epidemiology

Colorectal cancer is globally, and in the Western world, with about 1 million new cases annually, the third most common cancer. Its incidence is relatively stable in the western world,but increases in many developing countries. It is estimated that about 600 000 individuals every year die from colorectal cancer. In many western countries, it is the second cancer killer. In 2012, 600 new colorectal cancer patients were registered in Sweden [2]. About every third colorectal cancer starts in the rectum, or the most distal 15 cm of the large bowel. The rest starts in the colon, most frequently in the sigmoid part or in caecum. Rectal cancers are more common in males.

1.2 Treatment of rectal cancer

1.2.1 Surgery

Surgery was for long the only curative treatment and is still the most important treatment. If a macro- and microscopically radical resection (R0 resection) cannot be achieved, the chances of cure are very low. A few small tumours in the rectum can be treated with external and local radiotherapy [3] and there are indications that some, likewise small rectal cancers that are very chemoradiosensitive can be successfully handled without (major) surgery [4].

Although some early, mostly polypoid tumours without unfavourable characteristics can be operated with a local, i.e. transanal procedure, most patients with a rectal cancer are operated with an abdominal procedure with a resection of the affected bowel segment and adjacent fatty tissues with its vessels and lymph nodes. Depending upon location, standardized procedures are done, at least if the aim is cure.

Recognition of the importance of the circumferential resection margin led to the understanding that the entire mesorectum must be completely removed in one package to obtain low local failure rates [5]. The presently only accepted surgical method is to do a sharp dissection and a total mesorectal excision (TME) in all rectal cancers except in those in the upper third where at least a 5 cm distal margin within the mesorectum should be aimed at. The procedure in which the mesorectum and bowel are transected 5 cm distally of tumour is commonly termed partial mesorectal excision (PME). Most centres applying standardised TME/PME techniques can today report local failure rates of 5 to 10% in the group of patients where the intention is to do a radical procedure [6].

If the tumour involves the mesorectal fascia (MRF i.e., if a standard TME is done, there is a high risk that the circumferential resection margin will be positive, CRM+) or extends to adjacent structures or organs (T4b), a more extended procedure is required in order to reach an CRM negative resection. In certain patients, this may mean a full or partial pelvic exenteration or resection of parts of sacrum.

1.2.2 Radiotherapy

Radiotherapy has been extensively used in rectal cancer during the past decades. The purpose of adding radiotherapy to surgery has been mainly two-fold, firstly to reduce the risk of a local failure, even if an R0 surgery is considered likely and accomplished, or, secondly, to increase the chances of an R0-resection in a locally advanced tumour considered 'non-resectable'. In the first situation, a short-course schedule, like 5 x 5 Gy, with immediate surgery, is one option, since no down-sizing or down-staging is required. Data from randomized trials strongly support this approach in resectable rectal cancer [7-9]. In the second situation, long-course, conventionally fractionated (1.8 – 2 Gy/fraction) to a dose of 45 – 50.4 Gy is used with a delay prior to surgery to allow for down-sizing/staging. Concomitant chemotherapy to the long-course radiotherapy (RT) improves local control [10-12] and is thus standard treatment to patients who are suitable for this combined therapy. As an alternative to chemoradiotherapy (CRT), the short-course schedule with a delay prior to surgery has been used in unfit patients, with results that appear promising [13, 14]. This approach was used in the completed randomized trial in resectable patients (Stockholm III study) [15, 16]. The results show that downsizing and downstaging is seen after 5x5 Gy with delayed surgery that is at least as large as that seen after long-course RT to 50 Gy without chemotherapy. There are no differences in local recurrence rates, disease-free and overall survival between the groups. The surgical morbidity after 5x5 Gy with immediate surgery is higher than after delayed surgery; however, radiation induced adverse effects requiring hospitalization is seen in 5-7% of the patients in the delay groups.

1.2.3 Chemotherapy

Systemic relapses constitute a major problem in colorectal cancer. The most widely used method to decrease systemic relapse rates is to give postoperative adjuvant systemic therapy, presently chemotherapy. This approach has been successful in many tumours, such as breast and colon cancer with meaningful reductions in relapse rates and post-operative adjuvant chemotherapy in colon cancer stage III and high-risk stage II is standard treatment. Presently six months of treatment is used, but several large groups have run about five large trials comparing three and six months of oxaliplatin-containing therapy. There are no results from these comparisons but several of them have completed patient inclusion a few years ago. In rectal cancer, as opposed to colon cancer, the scientific support for sufficient activity from adjuvant chemotherapy is less strong, and its use is controversial. At many centres post-operative adjuvant chemotherapy is standard in rectal cancer but recent randomized trials have not been able to detect any significant gains if RT or CRT were given prior to surgery [17-19].

1.3 Rectal cancer staging and risk evaluation

Appropriate 'up-to-date' staging of rectal cancer includes Magnetic Resonance Imaging (MRI) of the pelvis together with imaging of the lungs, liver and abdomen to exclude distant metastases. Pelvic MRI has evolved as the method of choice since it evaluates the periphery of the tumour and its relations to the mesorectal fascia and surrounding structures better than other techniques. Positron emission tomography (PET) is also sometimes used to detect tumour manifestations not otherwise detectable [20]. Using MRI, rectal tumours can be grouped into categories having different risks of failing locally and, more recently, also systemically. A European project, The Magnetic Resonance Imaging and Rectal Cancer European Equivalence study (MERCURY) prospectively evaluated the risk of failing locally, and has published criteria dividing rectal tumours into three groups (low, intermediate and high, or 'good', 'bad' and 'ugly') [21, 22]. There is presently no international consensus about the criteria, but there is sufficient evidence to allow for identification of patients with a sufficiently high risk to fail either

locally and/or systemically to be included in a trial exploring the value of treating patients with neo-adjuvant chemotherapy.

1.4 Motivation for the RAPIDO trial of pre-operative chemotherapy in rectal cancer

Better staging, improved decision-making at multidisciplinary team (MDT) meetings, more refined surgery (TME), appropriate use of preoperative radiotherapy, being superior to postoperative CRT together with quality control (pathology and registries) have resulted in substantial lowering of local failure rates (from above 30% to below 5-10% in many populations). It can then be stated that 'the local problem in rectal cancer is in principle solved'. Although this may be true for rectal cancer patients in general, certain subgroups of patients still suffer a substantial risk of not having R0 surgery or a local failure. In addition, survival for rectal cancer patients has improved, but not nearly to the same extent as local failure rates have. Thus, it is important to study treatment approaches aimed at reducing the risk of systemic relapse without compromising local control. It is not reasonable to believe that further improvements in the loco-regional treatment of the primary will reduce the systemic relapses. The chapters below (1.4-1.5) constituted the motivation for the now recently closed RAPIDO trial, and the design is defined in chapter 1.6.

1.4.1 Systemic relapses

About 40-60% of patients with locally advanced rectal cancer (cT3c/d-4 and/or N1-2) develop distant metastases. Systemic chemotherapy aims at treating occult or micro-metastatic sub-clinical disease that later can appear as distant metastases. Current standard treatment for patients at high risk of failing locally and/or systemically includes pre-operative chemoradiation. Administration of chemotherapy concomitantly during radiotherapy improves local control in randomized trials [10-12]. In the Nordic trial cancer-specific survival was also improved [12]. However, when giving chemotherapy concomitantly toxicity increases, and dosage of chemotherapy must be reduced which may influence the systemic efficacy. In many centres post-operative adjuvant chemotherapy is prescribed to these patients but since rectal cancer surgery is associated with relatively high complication rates (e.g., anastomotic leakage in 19% [23]) many patients cannot receive chemotherapy postoperatively. In a German randomized rectal cancer trial comparing pre-operative chemoradiation to post-operative chemoradiation only 50 % of patients in the post-operative arm received full-dose chemotherapy compared to 89 % in the pre-operative arm [24].

An alternative approach is to administer the systemic therapy before surgery, which is often termed "neo-adjuvant" therapy. Support of greater efficacy from neo-adjuvant (or combined neo-adjuvant and adjuvant, so called peri-operative treatment) comes from some other tumour types, but not universally. The strongest support likely comes from gastric cancer, where peri-operative platinum-based therapy resulted in better overall survival than surgery alone (by about 13 – 14%-units), MAGIC trial [25] and FFCD trial [26]. In colorectal cancer metastatic to the liver, a gain in event-free survival was seen in an EORTC trial [27] with peri-operative chemotherapy. The difference was seen during the first 10 weeks, indicating that the preoperative part of the chemotherapy was more important than the postoperative. Neo-adjuvant treatment with chemotherapy has also been explored in e.g. head- and neck cancer and oesophageal cancer (in the MAGIC and FFCD gastric cancer trials, patients with oesophageal adenocarcinoma were also included) and muscle invasive bladder cancer [28], with unequivocal results. In early breast

cancer, it does not appear to be important to initiate the systemic therapy early, although up-front systemic chemotherapy is the treatment of choice in locally advanced breast cancer.

In the Dutch “M1 trial” short course radiation therapy, neoadjuvant chemotherapy with bevacizumab and radical resection of primary tumour and metastases in primary stage IV rectal cancer was evaluated [29]. The study included 50 patients (approximately 75 % with T3/T4/N+ tumours) who were treated with 5 x 5 Gy radiotherapy followed by 6 cycles of bevacizumab (7.5 mg/kg every 3 weeks), oxaliplatin (130 mg/m^2 every 3 weeks) and capecitabine (1000 mg daily day 1-14 mg/m^2). Eight weeks after last dose of bevacizumab surgery was performed. The completion rate for all (six) cycles of chemotherapy was 85% and more than 90 % completed at least 4 cycles. No significant tumour progression of the primary rectal cancer was observed during chemotherapy. None of the patients could not be operated on their local rectal tumour due local tumour progression. Only 1 patient had significant morbidity of the primary tumour due to the pre-operative treatment, this was caused by a perforation and pelvic abscess due to a massive tumour response with tumour necrosis. In 91% of patients a R0 resection of the primary tumour was performed. Pathological evaluation of rectal specimens showed a complete response rate of 27%. This is higher than after standard chemoradiotherapy. No severe toxicity was observed upon radiotherapy and chemotherapy related toxicity was mostly mild.

Thus, it appears reasonable to assume that pre-operative chemotherapy is more likely to be administered in full doses (giving full systemic effect) compared to concomitant or post-operative chemotherapy.

1.4.2 <u>Locoregional therapy</u>

In many centres the current standard of treatment for rectal tumours at high-risk of failing locally or non-resectable tumours is pre-operative long-course chemoradiation (1.8-2 Gy x 25-28 with capecitabine) whereas low-risk patients with resectable tumours receive short-course radiotherapy (5 Gy x 5). The biological effective dose (BED) of a fractionated radiation scheme is calculated as LQ time = $n.d(1+d/\alpha/\beta) - (\gamma/\alpha)(T-T_k)$ in which n is the number of fractions, d is the dose (Gy) per fraction, α/β is the common linear-quadratic quotient (set to 10 Gy), γ/α is the repair rate (set to 0.6 Gy/day), T is the total treatment time (days), and T_k is the initial delay time (days, set to 7 days). Using this formula, the BED of 5 x 5 Gy equals to 37.5 Gy and the BED of 28 x 1.8 Gy equals to 40.9 Gy.

In patients with more advanced tumours (e.g. T4, MRF-positive, positive lateral nodes) the pre-operative therapy aims at down-staging or down-sizing the tumour whereby the chances of performing a R0-resection are increased. Long-course radiotherapy, in particular in combination with concomitant chemotherapy increases resectability and improves local control [29]. When long-course chemoradiation is delivered, surgery is postponed for 4-8 weeks allowing for acute radiation-induced tissue reactions to settle prior to surgery and this “waiting period” also allows for down-sizing to occur [30]. When short-course radiotherapy is used, surgery is generally performed the following week without a “waiting period” and it has been questioned whether any down-staging occurs following this regimen. A Polish trial demonstrated that significantly more down staging occurred when a conventional radiotherapy scheme (50.4 Gy, surgery after 4-6 weeks) combined with chemotherapy (5-FU/leucovorin) was compared with short-term preoperative radiotherapy (5 x 5 Gy, surgery within 7 days), but with no difference in local recurrence rate and survival [31]. Similar results were reported from an Australasian trial [32]. The Stockholm III trial randomized patients with resectable rectal cancer to either long-course radiotherapy (50 Gy), short-course radiotherapy with immediate surgery or short-course radiotherapy with delayed surgery (4-8 weeks “waiting period”) and data have demonstrated that short-course radiotherapy with delayed surgery is feasible, result is low toxicity and causes down-staging [16, 33]. Retrospective observational data have shown that short-course

radiotherapy with delayed surgery can produce significant down-staging and also pCR) in some patients [13, 14, 34].

In the M1 trial where systemic chemotherapy was administered after short-course before surgery no significant local tumour progression during chemotherapy was seen [29]. As stated above in 11 of 41 (27%) resected rectal specimens a pCR was observed.

Thus, data to support that short-course pre-operative radiotherapy with delayed surgery is feasible and that down-staging or down-sizing may occur following this regimen are present in the literature. Furthermore, the interval between radiotherapy and surgery can be prolonged and if chemotherapy is delivered in this interval, significant effects can be seen on the primary rectal tumour.

1.5 Design considerations of a trial of pre-operative chemotherapy in rectal cancer (the RAPIDO trial)

In patients with a newly diagnosed rectal cancer at high risk of failing locally and/or systemically standard therapy is long-course preoperative radiotherapy with concomitant chemotherapy followed by surgery after 4-8 weeks. Despite lack of indisputable scientific evidence, postoperative adjuvant chemotherapy is added in many centres.

To achieve higher compliance and better effect of chemotherapy, the aim is to deliver the systemic treatment pre-operatively. Most standard adjuvant chemotherapy schedules in colorectal cancer have a duration of 24 weeks. Modifications of current standard therapy could theoretically include increase of dose or number of chemotherapy agents for the concomitant therapy but that would increase toxicity and possibly decrease compliance. To postpone all locoregional therapy in order to start with systemic chemotherapy would not gain acceptance because of the risk of local progression.

A peri-operative chemotherapy regimen was successfully explored for liver metastases of colorectal cancer in the EORTC-EPOC trial and with a similar schedule a trial with an experimental arm consisting of 12 weeks of chemotherapy pre-operatively followed by short-course radiotherapy and immediate surgery and 12 weeks of post-operative chemotherapy could be considered [27]. This design would, however, have some drawbacks including no locoregional therapy initially and the risk of not being able to deliver half of the chemotherapy to a substantial proportion of the patients. Moreover, when surgery is performed immediately after radiotherapy, the desired down-staging on these locally advanced tumours may not occur, leading to a potential risk of decreased local control rates.

Another alternative is to explore possibilities connected with using the short-course radiotherapy with delayed surgery as the locoregional therapy. One of the advantages of the short-course schedule is the low toxicity (in particular acute toxicity) which implies that a vast majority of patients would be able to start full-dose systemic chemotherapy a week or two after radiotherapy. Data from the retrospective trials [13, 14]and the M1 trial [29] support the notion that systemic chemotherapy also acts on the primary tumour, thus leading to improved locoregional therapy as compared to short-course and a “waiting period”. However, in order to reduce the interval between radiotherapy and surgery and still being able to deliver all systemic chemotherapy prior to surgery, adjustments of standard chemotherapy schedules for colorectal cancer may be necessary. The schedule explored in the M1 trial consisting of 18 weeks with oxaliplatin/capecitabine is 6 weeks (2 cycles) shorter than commonly used in post-operative adjuvant schedules and offers an attractive alternative. Bevacizumab was included in the “M1

trial” but there is no data suggesting that bevacizumab or cetuximab improves the antitumour effects against subclinical disease.

1.6 Designs of the RAPIDO trial and the follow-up trial LARCT-US while waiting for the results

RAPIDO was a randomized multicentre Phase III trial in patients with non-metastatic primary rectal cancer with a high risk of failing locally and/or systemically. Standardised MRI criteria will be used to identify eligible patients. Patients were randomized between a standard therapy arm (A) and an experimental arm (B).

A: Long-course radiotherapy (1.8-2 Gy x 25-28) with concomitant capecitabine. After a “waiting period” of 8-10 weeks during which response is evaluated, surgery according to TME/PME principles will be performed. In this arm it is allowed according to the local protocol of the participating institute to admit after recovery, optimally within 6-8 weeks, post-operative adjuvant chemotherapy consisting of 8 cycles oxaliplatin/capecitabine.

B: Short-course radiotherapy (5 Gy x 5). Within 11-18 days after the last day of radiotherapy pre-operative systemic chemotherapy with oxaliplatin/capecitabine will commence and is delivered in 6 cycles. After six cycles response is evaluated. Within 2-4 weeks after the final chemotherapy cycle surgery according to TME/PME principles will be performed. No postoperative therapy will be given.

LARCT-US

The RAPIDO trial closed patient entry early June 2016 after having randomized the planned number of 920 patients. 106 patients were included at Akademiska sjukhuset, Uppsala, being the largest centre. The immediate experience with the experimental treatment is that it is well tolerated by most patients, provided that the patient does not have remaining radiation toxicity at the start of the chemotherapy. No results are available until 2017 when only results of some secondary variables can be analysed. The primary outcome will not be available until late 2019 at the earliest.

During the inclusion period of the RAPIDO trial, a Polish group randomized similar patients, however not all staged with MRI, to CRT or to an experimental therapy starting also with short-course RT followed by three cycles of FOLFOX, being equivalent to two cycles of CAPOX. The results were presented in Ann Oncol in May 2016 [1]. They showed that the experimental treatment was better tolerated than CRT, preferred by more patients and that overall survival was improved. The latter is hard to explain since disease-free survival was not influenced. However, the experimental treatment can at least be considered equivalent to the previous standard therapy and with better compliance.

In the light of the favourable immediate experience with short-course RT followed by neo-adjuvant therapy in the RAPIDO trial together with the apparently favourable experience with a similar treatment in a randomized trial by the Polish group, the GI-oncologists in Uppsala decided that the coming reference treatment for these patients should be short-course RT followed by chemotherapy and then surgery. The decision was to recommend four cycles of CAPOX and not six as in the RAPIDO trial or two as in the Polish trial. Since the evidence for positive effects from adjuvant postoperative chemotherapy in rectal cancer after preoperative therapy is limited, it was decided not to give a full 6 months period of adjuvant chemotherapy prior to surgery in patients fit for this therapy, but rather 3 months since it can be expected that the present standard postoperatively of 6 months of adjuvant therapy will be shortened to 3

months after the results of the large trials comparing 6 and 3 months of therapy (IDEA, final results were released in May 2017 and have been published in full in 2018 (Grothey et al, NEJM 2018; Iveson et al Lancet Oncol 2018; Sobrero et al JCO 2018). The results briefly showed that 3 months of chemotherapy is not inferior, at least not for large groups of patients, to 6 months of therapy. In patients that are not expected to tolerate the planned four treatments, e.g. because of very high age or co-morbidities, two cycles can be given.

This treatment will be given within a prospective phase II trial to be able to evaluate the results properly and compare them with those reported by the Polish group and the coming results in the RAPIDO trial. A formalized protocol will likely also result in that the treatments are given with higher quality. The inclusion criteria, and routines in the trial will be identical to the RAPIDO trial, facilitating future comparisons. The inclusion criteria are also similar to those in the Polish study, although inter-trial comparisons are always difficult to interpret.

A large series of US patients with rectal cancer treated with CRT recently reported that patients could be divided into three prognostic groups according to the pretreatment stage and pathological stage {George, 2015 #13175}. The score (neo-adjuvant rectal, NAR, score) considers the pathological N-stage and the difference between clinical and pathological T-stage according to the following formulation:

$$NAR = \frac{[5\,pN - 3(cT - pT) + 12]^2}{9.61}$$

The NAR score has recently been prospectively evaluated or validated in other patient materials (Fokas et al Ann Oncol 2018; Rosello et al Clin Colorectal Cancer 2018), and is used by a large US co-operative group (NRG) to prospectively identify the best neo-adjuvant treatment, replacing CRT. A NAR score of 14 or less is then considered promising in a phase II study, motivating further testing.

The LARCT-US protocol has been used in Uppsala since August 2016 and has since attracted other clinics in Sweden to adopt the same idea while waiting for the RAPIDO results. Ethical approval for participating in the trial has been obtained from more and more centres since then, even if the committee in Uppsala considered this as routine therapy not formally requiring approval before being used.

1.7 Primary objective

- To investigate the complete pathological remission rate.

1.8 Secondary Objectives

- To describe the toxicity profile of the combined modality treatment in schedule.
- To determine the completion rate of the neo-adjuvant treatment.
- To determine the fraction of patients with a radical resection (negative CRM)
- To determine the clinical response rate as assessed at the MRI-investigation, palpation and endoscopy after the preoperative therapy
- To determine the postoperative complications
- To describe the local recurrence rate after 3 years follow-up.
- To describe disease-free and overall survival after 3 and 5 years of follow-up.

1.9 Endpoints

1.9.1 <u>Primary endpoint</u>

Pathological complete remission (pCR).

1.9.2 <u>Secondary endpoints</u>

- Treatment associated toxicity, including surgical morbidity
- Completion rate of neo-adjuvant treatment
- Negative CRM (margin > 1 mm)
- Clinical complete response (cCR) evaluated using MRI palpation and endoscopy. In patients where surgery is not primarily done, cCR with a duration exceeding 12 months will be considered together with the pCR rates as primary endpoint.
- Postoperative complications
- Local recurrence at 3 years
- Disease-free and overall survival

For an exact definition of the parameters used as end-points, and the detailed method of assessment: see section **6.**

2 Trial design

This trial is a prospective phase II trial, initially run at one centre (Uppsala) but later expanded to other centres.

Patients will be treated with short course 5 x 5 Gy radiation scheme followed by four (two in risk patients for toxicity) cycles of combination chemotherapy (capecitabine and oxaliplatin) and surgery. The rectal tumour will be removed by TME/PME surgery or more extensive surgery if required because of tumour extent.

Treatment Plan:

Study Flow Chart

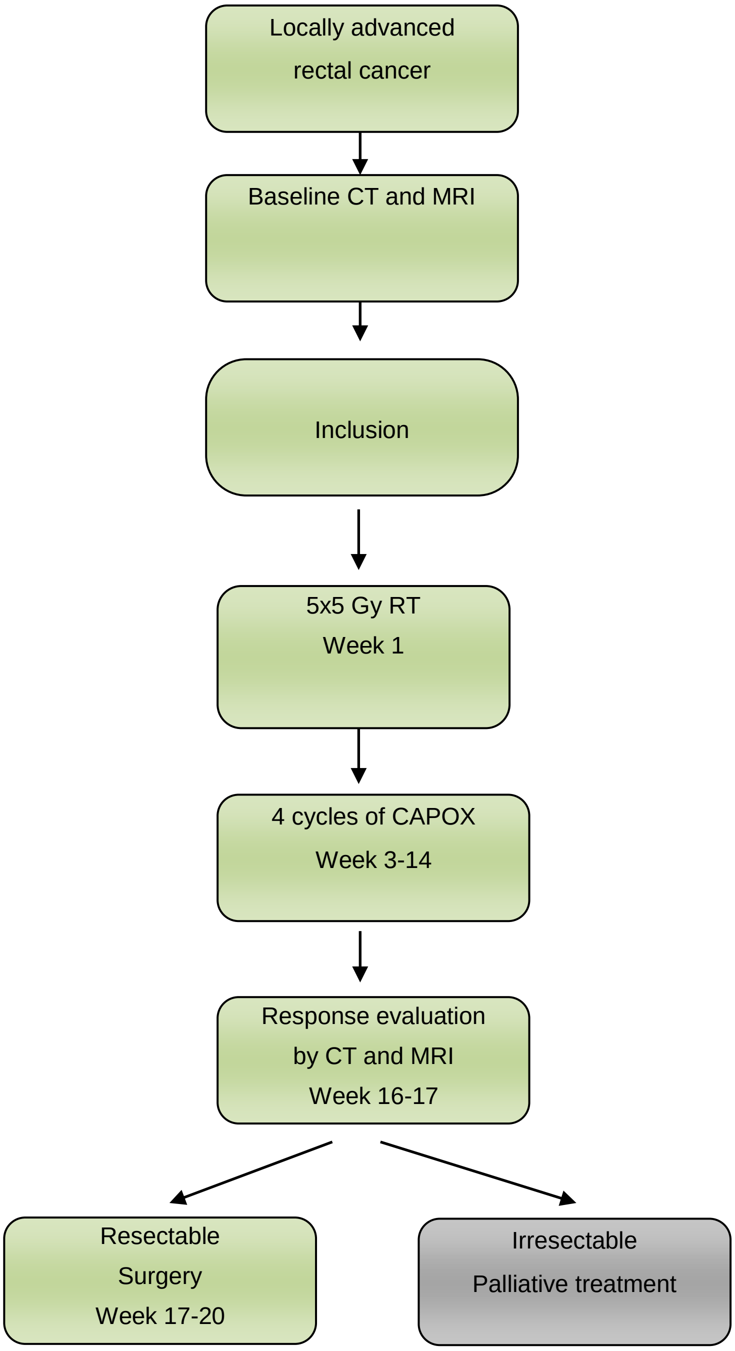

3 Patient selection criteria

3.1 Primary tumour characteristics

- Biopsy-proven, newly diagnosed primary rectal adenocarcinoma, i.e., with the lowest part of the tumour less than 16 cm from the anal verge using a rigid rectoscope or flexible endoscope.
- Locally advanced tumour fulfilling at least one of the following criteria on pelvic MRI indicating high risk of failing locally and/or systemically:
    - Clinical stage (c) T4a, i.e. overgrowth to an adjacent organ or structure like the prostate, urinary bladder, uterus, sacrum, pelvic floor or side-wall (according to TNM version 5).
    - cT4b, i.e., peritoneal involvement.
    - Extramural vascular invasion (EMVI+).
    - N2, i.e., four or more lymph nodes in the mesorectum showing morphological signs on MRI indicating metastatic disease. Four or more nodes, whether enlarged or not, with a rounded, homogeneous appearance is thus not sufficient.
    - Positive MRF, i.e., tumor or lymph node one mm or less from the mesorectal fascia.
    - Metastatic lateral nodes, > 1 cm (lat LN+), see appendix G (see the RAPIDO protocol).

3.2 General

- Staging done within 6 weeks before start of radiotherapy. No contraindications to chemotherapy with CAPOX including adequate blood counts, (within 5 weeks prior to randomisation):

  - white blood count ≥4.0 x 10^9/L
  - platelet count ≥100 x 10^9/L
  - clinically acceptable haemoglobin levels
  - creatinine levels indicating renal clearance of ≥50 ml/min
  - bilirubin <35 µmol/l.

- ECOG performance score ≤ 1, see appendix **B** (see the RAPIDO protocol).

- Patient is considered to be mentally and physically fit for chemotherapy with CAPOX as judged by the oncologist.

- Age ≥ 18 years

- Written informed consent.

- Adequate potential for follow-up.

3.3 Exclusion criteria

- Extensive growth into cranial part of the sacrum (above S3) or the lumbosacral nerve roots indicating that surgery will never be possible even if substantial tumour down-sizing is seen.

- Presence of metastatic disease or recurrent rectal tumour. Familial Adenomatosis Polyposis coli (FAP), Hereditary Non-Polyposis Colorectal Cancer (HNPCC), active Crohn's disease or active ulcerative Colitis.
- Concomitant malignancies, except for adequately treated basocellular carcinoma of the skin or in situ carcinoma of the cervix uteri. Subjects with prior malignancies must be disease-free for at least 5 years.
- Known DPD deficiency.
- Any contraindications to MRI (e.g., patients with pacemakers).
- Medical or psychiatric conditions that compromise the patient's ability to give informed consent.
- Concurrent uncontrolled medical conditions.
- Any investigational treatment for rectal cancer within the past month.
- Pregnancy or breast feeding.
- Patients with known malabsorption syndromes or a lack of physical integrity of the upper gastrointestinal tract.
- Clinically significant (i.e. active) cardiac disease (e.g. congestive heart failure, symptomatic coronary artery disease and cardiac dysrhythmia, e.g. atrial fibrillation, even if controlled with medication) or myocardial infarction within the past 12 months.
- Patients with symptoms of peripheral neuropathy.

3.4 Comments to the inclusion criteria with an assessment of risks

The presence of one or more of the risk factors indicates that the estimated risk of failing (no CRM negative resection, local pelvic or systemic recurrence) within 3 years is 60% or above if surgery is the primary treatment and 40% or above if radiotherapy with 5FU chemotherapy followed by surgery (and adjuvant chemotherapy) is the primary treatment. It is assumed that at least a TME/PME is performed. In patients with overgrowth to adjacent organs or structures, these are removed *en bloc.*

The criteria mentioned all indicate that the risk of systemic relapse is high, whereas not all indicate that the risk of failing locally is high.

4 Therapeutic regimens, expected toxicity, dose modifications

4.1 Chemotherapy and radiotherapy

4.1.1 Radiotherapy

Preoperative radiotherapy will be delivered on a linear accelerator in prone or supine position, preferably with full bladder. The use of a belly board is allowed. Isocentric 3 or 4 beams, as well as an IMRT technique is allowed, as long as all beams are treated on a daily basis.

The dose distribution and calculation should be performed on CT scans or MRI scans and specified according to the ICRU 50 guidelines.

4.1.1.1 Dose specification

All patients will receive 5 daily fractions of 5 Gy up to a total dose of 25 Gy. Overall treatment time should be maximum eight days.

A boost dose to the tumour bed is optional.

4.1.1.2 Target volume

Pelvic field (see Appendix **F** for further details found in the RAPIDO protocol)

Tumour bed with a margin, plus regional lymph nodes according to tumour location and growth. The mesorectal and pre-sacral lymph nodes are always included whereas the lateral obturator nodes and internal iliac nodes are only included if the tumour grows below the peritoneal reflection. The external iliac nodes should be included if the primary tumour invades the bladder, prostate, cervix or vagina to such an extent that the external nodes are at risk for metastases. Napping or minimal overgrowth dorsally is not sufficient.

If it is decided to give an additional boost, the boost will include the assessable (via MRI, CT, clinical examination) tumour with a 1 cm margin within the same anatomical compartment as the tumour is located in.

4.1.1.3 Toxicity and stopping rules

Toxicity will be assessed and recorded according to the CCTAE v4.0 acute radiation morbidity scoring criteria.

There is a risk of acute neuropathic pain. If this occurs, the upper border of the beams can be lowered by a few cm or, alternatively attempts to block the sacral nerve roots should be done if possible considering the tumour extent. This usually results in that the pain disappears. If not, treatment should be interrupted. A short period of corticosteroid treatment may help.

4.1.2 Chemotherapy

Neo-adjuvant chemotherapy consists of a combination of capecitabine and oxaliplatin.

Oxaliplatin

The standard dose of oxaliplatin in the CAPOX regimen is 130 mg/m^2 (5 mg/ml concentrate for solution for infusion) in 500 ml glucose 5% i.v. infusion in 30-120 minutes and should *never* be dissolved in NaCl. If FOLFOX is used instead, the oxaliplatin dose is 85 mg/m^{2}.

When prescribing oxaliplatin, the contra-indications, special warnings and interactions, as described in the latest version of the Summary Product Characteristics (SMP) (1B text), should be observed.

Capecitabine

For practical reasons dosing of capecitabine should be rounded to the nearest dose that can be administered using the 150 and 500 mg tablets. When prescribing capecitabine, the contra-indications, special warnings and interactions, as described in the latest version of the SmPC (1B text), should be observed.

Other medication

Anti-emetic prophylaxis with a 5HT3 antagonist and a glucocorticosteroid is required for all patients prior to each oxaliplatin dose.

Other standard supportive therapies should be administered as clinically indicated.

4.1.2.1 Chemotherapy doses and timing

Neoadjuvant chemotherapy

Preferably, chemotherapy will start within 11-18 days after the last day of radiotherapy. However, in case of treatment related diarrhoea or other toxicity, further delay until the toxicity is resolved is allowed till 4 weeks after the last day of radiotherapy. If there are signs of tumour progression during the neo-adjuvant chemotherapy, this treatment should be stopped, and the patient should be evaluated as soon as possible for surgery.

**drug**	**dose**	**frequency**	
Capecitabine	1000 mg/m^2	Twice daily, day 1-14	Every 3 week cycle, in total 4 cycles
Oxaliplatin	130 mg/m^2	Every 3 weeks	

Table 2 *Dose of (neo) adjuvant chemotherapy*

As an alternative to CAPOX, particularly if capecitabine is not well tolerated, mFOLFOX-6 can be used.

4.1.3 Dose modification schedules

4.1.3.1 Capecitabine

The most frequently occurring non-haematologic toxicities are: hand-foot syndrome, asymptomatic hyperbilirubinaemia, diarrhoea, nausea/vomiting (not requiring anti-emetic prophylaxis), abdominal pain, stomatitis, and anorexia.

In case of grade 2-3 hand-foot syndrome, capecitabine dosing should be interrupted until recovery until ≤ grade 1. The omitted doses should not be administered after resuming of treatment, i.e. the total length of each capecitabine treatment period should not exceed 14 days (during induction or reintroduction of MTD chemotherapy).

If painful swelling or erythema of hands or feet occur, emollients are beneficial. Pyridoxin, vitamin B6 50 – 150 mg/day has been reported to be of possible benefit to the patients. Pyridoxin is not licensed for that indication.

Diarrhoea

Prophylactic treatment:

No prophylaxis must be given, especially no loperamide should be administered prophylactically.

In case of diarrhoea grade 2-4, capecitabine intake should be interrupted immediately. Capecitabine can only be restarted when diarrhoea is resolved to grade ≤ 1.

In case of interruption of capecitabine therapy, the omitted doses should not be administered after resuming of treatment, i.e. the total length of each capecitabine treatment period should not exceed 14 days.

Patients experiencing severe diarrhoea should be followed cautiously. In case of risk of dehydration, fluids and electrolytes should be administered. Standard treatment for diarrhoea should be prescribed (i.e. loperamide).

If diarrhoea persists for more than 48 hours despite the recommended loperamide treatment, the patient should be hospitalised for parenteral support. Loperamide may be replaced by other anti-diarrhoeal treatment (e.g. octreotide etc.).

Patients who experience concomitant vomiting or fever or have a performance status > 2 should be hospitalised immediately for i.v. rehydration.

Capecitabine treatment interruption during the cycle

Capecitabine intake must be interrupted in case of ≥ grade 2 non-haematologic toxicity and can be resumed after improvement to ≤ grade 1. During induction treatment the omitted doses should not be administered after resuming of treatment, i.e. the total length of each capecitabine treatment period should not exceed 14 days. In case recovery to ≤ grade 1 does not occur within 2 weeks, the treatment should be discontinued.

Capecitabine dose adaptations for non-haematological toxicity

No dose reduction for the 1st occurrence of grade 2 toxicity, but treatment should be interrupted until recovery of symptoms to grade 0-1. The dose should be reduced 25% relative to the previous cycle at the 2nd occurrence of grade 2 or the occurrence of any grade 3 toxicity. The dose should be reduced 50% relative to the previous cycle at the 3rd occurrence of any grade 2 toxicity or a 2nd occurrence of any grade 3 toxicity or the occurrence of any grade 4 toxicity. Treatment should be discontinued if despite these dose reductions, a given toxicity occurs for a 4th time at grade 2, a 3rd time at grade 3, or a 2nd time at grade 4 (see table 3 below).

	Grade 2	Grade 3	Grade 4
1st occurrence	Interrupt treatment<br>♦ Until symptom recovery to grade 0-1<br>♦ Continue with 100% of the capecitabine dose	Interrupt treatment<br>♦ Until symptom recovery to grade 0-1<br>♦ Continue with 75% of the capecitabine dose	Interrupt treatment<br>♦ Until symptom recovery to grade 0-1<br>♦ Continue with 50% of the capecitabine dose
2nd occurrence	Interrupt treatment<br>♦ Until symptom recovery to grade 0-1<br>♦ Continue with 75% of the capecitabine dose	Interrupt treatment<br>♦ Until symptom recovery to grade 0-1<br>♦ Continue with 50% of the capecitabine dose	Discontinue treatment
3rd occurrence	Interrupt treatment<br>♦ Until symptom recovery to grade 0-1<br>♦ Continue with 50% of the capecitabine dose	Discontinue treatment	
4th occurrence	Discontinue treatment		

Table 3. Dose adaptions of ***capecitabine*** *for non-haematological toxicity.*

In the case of cardiac symptoms (angina, arrhythmia) considered possibly related to capecitabine, CAPOX can be replaced with Nordic FLOX (5-FU 500 mg/m^2 iv and calciumfolinate 100 mg iv days 1 and 2 with oxaliplatin 85 mg/m^2 iv day 1 q 2 weeks) for the corresponding time of neoadjuvant treatment.

4.1.4 Oxaliplatin

The most frequently occurring non-haematologic toxicities are: sensory neuropathy, nausea/vomiting (requiring anti-emetic prophylaxis), diarrhoea, mucositis/stomatitis.

Sensory neuropathy

A 25% dose reduction of oxaliplatin in case of persistent (≥ 14 days) paresthesia or temporary (7-14 days) painful paresthesia or functional impairment. In case of persistent (≥ 14 days) painful paresthesia or functional impairment, oxaliplatin should be omitted until recovery and may be restarted at 50% of the dose. If despite of a 50% dose reduction, neurotoxicity does recur, and oxaliplatin will be discontinued permanently and patients will continue treatment with capecitabine (or 5-FU, see above). In case oxaliplatin infusion is not possible according to this schedule on day 1 of the next cycle, this cycle should not be delayed, and oxaliplatin should be withheld until the following cycle. Acute neurosensory effects (acute laryngeopharyngeal dysesthesia with subjective feelings of dyspnea and dysphagia without signs of bronchospasms or pulmonary abnormalities) have been observed. See also table 4 below.

Sensory neuropathy	Oxaliplatin dose
Non-painful paresthesia ≥ 14 days or<br>temporary (7-14 days) painful paresthesia/functional impairment	25% reduction
Persistent (pain≥ 14 days) painful paresthesia/functional impairment	Omit until recovery, then restart at 50%
Recurrent neurotoxicity after 50% dose reduction	Permanently discontinued

*Table 4. Dose adaptions for **oxaliplatin** for sensory neuropathy (cycles 1 – 6(8))*

Extravasation of oxaliplatin

No severe extravasation reactions have been observed so far with oxaliplatin.

As a general recommendation in the event of extravasation, the following measures are advised (like for any other cytotoxic drug):

1. Stop the infusion immediately.
2. Do not remove the needle or cannula.
3. Aspirate with the same needle as much infiltrated drug as possible from the subcutaneous site.
4. Apply ice to area for 15 to 20 minutes every 4 to 6 hours for the first 72 hours.
5. Watch the area closely during the following days in order to determine whether any further treatment is necessary.

Allergic/ideosyncratic reactions to oxaliplatin

These reactions have been described occurring shortly after oxaliplatin infusion, and a massive cytokine release has been suggested as its cause. In case such a reaction occurs, prophylaxis with steroids ± anti-histamines is indicated.

Dose adaptions for **oxaliplatin and capecitabine** for non-haematological toxicity: see Table 5 below.

Toxicity during previous cycle	Grade	Next dose Oxaliplatin	Next dose Capecitabine
Diarrhoea	3/4	75%	75%/50%
Mucositis	3/4	Full dose	75%/50%
Skin	3/4	Full dose	75%/50%
Hand-foot-syndrome	2-3	Full dose	See Table 3.
Neurotoxicity	See Table 4	See Table 4	Full dose
Other non haematologic toxicities	3/4	75%	75%/50%

Table 5. Dose adjustment relative to the previous cycle for next cycle.

4.1.5 Status of non-haematological toxicity at the start of each treatment cycle.

Non-haematological toxicity should be ≤ grade 1 before start of the next treatment cycle. If these conditions are not met dosing of all drugs should be delayed for a maximum of two weeks until recovery to ≤ grade 1. In case recovery to ≤ grade 1 does not occur within 2 weeks, the treatment will be discontinued. The only exception will be the occurrence of sensory neuropathy induced by oxaliplatin: in case oxaliplatin infusion is not possible after a 2 week delay, the next cycle should not be further delayed, but oxaliplatin should be withheld until the following cycle.

4.1.6 Status of haematological toxicity at the start of each treatment cycle.

Haematological toxicity may be induced by oxaliplatin, and less frequently by capecitabine.

**Neutrophils (10^9/l)**	**WBC (10^9/l)**	**Platelets (10^9/l)**	**Next dose oxaliplatin**	**Next dose capecitabine**
< 0.5 (grade 4) or febrile neutropenia	< 1.0 (grade 4)	< 25 (grade 4)	-25%	No adjustment

*Table 6. Dose adaptations for o**xaliplatin and capecitabine** for haematological toxicity relative to the previous cycle for the next cycle.*

If these toxicities recur after dose reduction for previous toxicity, the next cycle should be given with a 25% dose reduction of capecitabine. If these toxicities occur again, a 50% dose reduction of oxaliplatin should be given. Treatment should be discontinued if these toxicities recur despite these dose reductions.

4.1.7 At the start of each treatment cycle.

WBC and platelet counts should have been recovered to ≥ 3.0 and ≥ 75 x 10^9/L, respectively, before the start of the next treatment cycle. If these conditions are not met dosing should be delayed for a maximum of 2 weeks. If haematological toxicity has not recovered to the above-mentioned values after 2 weeks delay patients will discontinue treatment with chemotherapy.

4.1.8 Permanent discontinuation of individual drugs due to toxicity

If patients experience severe toxicity despite dose reductions which necessitate the discontinuation of individual or all drugs, these patients will remain on study and should be followed for progression of disease according to the specified timelines.

4.1.9 Prophylactic treatments

Anti-emetic prophylaxis

The prophylactic use of a 5HT3 antagonist i.v. is indicated prior to administration of oxaliplatin. Corticosteroids may be added as prophylaxis. All patients should be provided with a prescription for anti-emetics (metoclopramide or 5-HT3 antagonists) and should receive instructions on how to use this medication in case nausea/vomiting occurs at home.

Trombo-embolic prophylaxis

Trombo-embolic prophylaxis can be used according to local protocols during pre-operative treatment, peri-operatively and during adjuvant treatment.

4.2 Surgery

Patients are treated with tromboembolic prophylaxis, antibiotic prophylaxis and bowel preparation according to local protocols. An open or laparoscopic approach may be used.

After entering the abdomen, the liver, the peritoneum and retroperitoneum are screened for metastatic disease. The operation starts with mobilization of the sigmoid from the lateral or medial approach, dependent upon experience of the surgeon, and whether or not the procedure is done open or laparoscopic. Care has to be taken to identify the hypogastric nerves to avoid damage. The vascular supply is ligated. Ligation of the inferior mesenteric artery at its origin from aorta (“high tie”) is not mandatory and ligation the superior rectal artery is considered oncologically adequate. The inferior mesenteric vein is divided at the level of convenience. After the vessels are divided the sigmoid colon is transected. The dissection continues in the avascular plane between the mesentery and the parietal structures leaving the ureter covered by its fascia. The pelvic nerves and the inferior pelvic autonomic nerve plexus are identified and preserved if it is oncologically possible. The anterior dissection should always be carried out anteriorly to the Denonvilliers’fascia. The dissection is carried out keeping the mesorectal fascia intact, ending up with a total mesorectal excision (TME). The resection of the primary tumour is carried out using sharp dissection to encompass the circumference of the mesorectum. In high rectal tumours (>12 cm from the anal verge) a partial mesorectal excision (PME) may be used granted that the distal margin in both the bowel and the mesorectum is at least 5 cm. In mid or low rectal tumours (< 12 cm) a TME down to the pelvic floor has to be performed. When an anterior resection or a Hartmann’s procedure is performed, rectum should be irrigated prior to division of the bowel. If a colo-anal anastomosis is planned for a very low rectal cancer, at least a 1 cm distal margin from the tumour is required. In case of an abdominal perineal resection (APR) in low tumours a perineal resection with the extra-levator technique aiming at a cylindrical specimen without “waisting” is mandatory. In patients with poor bowel function, a Hartmann´s procedure or an inter-sphincteric APR may be used if oncologically safe.

Potentially invaded adjacent structures are resected en bloc with the rectum. This may include small bowel, ureter(s), bladder, vaginal wall and/or uterus and also the sacrum below the level of S3. Thus, patients may require a partial or full pelvic exenteration. Following APR, closure of the perineal wound is up to discretion to each surgeon, but musculocutaneous flaps are advisable. Omental flaps and drains can be used according to surgeon preference. Following an anterior resection, a covering stoma and drains can be used according to surgeon preference.

5 Clinical evaluation, laboratory tests, follow-up

5.1 Before treatment start

5.1.1 Eligibility evaluation

The following studies are required upon entry into the study, maximum 6 weeks prior to start of radiotherapy:

- Physical examination, including blood pressure, ECOG performance score

- Rigid sigmoidoscopy (rectoscopy) or colonoscopy with biopsy of the tumour and a "clean colon" investigation with CT-colonography, barium enema or colonoscopy

- Contrast enhanced multi-detector CT scan of thorax, abdomen and pelvis

- Laboratory tests: haemoglobin, white blood cell count, platelets, bilirubin, ALP, ASAT, ALAT, creatinine, and CEA.

- MRI scan of the pelvis (see Appendix **G** in the RAPIDO protocol).

5.1.2 Obstructing tumours

Patients who present with obstructing tumours may be candidates for a diverting colostomy which can be performed laparoscopically.

5.2 During treatment

5.2.1 Experimental arm (5 x 5 Gy, neo-adjuvant chemotherapy, surgery,

5.2.1.1 Interval between short course radiation and chemotherapy

In case of no or moderate toxicity chemotherapy starts the following week, ideally 11 – 18 days after the last radiation fraction. In case of more than moderate toxicity chemotherapy will be postponed with one week, or longer if necessary (see also Chapter 4.1.1.3)

5.2.1.2 Evaluation during neo-adjuvant chemotherapy

Prior to all cycles (1 to 4):

- ECOG performance status
- Haematology
- Physical examination
- Biochemistry (Na, K, bilirubin, ALP, ALAT, ASAT, creatinine)

5.2.1.3 Re-staging

After the end of chemotherapy (1 – 2 weeks after the last dose) resectability of the primary tumour is evaluated by MRI of the pelvis. Appearance of metastatic disease is evaluated with contrast-enhanced multi-detector CT scan of thorax, abdomen and pelvis, at the end of chemotherapy.

5.2.1.4 Interval between chemotherapy and surgery

After completing the neo-adjuvant chemotherapy, time must be allowed for the patient to recover. Surgery (rectal resection) should be planned within 2 to 4 weeks after the last dose of capecitabine in the last cycle of chemotherapy.

5.3 Stopping rules due to chemotherapy toxicity

This may be the case if severe adverse events are persistent.

A patient should be withdrawn from treatment in any case due to toxicity, if one of the following toxicities persists despite withholding the capecitabine and oxaliplatin for a maximum delay of two weeks:

- Absolute neutrophils count (ANC) < 1.0 and platelets <100 x 10^9/L, respectively
- If the chemotherapy-induced gastrointestinal toxicity does not normalize
- If any other toxicity ≥ grade 3 persists

Toxicity will be assessed and documented according the CTCAE version 4.0. Most common grade 3-4 toxicity are demonstrated in Tables 3, 4, 5, 6, 7 in Chapter **4**.

5.4 Resection and response evaluation

A multidisciplinary team with a panel of radiologist, rectal surgeons, medical-oncologist and radiation-oncologist will evaluate the imaging studies to assess resectability and tumour response. Tumours will be considered resectable unless on imaging:

- T4 tumour with invasion of the sacrum above the level of S3.
- Encasement of lumbosacral nerve root(s)
- Para-aortic pathological nodes (=M1)
- Inguinal lymph nodes (=M1)
- Carcinosis peritonei (=M1)

Special notes on the assessment of target lesions regarding lymph nodes. Lymph nodes identified as target lesions should always have the actual short axis measurement recorded (measured in the same anatomical plane as the baseline examination), even if the nodes regress to below 10mm on study. This means that when lymph nodes are included as target lesions, the ‘sum’ of lesions may not be zero even if complete response criteria are met, since a normal lymph node is defined as having a short axis of <10mm. Case report forms or other data collection methods may therefore be designed to have target nodal lesions recorded in a separate section where, in order

to qualify for CR, each node must achieve a short axis <10mm. For PR, SD and PD, the actual short axis should be given.

5.5 Pathologic evaluation of the rectal cancer resection specimen

Pathological evaluation of the resection specimen will be conducted according to national guidelines and will include standardized workup (see appendix **C** in the RAPIDO protocol) as well as standardized reporting. Key features in the reporting of rectal carcinoma include investigation of depth of tumour invasion and the presence of lymph node involvement. Using these parameters, TNM classification can be assessed. The 7th edition of TNM will be used in this study. In addition, an evaluation of the involvement of circumferential resection margins (CRM) [35], quality of surgery by photo [36] and tumour regression must be done. A circumferential margin of 1 mm or less is considered positive. The exact measurements of the CRM should be given, and, in cases of lymph nodes or tumour deposits being closer to the CRM than the mass of the primary tumour, two separate CRMs should be measured (one of the closest margin and the other one from the primary tumour mass). It should be noted that CRM can only be evaluated postoperatively; preoperatively the evaluation should relate to anatomical structures, like the mesorectal fascia [37].

5.5.1 <u>Quality of resection evaluation</u>

The quality of resection is evaluated at two different levels for APRs (mesorectum as well as anal canal) and at one level for anterior resections or Hartmann’s (mesorectum).

The mesorectal score is based on the surgical plane which is achieved:

- Mesorectal plane (Complete): intact mesorectum with only minor irregularities of smooth mesorectal surface. No defect is deeper than 5 mm, and there is no coning toward the distal margin of the specimen. There is a smooth circumferential resection margin on slicing.

- Intramesorectal plane (Nearly complete): moderate bulk to the mesorectum, but irregularities of the mesorectal surface. Moderate coning of the specimen is allowed. At no site is the muscularis propria visible, with the exception of the insertion of the levator muscles.

- Muscularis propria plane (Incomplete): little bulk to mesorectum with defects down onto muscularis propria and/or very irregular circumferential resection margin.

In analogy, the score of the anal canal is:

- Outside levator plane: This plane has a cylindrical specimen with the levators removed en bloc
- Sphincteric plane: This plane has CRM on the surface of the sphincteric muscular tube, but this is intact.
- Intramuscular/submucosal plane: This plane has perforation or missing areas of muscularis propria indicating entry into the muscular tube at this level

5.5.2 Tumour regression score

Tumour regression is scored using a three-tiered system: no regression, regression and complete response. Complete pathological response is only used after standardized workup of the specimen which includes blocking of the whole tumour area and cutting three levels of each block (at 250 um).

5.6 After the end of treatment: Follow-up

If pre-operatively no complete colonoscopy could be performed a total colonoscopy has to be performed within the first year after treatment. At baseline, 12, 36 and 60 months after date of surgery will be done by taking history, physical examination, ECOG performance score, symptoms according to CTC (see case record forms) and CEA. Follow-up visits with CEA and pulmonary x-ray and ultrasound of the liver or CT of thorax and abdomen should be done after 12, and 36 months (see also Table 8). On indication other diagnostic or imaging techniques (MRI, FDG-PET, colonoscopy) can be used to confirm or detect recurrent or metastatic disease. When recurrent or metastatic disease is detected this time is marked as the time to progression starting from start of radiotherapy. Hereby the disease-free survival can be calculated. After five years, routine follow-up will be ended in case of no evidence of disease after performing a final colonoscopy. More intense follow-up is allowed if this is routinely done.

If a centre participation in the quality-of-life (QoL) evaluation after 3 years in the RAPIDO study, it is desirable to have this done also in LARCT-US.

5.6.1 Requirements for Follow-Up

Months since date of surgery		12	36	60
History, incl. morbidity assessment		X	X	X
Physical Examination		X	X	X
CEA		X	X	X
X-thorax & US-liver or CT thorax-abdomen		X	X	
Colonoscopy				X
Quality-of-life (optional)			X	

Table 8: follow-up scheme. More intense follow-up is allowed if this is routinely done.

5.6.2 Assessment of Recurrent Disease

Evidence of recurrent disease is accepted when one of the following criteria is present:

- Positive histology or cytology of adenocarcinoma, compatible with the primary tumour in any location.

- Liver metastases on Ultrasound and/or (PET)CT.
- Lung metastases on X-ray and/or (PET)CT or MRI scan.
- Bone metastases on X-ray and/or bone-scintigraphy and/or MRI
- Brain metastases on MRI
- Distant lymph node metastases
- Changes in soft tissue outlines on (PET)CT or MRI– pelvis in combination with an increased CEA to differentiate from fibrosis. Parameters for Recurrent Disease

The following parameters will be recorded and studied:

- Loco-regional recurrence site and date (local within the pelvis).

- Distant recurrence site and date (outside the pelvis).

- Cause of death: local failure, local failure and metastases, metastases only, complications due to treatment, intercurrent disease and unknown cause.

5.7 Treatment Summary table

	Baseline	WEEK									
**Required Investigations**		**1**	**2**	**3-4**	**6-7**	**9-10**	**12-13**			**16-17**	**17-20**
Physical examination	x			x							x
ECOG performance score	x			x	x	x	x				x
Tumour related symptoms	x			x	x	x	x				x
Blood Pressure	x			x							x
Haematology[1]	x			x	x	x	x				x
Biochemistry[2]	x			x							x
CEA[3]	x										
CT thorax-abdomen-pelvis	x									x	
MRI pelvis	x									x	
Colonoscopy/rectoscopy[4]	x										
Toxicity evaluation			x	x	x	x	x				x
Radiotherapy		x									
Oxaliplatin				x	x	x	x				
Capecitabine				x	x	x	x				
Surgery											x

1) Hb, WBC count, , platelet count
At baseline: < 72 hour prior to start of chemotherapy

2) Na, K, creatinin, ALP, ALAT, ASAT, , bilirubin
At baseline: < 72 hour prior to start of chemotherapy

3) Within 5 weeks prior to start of radiotherapy

4) Biopsy taken

**Treatment/drug**	**dose**	**frequency**
Radiotherapy	5x5Gy	week 1 day 1-5
Capecitabine	1000 mg/m^2	b.i.d. day 1-14 every 3 week cycle starting at week 3
Oxaliplatin	130 mg/m^2	day 1 every 3 week cycle starting at week 3

6 Criteria of evaluation

6.1 Definitions

Disease-free survival

Disease-free survival will be computed as the time between start of radiotherapy and either local or distant relapse or death caused by the rectal carcinoma whichever comes first. In case of non-rectal cancer related death patients will be censored at date of death. In case of a second primary tumour patients will be censored at the date of diagnosis of the second primary tumour. Patients lost to follow-up will be censored the last date of patient visit.

Toxicity

All patients will be evaluable for toxicity from the time of their first treatment. Toxicity (acute and late) will be assessed and documented according the CTCAE version 4.0. Adverse events and serious adverse events will be reported as described in section 8.4.

Fraction of radical resection (CRM > 1 mm)

Negative CRM will be evaluated according the pathology protocol described by Quirke [36].

Complete pathological response (pCR).

pCR evaluation is done by the method described in the pathology Appendix **C**.

Neoadjuvant rectal score (NAR)

As described in the Background, a novel score will be an additional way to evaluate the results of the study and compare with the results of previous and ongoing trials testing different neo-adjuvant treatments.

Local recurrence

Local recurrence is described as relapse of tumour in the pelvic region.

Distant relapse

Distant relapse is described as relapse of tumour outside the pelvic region. This will be assessed by clinical investigation and imaging studies. Special attention has to be made on the liver and lung since these are the predominant side of metastases.

Local control

Local control will be computed as the time between randomization and local relapse. If the primary tumour cannot be removed macroscopically radically, the time to local failure is zero months. Patients who died or are lost to follow-up without evidence of local relapse are censored at the date of death or the last date of patient visit.

Overall survival

Overall survival will be computed as the time between randomization and all causes of death. Patients lost to follow-up will be censored the last date of patient visit. In case of a second primary tumour patients will be censored at the date of diagnosis of the second primary tumour. Follow-up is described in Chapter **6**.

6.2 Statistical considerations

6.2.1 Sample size

No specific considerations have been taken since this is intended to be a trial that is run between the end of the preceding randomized RAPIDO trial prior to the results of the trial are available or another trial, like the planned CREATE, has been initiated. Since this time is likely to be in the order of two years, it can be expected that about 60 patients will be included. This number is sufficient to allow a proper evaluation of the primary outcome and a good evaluation of the toxicity from the treatment in relation to that seen in other trials. It is possible to make a more proper estimation of sample size if it turns out that it is motivated to continue inclusion further prior to a more conclusive trial will be initiated.

All efficacy analyses will be based on intention-to-treat. Per-protocol analyses will be performed as secondary analyses.

Safety analyses will be based on treatment received and will include only eligible patients.

Survival curves for disease-free survival and overall survival will be constructed using the method of Kaplan-Meier. Cumulative incidence of local recurrence will be computed accounting for death as competing risk. Differences in survival between subgroups will be tested with the log-rank test. Hazard ratios and 95% confidence intervals (CI) will be computed using Cox regression.

All tests will be two-sided.

A table will present the completion rate of the neo-adjuvant treatment, pCR frequency and percentages, fraction of patients with a radical resection with 90 and 95% CI.

Frequency and percentages for toxicity will be presented according to the CTCAEv4.0 (see appendix **D** in the RAPIDO protocol). All proportions will be presented with 95% CI.

6.3 Interim Analyses

No interim analysis is planned

7 Translational research

Proteomics, genomics, and circulating tumour cell analyses of plasma and tumour tissue along the treatment schedule may provide insight in biomarkers associated with response and prognosis. A tissue block (or two-three cores for tissue microarray, TMA) will be collected from the preoperative biopsy (if sufficient material is available) and from the operative specimen (See appendix C). Optional collection of fresh tissue for freezing and blood samples include:

- Tumour biopsy (at time of colonoscopy), and directly after surgical resection of the rectum, stored at -80˚C.

- No specific blood samples will be taken for the study. It is expected that most patients will be included in the U-CAN project. Blood samples are then taken prior to the start of therapy at diagnosis, after the neo-adjuvant treatment prior to surgery, 6-8 weeks after surgery and 1 and 3 years after during routine follow-up visits.

Patient registration

8 Forms and procedures for collecting data

The case record forms (CRF's) for this study are available on paper and electronically. They are the same as used in the RAPIDO trial with appropriate modifications. They are divided in different numbered sections. All CRF's are identified by the patient´s study number and month and year of birth. All CRF's have to be signed and dated by the person filling in the form.

All CRF's allow registration of optional collection of tissue or plasma for translational research. A logistical form will be kept up to date with all planned clinic appointments and admissions, scheduled studies and treatments.

8.1 Case report forms (modified from the RAPIDO trial)

- F01 Inclusion Form
- F02 History and Staging Form
- F03 Baseline Radiology Form
- F04 Radiotherapy Form
- F05a Pre-operative CAPOX Form
- F05b Pre-operative FOLFOX Form
- F06 Restaging Radiology Form
- F07 Surgery/Post-surgery Form
- F08 Pathology Form
- F11 Follow-up Form
- F12 Recurrence Form
- F13 End of Pre-operative Treatment Form
- F20 Death Form
- F30 Adverse Events/SAE Form
- F50 Comment Form

8.2 Data flow

Paper CRFs will be filled in by treating physicians or data managers at all participating centers and departments.

Reporting adverse events

8.3 Section 10 WHO event

In accordance to section 10, subsection 1, of the WMO, the investigator will inform the subjects and the reviewing accredited METC if anything occurs, on the basis of which it appears that the disadvantages of participation may be significantly greater than was foreseen in the research proposal. The study will be suspended pending further review by the accredited METC, except insofar as suspension would jeopardise the subjects' health. The investigator will take care that all subjects are kept informed.

8.4 Adverse events and serious adverse events

NOTE In this study, the following events are not reported as an AE or SAE:

- planned surgery (e.g. stoma removal)
- planned hospitalisation (e.g. for administering chemotherapy) or recurrences.
- For recurrences, the CRF "new primary / recurrences" has to be filled in;
- death due to progression of disease;

Adverse events are defined as any undesirable experience occurring to a subject during the study, whether or not considered related to [the investigational product / the experimental treatment. Only adverse events reported spontaneously by the subject or observed by the investigator or his staff of grade 3 or 4 will be recorded.

A serious adverse event is any untoward medical occurrence or effect that at any dose:

- results in death;
- is life threatening (at the time of the event);
- requires hospitalisation or prolongation of existing inpatients' hospitalisation;
- results in persistent or significant disability or incapacity;
- is a congenital anomaly or birth defect;
- is a new event of the trial likely to affect the safety of the subjects, such as an unexpected outcome of an adverse reaction, lack of efficacy of an IMP used for the treatment of a life-threatening disease, major safety finding from a newly completed animal study, etc.

SAEs that result in death or are life threatening should be reported expedited. The expedited reporting will occur not later than 7 days after the responsible investigator has first knowledge of the adverse reaction. This is for a preliminary report with another 8 days for completion of the report.

All SAEs, irrespective of relationship to the study treatment must be reported to the Datacentre. The SAE report should include the investigator's assessment of causality. If follow-up information changes the investigator's assessment of causality, this should be noted on the SAEs occurring within 30 days after discontinuation of the study treatment should be reported.

8.5 Suspected unexpected serious adverse reactions (SUSAR)

Adverse reactions are all untoward and unintended responses to an investigational product related to any dose administered.

Unexpected adverse reactions are adverse reactions, of which the nature, or severity, is not consistent with the applicable product information (e.g. Investigator's Brochure for an unapproved IMP or Summary of Product Characteristics (SPC) for an authorised medicinal

product).

8.6 Follow-up of adverse events

All adverse events will be followed until they have abated, or until a stable situation has been reached. Depending on the event, follow up may require additional tests or medical procedures as indicated, and/or referral to the general physician or a medical specialist.

9 Quality assurance

9.1 Control of data consistency

Data for this study will be recorded via using Case Report Forms (CRF). On-site quality control

A monitoring committee will be appointed which will perform monitoring every 6 months after start of this trial.

When necessary regular visits by research nurses, data managers of the regional cancer center or monitoring committee members will be organized.

9.2 Audits

The investigator, by accepting to participate to this protocol, agrees to co-operate fully with any quality assurance visit undertaken by third parties, including representatives from the “Sponsor”, national and/or foreign regulatory authorities or company supplying the product under investigation, as well as to allow direct access to documentation pertaining to the clinical trial (including CRFs, source documents, hospital patient charts and other study files) to these authorized individuals.

9.3 Review of pathology

In order to optimize pathology quality, review of pathology will be performed after inclusion of the last patient. A committee of experienced rectal cancer pathologists will be appointed. This board will review biopsies and resected rectal cancer specimens according to the pathology protocol described in section 6 and Appendix C (see the RAPIDO protocol).

9.4 Other review procedures

In order to optimize pre-operative staging, radiology review will be performed after the inclusion of the last patient. A committee of experienced rectal cancer radiologists will be appointed to review all pre-operative CT and MRI scans.

10 Ethical considerations

10.1 Patient protection

The responsible investigator will ensure that this study is conducted in agreement with most recent version of the Declaration of Helsinki and with the laws and regulations of the country.

The protocol has been written, and the study will be conducted according to the ICH Harmonized Tripartite Guideline for Good Clinical Practice.

The protocol will be approved by the Local Ethics Committee.

10.2 Subject identification

The investigator must assure that patients' anonymity will be maintained and that their identities are protected from unauthorized parties. On CRFs or other documents submitted to the data centre, patients should only be identified by the identification code and month and year of birth. The investigator and each investigator in each participating hospital should keep a patient enrolment log showing codes, names and addresses.

10.3 Informed consent

All patients will be informed of the aims of the study, the possible adverse events, the procedures and possible hazards to which he/she will be exposed. They will be informed as to the strict confidentiality of their patient data, but that their medical records may be reviewed for trial purposes by authorized individuals other than their treating physician.

It will be emphasized that the participation is voluntary and that the patient is allowed to refuse further participation in the protocol whenever he/she wants. This will not prejudice the patient's subsequent care. Documented informed consent must be obtained for all patients included in the study before they are registered in the study. This must be done in accordance with the national and local regulatory requirements.

For European Union member states, the informed consent procedure must conform to the ICH guidelines on Good Clinical Practice. This implies that "the written informed consent form should be signed and personally dated by the patient".

11 Publication policy

The trial will be published after completion of the inclusion and completion of follow-up of patients with respect to results regarding the primary and secondary endpoints. The main results regarding the primary and secondary endpoints have to be published first, compared to publication of results of side-studies. The principal investigators will be first author and/or last authors of main papers based on this study. In case of papers of side results authors have to be appointed by the writing committee based on the topic studied and investigators involved.

References

1. Bujko, K., et al., *Long-course oxaliplatin-based preoperative chemoradiation versus 5 x 5 Gy and consolidation chemotherapy for cT4 or fixed cT3 rectal cancer: results of a randomized phase III study.* Ann Oncol, 2016. **27**(5): p. 834-42.
2. Cancer Incidence in Sweden 2014, www.socialstyrelsen.se 2016.
3. Lindegaard, J., et al., *Whither Papillon? Future directions for contact radiotherapy in rectal cancer.* Clin Oncol (R Coll Radiol), 2007. **19**(9): p. 738-41.
4. Habr-Gama, A., et al., *Preoperative chemoradiation therapy for low rectal cancer. Impact on downstaging and sphincter-saving operations.* Hepatogastroenterology, 2004. **51**(60): p. 1703-7.
5. Heald, R.J., E.M. Husband, and R.D. Ryall, *The mesorectum in rectal cancer surgery--the clue to pelvic recurrence?* Br J Surg, 1982. **69**(10): p. 613-6.
6. Glimelius, B., *Multidisciplinary treatment of patients with rectal cancer: Development during the past decades and plans for the future.* Ups J Med Sci, 2012. **117**(2): p. 225-36.

7. Swedish Rectal Cancer Trial, *Improved survival with preoperative radiotherapy in resectable rectal cancer.* N Engl J Med, 1997. **336**: p. 980-987.
8. Kapiteijn E, et al., *Preoperative radiotherapy in combination with total mesorectal excision improves local control in resectable rectal cancer. Report from a multicenter randomized trial.* New Engl J Med, 2001. **345**: p. 638-646.
9. Sebag-Montefiore, D., et al., *Preoperative radiotherapy versus selective postoperative chemoradiotherapy in patients with rectal cancer (MRC CR07 and NCIC-CTG C016): a multicentre, randomised trial.* Lancet, 2009. **373**(9666): p. 811-20.
10. Gerard, J.P., et al., *Preoperative radiotherapy with or without concurrent fluorouracil and leucovorin in T3-4 rectal cancers: results of FFCD 9203.* J Clin Oncol, 2006. **24**(28): p. 4620-5.
11. Bosset, J.F., et al., *Chemotherapy with preoperative radiotherapy in rectal cancer.* N Engl J Med, 2006. **355**(11): p. 1114-23.
12. Braendengen M, et al., *A randomized phase III study (LARCS) comparing preoperative radiotherapy alone versus chemoradiotherapy in non-resectable rectal cancer.* J Clin Oncol, 2008. **26**: p. 3687-3694.
13. Radu C, et al., *Short course preoperative radiotherapy with delayed surgery in rectal cancer - a retrospective study.* Radiother Oncol, 2008. **87**: p. 343-9.
14. Hatfield, P., et al., *Short-course radiotherapy, with elective delay prior to surgery, in patients with unresectable rectal cancer who have poor performance status or significant co-morbidity.* Radiother Oncol, 2009. **92**(2): p. 210-4.
15. Pettersson, D., et al., *Interim analysis of the Stockholm III trial of preoperative radiotherapy regimens for rectal cancer.* British Journal of Surgery, 2010. **97**(4): p. 580-7.
16. Pettersson, D., et al., *Tumour regression in the randomized Stockholm III Trial of radiotherapy regimens for rectal cancer.* Br J Surg, 2015. **102**(8): p. 972-8.
17. Bujko, K., et al., *Postoperative chemotherapy in patients with rectal cancer receiving preoperative radio(chemo)therapy: A meta-analysis of randomized trials comparing surgery +/- a fluoropyrimidine and surgery + a fluoropyrimidine +/- oxaliplatin.* Eur J Surg Oncol, 2015. **41**(6): p. 713-723.
18. Breugom, A.J., et al., *Adjuvant chemotherapy after preoperative (chemo)radiotherapy and surgery for patients with rectal cancer: a systematic review and meta-analysis of individual patient data.* Lancet Oncol, 2015. **16**(2): p. 200-7.
19. Breugom, A.J., et al., *Adjuvant chemotherapy for rectal cancer patients treated with preoperative (chemo)radiotherapy and total mesorectal excision: a Dutch Colorectal Cancer Group (DCCG) randomized phase III trial.* Ann Oncol, 2015. **26**(4): p. 696-701.
20. Anderson, C., et al., *PET-CT Fusion in Radiation Management of Patients with Anorectal Tumors.* Int J Radiat Oncol Biol Phys, 2007. **69**(1): p. 155-62.
21. Smith N and Brown G, *Preoperative staging in rectal cancer.* Acta Oncol, 2008. **47**: p. 20-31.
22. Smith NJ, et al., *Prognostic significance of magnetic resonance imaging-detected extramural vascular invasion in rectal cancer.* Br J Surg, 2008. **95**: p. 229-36.
23. Matthiessen, P., et al., *Defunctioning stoma reduces symptomatic anastomotic leakage after low anterior resection of the rectum for cancer: a randomized multicenter trial.* Ann Surg, 2007. **246**(2): p. 207-14.
24. Sauer, R., et al., *Preoperative versus postoperative chemoradiotherapy for rectal cancer.* N Engl J Med, 2004. **351**(17): p. 1731-40.

25. Chau, I., et al., *Neoadjuvant capecitabine and oxaliplatin followed by synchronous chemoradiation and total mesorectal excision in magnetic resonance imaging-defined poor-risk rectal cancer.* J Clin Oncol, 2006. **24**(4): p. 668-74.
26. Boige V and et al, *Final results of a randomized trial comparing 5-fluorouracil/F)/cisplatin(P) to surgery alone in adenocarcinoma of stomach and lower esophagus: FNLCC ACCORD07-FFCD 9703 trial.* J Clin Oncol. ASCO Annual Meeting Proceedings 2007, 25S (No 18S), 2007.
27. Nordlinger, B., et al., *Perioperative chemotherapy with FOLFOX4 and surgery versus surgery alone for resectable liver metastases from colorectal cancer (EORTC Intergroup trial 40983): a randomised controlled trial.* Lancet, 2008. **371**(9617): p. 1007-16.
28. Calabro, F. and C.N. Sternberg, *Neoadjuvant and Adjuvant Chemotherapy in Muscle-Invasive Bladder Cancer.* Eur Urol, 2009. **55**: p. 348-358.
29. van Dijk, T.H., et al., *Evaluation of short-course radiotherapy followed by neoadjuvant bevacizumab, capecitabine, and oxaliplatin and subsequent radical surgical treatment in primary stage IV rectal cancer.* Ann Oncol, 2013. **24**(7): p. 1762-9.
30. Glimelius, B., *Optimal time intervals between pre-operative radiotherapy or chemoradiotherapy and surgery in rectal cancer?* Front Oncol, 2014. **4**: p. 50:1-10.
31. Bujko K, et al., *Long-term results of a randomised trial comparing preoperative short-course radiotherapy vs preoperative conventionally fractionated chemoradiation for rectal cancer.* Br J Surg, 2006. **93**: p. 1215-1223.
32. Ngan S and et al, *A randomized trial comparing local recurrence (LR) rates between short-course (SC) and long-course (LC) preoperative radiotherapy (RT) for clinical T3 rectal cancer: An intergroup trial (TROG, AGITG, CSSANZ, RACS).* J Clin Oncol 28:15s (suppl; abstr 3509), 2010.
33. Pettersson, D., et al., *Interim analysis of the Stockholm III trial of preoperative radiotherapy regimens for rectal cancer.* Br J Surg, 2010. **97**(4): p. 580-7.
34. Veenhof, A.A., et al., *Preoperative radiation therapy for locally advanced rectal cancer: a comparison between two different time intervals to surgery.* Int J Colorectal Dis, 2007. **22**(5): p. 507-13.
35. Nagtegaal, I.D. and P. Quirke, *What is the role for the circumferential margin in the modern treatment of rectal cancer?* J Clin Oncol, 2008. **26**(2): p. 303-12.
36. Quirke, P., et al., *Effect of the plane of surgery achieved on local recurrence in patients with operable rectal cancer: a prospective study using data from the MRC CR07 and NCIC-CTG CO16 randomised clinical trial.* Lancet, 2009. **373**(9666): p. 821-8.
37. Glimelius, B., et al., *Mesorectal fascia instead of circumferential resection margin in preoperative staging of rectal cancer.* J Clin Oncol, 2011. **29**(16): p. 2142-3.

Appendix E - Patient Information and Informed Consent LARCTUS

INFORMED CONSENT FORM

Patientinformation LARCT-US studien

Du tillfrågas härmed om att delta i en studie vid lokalt avancerad ändtarmscancer. Studien undersöker effekt och säkerhet av en kombination av strålning under en vecka följt av cytostatika (cellgifter) under 3 månader före operationen.

Bakgrund och syfte

Den utredning som gjorts visar att du har en tumör i ändtarmen som växer utanför tarmväggen, att det eventuellt finns växt över på andra organ eller spridning i tarmnära lymfkörtlar men att det inte finns tecken till spridning till andra organ, s.k. fjärrmetastaser. Standardbehandlingen vid ändtarmscancer med denna utbredning (benämns lokalt avancerad ändtarmscancer) är en kombination av strålning, cytostatika under cirka 5 veckor följt av operation 6 – 8 veckor senare. Det har visat sig att strålningen har bäst effekt och minst biverkningar om den ges innan operationen. Strålbehandlingen kan också ges i högre doser varje dag under en vecka. Andra studier vid ändtarmscancer som är mindre avancerade än din tumör har visat att en veckas bestrålning har samma effekt på tumören som strålning under 5 veckor med cytostatika. När tumören växer så som i ditt fall rekommenderas alltid att man väntar minst 6 - 8 veckor efter avslutad strålbehandling innan operationen så att tumören kan krympa och på så sätt bli lättare att i sin helhet opereras bort.

Dessutom rekommenderas ofta cytostatika för att ytterligare minska risken för återfall i övriga delar av kroppen. Av hävd har denna tilläggscytostatika getts efter operationen, men andra studier har visat att effekten är bättre om den ges före operationen. I denna studie ges cytostatikabehandlingen efter strålbehandlingen, men före operationen. Den cytostatika som normalt ges är en kombination av två läkemedel, capecitabin (en fluoropyrimidin som ges som tabletter) och oxaliplatin (kombination benämnd CAPOX). I en nyligen avslutad studie genomförd vid flera sjukhus i många länder har den korta strålbehandlingen under en vecka följts av cytostatika under 18 veckor (sex omgångar av CAPOX-kombinationen). Erfarenheterna av behandlingen är mycket goda. I en annan studie (gjord av en polsk forskargrupp) vid lokalt avancerad ändtarmscancer gavs samma strålbehandling men bara motsvarande två omgångar cytostatika, också med tillsynes goda behandlingsresultat. För att ha god effekt på misstänkt spridd sjukdom finns det dock bristande kunskap om positiv effekt av kortare behandlingar än 12 veckor, motsvarande fyra omgångar CAPOX.

Syftet med denna studie är att undersöka hur effektiv kort strålbehandling följd av fyra omgångar CAPOX är som förbehandling inför operation av lokalt avancerad ändtarmscancer. Det mått som i första hand används för att värdera effekten är andelen patienter hos vilka tumören vid operationen visar sig helt ha försvunnit. Därutöver insamlas data som belyser behandlingens biverkningar.

Denna studie

I denna studie ges en kombination av kort strålbehandling under en vecka följt av cytostatika med CAPOX kombinationen under tre månader, dvs. fyra omgångar. Operationen sker efter ytterligare 3 - 4 veckor.

Behandlingen:
Först ges strålning under 5 dagar. Cirka 10 till 18 dagar senare påbörjas cytostatikabehandlingen med en kombination av capecitabin och oxaliplatin, dvs. CAPOX-behandling. Totalt ges 4 omgångar var tredje vecka. Dag 1 i varje omgång ges oxaliplatin som dropp under ca 1 timme och på kvällen samma dag startas capecitabin i tablettform. Capecitabin tas därefter morgon och kväll i 2 veckor följt av en veckas uppehåll. Efter en paus på 3 till 4 veckor efter sista CAPOX-omgången görs operationen om inte ny röntgen med magnetkamera och datortomografi dessförinnan gör att annan behandling rekommenderas.

Genomförande

Undersökningar och provtagningar skiljer sig inte från dem som görs i ordinarie vård. Före studiebehandlingen görs således röntgenundersökningar med magnetkamera och datortomografi och blodprover tas. Nya blodprover tas sedan inför varje cytostatika-behandling som också föregås av läkarbesök för att värdera biverkningar. Före operationen görs ny röntgen för att mäta ändtarmtumörens storleksändring och för att kontrollera att ingen spridning av sjukdomen skett.

Biverkningar

Alla cancerbehandlingar kan ha biverkningar. Dessa varierar dock påtagligt från patient till patient, vissa får nästan inga biverkningar alls medan några får betydligt mer och ibland även allvarliga biverkningar. Den korta strålbehandlingen under en vecka ger oftast få eller inga biverkningar. Veckan efteråt kan några patienter känna sig trötta, få diarré och må något illa. Mycket ovanligt är att dessa biverkningar blir svåra och någon gång kan det krävas inläggning på sjukhus. Strålbehandlingen kan också ge övergående smärtor i bakre delen av ryggen och ner i benen.

Kombinationen av capecitabin och oxaliplatin (CAPOX) kan ge illamående, kräkningar, diarré, påverkan på blodbilden med blodbrist, brist på vita blodkroppar och risk för infektioner och brist på blodplättar med risk för blödning. Capecitabin kan också ge påverkan på munslemhinna, handflator och fotsulor liksom på hjärtat med rytmrubbningar. Dessa biverkningar är i allmänhet övergående. Håravfall är ovanligt.

Oxaliplatin ger ofta en påverkan på nerver som yttrar sig i början som överkänslighet mot kyla med stickningar och domningar i fingrar, svalg och tår. Dessa besvär har en tendens att bli något värre för varje kur och kan någon gång bli besvärande. Det finns en risk att dessa biverkningar blir bestående också lång tid, ibland flera år efter avslutad behandling. Det är viktigt att du informerar läkaren om du får dessa besvär så att man i tid kan minska på oxaliplatindosen och kanske mot slutet avbryta just den delen av behandlingen. Man kan dock förvänta sig att dessa långsiktiga biverkningar blir mindre efter 3 månaders behandling som i denna studie jämfört med efter 6 månaders behandling vilket tidigare varit rutin när man gett tilläggsbehandling med cytostatika.

Även operationen kan ha biverkningar. Beroende på hur stor din tumör är och var den sitter kan operationen innebära ett mer eller mindre stort ingrepp. Ibland behöver enbart en del av ändtarmen tas bort, ibland även slutmuskeln med permanent stomi som följd. Ibland kan det finnas behov av att ta bort också intilliggande organ som livmodern hos kvinnor, prostatakörteln eller urinblåsan hos män. Omfattningen av ingreppet på just dig

kommer din kirurg att informera dig om. Fullt ut kan man inte bestämma hur omfattande operationen blir förrän strax innan själva operationen. De biverkningar som en operation kan medföra både på kort och lång sikt beskrivs inte här men din kirurg kan ge mer information.

Möjliga för- och nackdelar med att delta i studien

Syftet med studien är att undersöka hur väl behandlingen tolereras av patienterna och hur stor påverkan blir på tumören, dvs. om detta sätt att ge behandlingarna i förhållande till varandra har för- eller nackdelar. Genom detta får vi ny kunskap av betydelse för framtida patienter. Samtidigt kommer du att få den behandling som vi idag betraktar som den mest effektiva, nämligen denna kombination av strålning, cytostatika och operation.

Om du väljer att inte delta i denna studie kommer du att få den behandling som hittills varit standardbehandling, d v s strålning under ca 5 veckor i kombination med capecitabin följt av operation 6 – 8 veckor senare. Beroende på resultatet av den behandlingen kan också cytostatikabehandling ges efter operationen. Ditt deltagande i studien är helt frivilligt och du kan när som helst avbryta ditt deltagande utan att behöva ange några skäl.

Vad händer med mina uppgifter?

För denna studie är Region Uppsala personuppgiftsansvarig. Enligt EU:s direktiv har du rätt att kostnadsfritt få ta del av de uppgifter om dig som hanteras i studien, och vid behov få eventuella fel rättade. Du kan också begära att uppgifter om dig raderas samt att behandlingen av dina personuppgifter begränsas. Om du vill ta del av uppgifterna ska du kontakta ansvarig för studien, professor Bengt Glimelius, Onkologikliniken, Akademiska sjukhuset, 751 85 Uppsala, telefon 018-611 55 13. Region Uppsala har ett dataskyddsombud. Postadressen är: Dataskyddsombudet, Region Uppsala, Storgatan 27, 751 25 Uppsala. E-postadress: dataskyddsombud@regionuppsala.se. Delar av din patientjournal kommer att rapporteras i patientformulär (Case Record Form, CRF) som är en del av studiedatabasen. I studiedatabasen är alla personuppgifter kodade. Dina uppgifter behandlas sedan tillsammans med alla andra patienters med datateknik för statistiska beräkningar.

För att säkerställa att de uppgifter som insamlas är korrekta, kan dessa komma att jämföras med uppgifterna i din patientjournal. Denna uppgift utförs av speciellt utbildad person (monitor) som har tystnadsplikt. Uppgifterna kan också komma att kontrolleras av läkemedelsmyndigheter. Resultaten av studien kommer att publiceras i medicinsk facklitteratur i form av kurvor och tabeller. Det innebär att ditt namn och personnummer inte lämnas ut. Dina personuppgifter förvaras med en kod som endast din läkare kan identifiera. Efter studien kommer koden att förvaras i enlighet med gällande lag.

Frivillighet

Deltagande i studien är helt frivilligt och du kan när som helst avbryta ditt deltagande utan att behöva ange något skäl. Om du väljer att avbryta studien kommer det inte att påverka ditt fortsatta omhändertagande på kliniken.
Om det är något ytterligare du undrar över är du alltid välkommen att kontakta huvudansvarig för studien professor Bengt Glimelius, Onkologiska kliniken, Akademiska

sjukhuset i Uppsala, telefon 018-611 55 13 eller din ansvarige läkare eller forskningssjuksköterska:

Ansvarig läkare: ______________________________

Telefon: ______________________________

Forskningssjuksköterska: ______________________________

Telefon: ______________________________

Patientens samtycke till att delta i studien

Jag har muntligen informerats om studien och tagit del av ovanstående skriftliga information. Jag är medveten om att mitt deltagande i studien är fullt frivilligt, och att jag när som helst och utan närmare förklaring kan avbryta mitt deltagande utan att det påverkar mitt omhändertagande.
Jag är dessutom medveten om att ansvarig läkare kan avbryta mitt deltagande, om det anses olämpligt att jag fortsätter.
Jag har haft möjlighet att fråga min läkare om risker, fördelar och alternativa behandlingsmöjligheter.

Tillgång till patientjournal

Jag har informerats om och samtycker till att berörd forskningssjuksköterska/monitor och eventuell läkemedelskontrollmyndighet får jämföra de i studien rapporterade uppgifterna med de uppgifter som finns i min patientjournal under förutsättning att information som därvid blir tillgänglig inte förs vidare.

Datum

Patientens underskrift

Namnförtydligande

Ansvarig prövares försäkran

Undertecknad har gått igenom och förklarat studiens syfte för patienten. Patienten har haft möjlighet att ställa frågor och fått dem besvarade. Patienten har även fått en kopia av patientinformationen.

Datum

Ansvarig läkares underskrift

Namnförtydligande
